# Physical pictures of rotation mechanisms of F_1_- and V_1_-ATPases: Leading roles of translational, configurational entropy of water

**DOI:** 10.3389/fmolb.2023.1159603

**Published:** 2023-06-09

**Authors:** Satoshi Yasuda, Tomohiko Hayashi, Takeshi Murata, Masahiro Kinoshita

**Affiliations:** ^1^ Department of Chemistry, Graduate School of Science, Chiba University, Chiba, Japan; ^2^ Department of Quantum Life Science, Graduate School of Science, Chiba University, Chiba, Japan; ^3^ Membrane Protein Research and Molecular Chirality Research Centers, Chiba University, Chiba, Japan; ^4^ Interdisciplinary Program of Biomedical Engineering, Assistive Technology and Art and Sports Sciences, Faculty of Engineering, Niigata University, Niigata, Japan; ^5^ Institute of Advanced Energy, Kyoto University, Kyoto, Japan; ^6^ Center for the Promotion of Interdisciplinary Education and Research, Kyoto University, Kyoto, Japan

**Keywords:** ATP-driven protein, linear molecular motor, rotary molecular motor, ATP hydrolysis, ATP synthesis, statistical mechanics, protein structure, water entropy

## Abstract

We aim to develop a theory based on a concept other than the chemo-mechanical coupling (transduction of chemical free energy of ATP to mechanical work) for an ATP-driven protein complex. Experimental results conflicting with the chemo-mechanical coupling have recently emerged. We claim that the system comprises not only the protein complex but also the aqueous solution in which the protein complex is immersed and the system performs essentially no mechanical work. We perform statistical-mechanical analyses on V_1_-ATPase (the A_3_B_3_DF complex) for which crystal structures in more different states are experimentally known than for F_1_-ATPase (the α_3_β_3_γ complex). Molecular and atomistic models are employed for water and the structure of V_1_-ATPase, respectively. The entropy originating from the translational displacement of water molecules in the system is treated as a pivotal factor. We find that the packing structure of the catalytic dwell state of V_1_-ATPase is constructed by the interplay of ATP bindings to two of the A subunits and incorporation of the DF subunit. The packing structure represents the nonuniformity with respect to the closeness of packing of the atoms in constituent proteins and protein interfaces. The physical picture of rotation mechanism of F_1_-ATPase recently constructed by Kinoshita is examined, and common points and differences between F_1_- and V_1_-ATPases are revealed. An ATP hydrolysis cycle comprises binding of ATP to the protein complex, hydrolysis of ATP into ADP and Pi in it, and dissociation of ADP and Pi from it. During each cycle, the chemical compounds bound to the three A or β subunits and the packing structure of the A_3_B_3_ or α_3_β_3_ complex are sequentially changed, which induces the unidirectional rotation of the central shaft for retaining the packing structure of the A_3_B_3_DF or α_3_β_3_γ complex stabilized for almost maximizing the water entropy. The torque driving the rotation is generated by water with no input of chemical free energy. The presence of ATP is indispensable as a trigger of the torque generation. The ATP hydrolysis or synthesis reaction is tightly coupled to the rotation of the central shaft in the normal or inverse direction through the water-entropy effect.

## 1 Introduction

Unveiling the mechanism of functional expression of an ATP-driven protein or protein complex is a most challenging subject in bioscience (Suzuki, 2018). It functions through the ATP hydrolysis cycle composed of binding of ATP to it, hydrolysis of ATP into ADP and Pi in it, and dissociation of ADP and Pi from it. Paradigmatic examples are myosin, a linear molecular motor, and F_1_- and V_1_-ATPases, rotary molecular motors (Suzuki, 2018; Kinoshita, 2021a). In this study, we deal primarily with F_1_- and V_1_-ATPases. F_1_-ATPase comprises three β subunits, three α subunits, and a γ subunit. The ATP hydrolysis cycle occurs in the β subunits, and the γ subunit rotates in the counterclockwise direction when F_1_-ATPase is viewed from the F_o_ side (Yoshidome et al., 2011; Kinoshita, 2021a). The B, A, and DF subunits in V_1_-ATPase correspond to the α, β, and γ subunits in F_1_-ATPase, respectively (Singharoy et al., 2019). That is, the ATP hydrolysis cycle occurs in the A subunits, and the DF subunit rotates in the counterclockwise direction when V_1_-ATPase is viewed from the V_o_ side. The central shaft, the γ subunit in F_1_-ATPase (α_3_β_3_γ complex) or the DF subunit in V_1_-ATPase (A_3_B_3_DF complex), performs unidirectional rotation.

Many experimental and theoretical efforts were devoted to the investigation of rotation mechanism of the rotary molecular motors, in particular, F_1_-ATPase. Typical examples of the theoretical studies are molecular dynamics (MD) simulations with coarse-grained (Koga and Takada, 2006; Kubo et al., 2020) or atomistic (Okazaki and Hummer, 2013; Czub and Grubmüller, 2014) models and analyses based on phenomenological or thermodynamic models (Nam and Karplus, 2019). These studies were conducted to explore specific subjects related to the mechanism or acquire microscopic information on the structure and properties of the α_3_β_3_γ complex. The elucidation of high efficiency suggested for the chemo-mechanical coupling (Toyabe et al., 2011; Toyabe and Muneyuki, 2015) has caught much attention. It was claimed that the chemo-mechanical coupling (transduction of chemical free energy of ATP to mechanical work) could be understood by a coarse-grained model (Vicatos et al., 2014) because the torque for rotating the γ subunit was generated only when an input of chemical free energy of ATP was incorporated, and the electrostatics played a dominant role (Mukherjee and Warshel, 2011; Mukherjee and Warshel, 2015). Despite the experimental and theoretical efforts mentioned above, the mechanism remains rather controversial. In our opinion, the problem in the previously reported studies is that the roles of water are unduly underestimated and a statistical-mechanical approach using a molecular model for water and treating the structure of the protein complex at the atomic level is missing.

According to the chemo-mechanical coupling, F_1_-ATPase transduces chemical free energy of ATP to mechanical work. F_1_-ATPase performs the mechanical work against the viscous resistance force by water for attaining the unidirectional rotation (Noji et al., 1997). In this case, F_1_-ATPase is regarded as the system and the aqueous solution in which F_1_-ATPase is immersed is regarded as the surroundings. The unidirectional movement of myosin along F-actin as well as the unidirectional rotation of the central shaft in F_1_-ATPase has been investigated extensively. Iwaki et al. (Iwaki et al., 2018) experimentally studied the effect of sucrose addition on the movement of myosin V. At a sucrose concentration of 1.25 mol/L, the viscosity of aqueous solution became ∼10 times higher but the movement velocity of the trailing head reduced only by a factor of ∼1/1.8: The viscous resistance force became ∼5.6 times stronger. Nevertheless, the head could move the same distance (∼77 nm) and perform ∼5.6 times more mechanical work. This result is not consistent with the chemo-mechanical coupling suggesting that the chemical free energy of ATP is transduced to mechanical work. By a novel experimental technique using high-speed atomic force microscopy (Kodera et al., 2010), for myosin V, Kodera et al. showed the following: The force responsible for the forward movement can be generated without transitioning through a state with ADP-Pi bound, meaning that no input of chemical free energy of ATP is required for the force generation; and even in the absence of ATP, when the trailing head is artificially detached from F-actin, it makes the forward movement (Kodera and Ando, 2014; Ngo et al., 2016). These observations conflict with the chemo-mechanical coupling.

Kinoshita and coworkers (Yoshidome et al., 2011; Yoshidome et al., 2012) have been developing a physical picture of rotation mechanism of F_1_-ATPase from a different point of view. Their approach is based on a statistical-mechanical theory wherein a molecular model is adopted for water, the structure of F_1_-ATPase is treated at the atomic level, and the effect of translational, configurational entropy of water in the system is highlighted. Recently, Kinoshita (Kinoshita, 2021a; Kinoshita, 2021b) constructed a more detailed physical picture where it is claimed that the system comprises not only F_1_-ATPase but also the aqueous solution in which F_1_-ATPase is immersed (i.e., the aqueous solution coexisting with F_1_-ATPase) and the system performs essentially no mechanical work during the rotation of the γ subunit. Strikingly, the torque driving the unidirectional rotation of the γ subunit is generated by water with no input of chemical free energy of ATP: A torque field acts on the γ subunit when it is accommodated within the α_3_β_3_ complex (such a torque field is absent for the γ subunit isolated). The mechanism of the torque generation becomes clearer as the argument proceeds; see Section 7.3.1 for the highlight. Though some of the basal concepts hypothesized in this physical picture need to be examined in further studies, such further examinations are difficult to achieve for F_1_-ATPase because crystal structures are experimentally available only for a limited number of different states. For V_1_-ATPase, on the other hand, crystal structures were experimentally solved by Murata and coworkers (Arai et al., 2013; Suzuki et al., 2016) for considerably more different states. Therefore, we have decided to perform a series of investigations based on statistical thermodynamics for V_1_-ATPase. Many of the results of theoretical analyses for V_1_-ATPase should be applicable to F_1_-ATPase as well. Our goal is to complete the physical pictures of rotation mechanisms of F_1_- and V_1_-ATPases the basal concepts of which are also applicable to the other ATP-driven proteins or protein complexes. This article provides an important first step in this direction.

Murata and coworkers (Arai et al., 2013; Singharoy et al., 2019; Arai et al., 2020) discussed how the unidirectional rotation of the DF subunit in V_1_-ATPase is induced by inferring the variations of chemical compounds bound to the three A subunits and the changes in structural properties of the three A-B subunit pairs during each ATP hydrolysis cycle. However, no physical rationalization was given from a statistical-thermodynamics viewpoint. In this study, we argue how the ATP hydrolysis cycle, variations of chemical compounds bound to the three A subunits, changes in the structural properties of the A_3_B_3_ complex, and unidirectional rotation of the DF subunit are correlated with one another using our statistical-thermodynamics analyses focused on the water-entropy effect.

As detailed in Supplementary Section S1, it was shown that many of the biological self-assembly processes in aqueous environment are driven by a gain of translational, configuration entropy of water. However, it is also essential to achieve and maintain the charge complementarity (see Section 2.2) in the structure formed. In F_1_- and V_1_-ATPases, the electrostatics should play important roles in the sense that the charge complementarity must not be broken during the rotation of the central shaft. Using all-atom MD simulations with explicit water for V_1_-ATPase, Singharoy and coworkers (Singharoy et al., 2017; Shekhar et al., 2022) showed that the electrostatic interactions at a specific residue-residue level and between a binding pocket and ATP, ADP+Pi, or ADP play important roles in the control of unidirectionality and rate of the rotation. The break of charge complementarity for a residue-residue pair must be compensated with the formation of charge complementarity for another residue-residue pair, and this is a significant factor in the rotation mechanism. In this study, we show that many of the fundamental characteristics of the rotation mechanisms of F_1_- and V_1_-ATPases can be elucidated by a model focused on the water-entropy effect and develop a theory based on a concept other than the chemo-mechanical coupling. Incorporation of the physical factors other than the water-entropy effect such as the electrostatics for making the theory more complete is a task for the future.

## 2 Crucial importance of translational, configurational entropy of water

### 2.1 Definitions of packing structure and packing efficiency related to translational, configurational entropy of water

**FIGURE 1:**
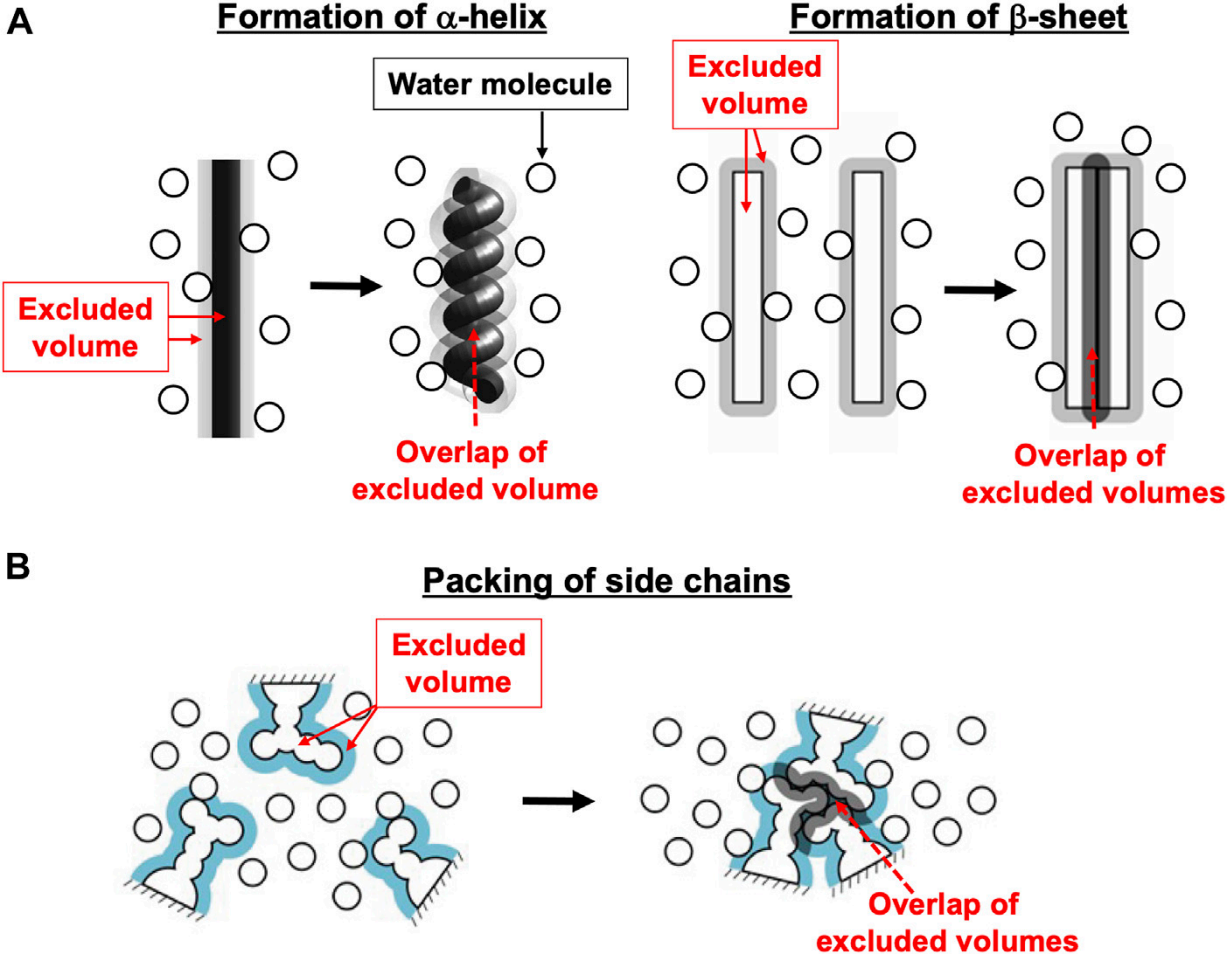
Basal processes in protein folding. **(A)** Formation of α-helix or β-sheet. **(B)** Close packing of side chains.

In the biological self-assembly, a gain of the translational, configurational entropy of water ascribed mainly to the mitigation of water crowding (i.e., entropic correlations among water molecules) in the system plays imperative roles (Kinoshita, 2013; Oshima and Kinoshita, 2015; Kinoshita, 2016) (Supplementary Section S1). The most fundamental self-assembly is protein folding (Figure 1). The presence of the backbone or a side chain of a protein generates an excluded volume (EV) to which the centers of water molecules are inaccessible (Harano and Kinoshita, 2005; Yoshidome et al., 2008; Yasuda et al., 2010; Hayashi et al., 2017; Hayashi et al., 2018). The formation of α-helix or β-sheet is accompanied by a reduction of the total EV generated by the backbone and an increase in the total volume available for the translational displacement of water molecules in the entire system, leading to a water-entropy gain. The formation undergoes an energy increase arising from the loss of protein-water hydrogen bonds. However, this energy increase is compensated with an energy decrease by the ensuring of protein intramolecular hydrogen bonds. Therefore, α-helix and β-sheet are very advantageous structural units from both entropic and energetic viewpoints (Hayashi et al., 2017; Hayashi et al., 2018). The close packing of side chains also leads to a reduction of the total EV followed by a water-entropy gain. The geometric properties of side chains play essential roles in the close packing. It was experimentally and theoretically shown that the water-entropy gain upon folding of apoplastocyanin (a protein with 99 residues) is as large as ∼670*k*_B_ (*k*_B_ is the Boltzmann constant) (Yoshidome et al., 2008). A protein folds so that its backbone and side chains can closely be packed with the formation of as much α-helix and β-sheet as possible (Hayashi et al., 2017; Hayashi et al., 2018). It is often, however, that the overall, sufficiently close packing cannot be achieved, in which case only the portions amenable to close packing are preferentially packed: In a protein, some portions are closely packed whereas the other portions are not. This nonuniform packing is more favorable than the overall uniform packing in terms of the water entropy (Yoshidome et al., 2011; Kinoshita, 2021a).

As rationalized in Supplementary Section S1, the translational, configurational entropy of water is a crucially important factor in the functional expression of F_1_-ATPase (Kinoshita, 2021a; Kinoshita, 2021b). Moreover, the nonuniform packing in F_1_-ATPase plays an essential role in its rotation mechanism. Hereafter, we use the terms, “packing efficiency (PE)” and “packing structure.” For a protein complex, from a viewpoint of the water entropy, it is required that not only the backbone and side chains of each protein in the complex but also the atoms in the protein interfaces be packed as closely as possible. However, it is often impossible to meet this requirement through uniform packing. The water entropy can become higher for nonuniform packing than for the uniform packing. The nonuniform packing means that only the portions of each protein and protein interfaces amenable to close packing are preferentially packed (Yoshidome et al., 2011; Kinoshita, 2021a). The PE signifies how close the packing is: When a protein in a complex or a protein interface is more closely packed, we state that its PE is higher. The physical meaning of the PE of a subunit or a subcomplex is made more definite in Section 4.1. The packing structure represents the differences in the PE among proteins and protein interfaces in a complex.

### 2.2 Electrostatics in aqueous environment

In determining which physical factors are more important than the others, it is absolutely necessary to employ an accurate model accounting for all the physical factors and compare their relative magnitudes quantitatively. By meeting this requirement for protein folding/denaturation problems (Inoue et al., 2020a; Inoue et al., 2020b) and protein-ligand binding processes (Yamada et al., 2019), we drew the conclusion that in aqueous environment the EV effect is a pivotal physical factor. Here, the EV effect works for increasing the translational, configurational entropy of water primarily by reducing the total EV. Matubayasi and coworkers (Kamo et al., 2016; Togunaga et al., 2018), using their all-atom MD simulation with explicit water based on a new solution theory in energy representation, showed that a useful modeling of protein folding is possible on the basis of not the electrostatics but the EV effect. (Graziano and coworkers (Merlino et al., 2017) also suggested that the EV effect is a principal driving force of protein folding.) This is because, for example, the decrease in protein intramolecular electrostatic interaction energy upon folding or transition to a more compact structure is accompanied by the penalty of electrostatic component of the energetic dehydration (i.e., the energy increase): The former is quite large but almost cancelled out by the latter that is equally large (Kinoshita, 2016; Kinoshita, 2021a), which can never be reproduced by a dielectric continuum model for water. In general, for a solute in aqueous solution, the EV effect becomes stronger as its size increases.

The intramolecular and intermolecular electrostatic attractive interactions of biomolecules do not work as a driving force of the biological self-assembly. However, the electrostatics is important in the following respect. When charged groups are buried upon protein folding or protein-ligand binding, the contact of oppositely charged groups (i.e., gain of electrostatic attractive interaction between groups with positive and negative charges) are essential for compensating for the electrostatic energetic dehydration penalty, namely, the loss of electrostatic attractive interactions between the group with a positive charge and water oxygens and between the group with a negative charge and water hydrogens. (Water oxygens and hydrogens carry negative and positive partial charges, respectively.) Thus, it is important to insure the charge complementarity in the biological self-assembly. The charge complementarity achieved in the stabilization of the structure of F_1_- or V_1_-ATPase is to be maintained during the rotation of the central shaft.

## 3 Features of the physical picture of rotation mechanism of F_1_-ATPase recently developed by Kinoshita

### 3.1 Involvement of an ATP-driven protein or protein complex in ATP hydrolysis cycle

The ATP hydrolysis reaction is extremely slow in bulk aqueous solution in the absence of a catalyst. Since an ATP-driven protein or protein complex works as the catalyst for accelerating the reaction, it is involved in the reaction through the ATP hydrolysis cycle composed of binding of ATP to it, hydrolysis of ATP into ADP and Pi in it, and dissociation of ADP and Pi from it (Kinoshita, 2021a).

In F_1_-ATPase, a β subunit works as the catalyst. Let *C*_*X*_ (mol/L) be the concentration of *X*. Unless otherwise specified, we consider the following aqueous-solution condition: *C*_ATP_ is sufficiently higher than *C*_ADP_ and *C*_Pi_ such that the reaction of ATP+H_2_O→ADP+Pi (ATP hydrolysis) occurs much more frequently than that of ATP+H_2_O←ADP+Pi (ATP synthesis) and the overall reaction is unidirectional and not ATP synthesis but ATP hydrolysis (Supplementary Section S3); and *C*_ATP_ is sufficiently high and *C*_ADP_ and *C*_Pi_ are sufficiently low such that on the whole, binding of ATP to and dissociation of ADP and Pi from a β subunit take place (Supplementary Section S4). It follows that the ATP hydrolysis cycle occurs spontaneously for lowering the system free energy.

### 3.2 Features of the physical picture

Kinoshita’s physical picture (Kinoshita, 2021a; Kinoshita, 2021b) is distinguished from the others in the following respects.

(1) We must consider that the system comprises not only F_1_-ATPase but also the aqueous solution of ATP, ADP, and Pi in which F_1_-ATPase is immersed when we refer to the decrease in system free energy upon a state change of the system. The system exchanges energy with the surroundings (i.e., the heat bath) through heat and work. For the system comprising F_1_-ATPase and the aqueous solution such as the one employed in a single-molecule experiment, the pressure-volume work performed by the system against the pressure of the surroundings *P*, *P*Δ*V* (Δ*V* is the change in system volume), is the only mechanical work. Since *P*Δ*V* is negligibly small, the system performs essentially no mechanical work (for Δ*V*<0, the work is performed by the pressure of the surroundings). This argument is based on the fact that a structural change of a protein (Yoshidome et al., 2008; Inoue et al., 2020a) or protein-ligand binding (Yamada et al., 2019) is accompanied by only small Δ*V* and *P*|Δ*V*|<<*k*_B_ *T* (the absolute temperature *T* is set at 298 K in this article) at *P* = 1 atm (Supplementary Section S1.1).
(2) It is suggested that the γ subunit is rotated by an entropic torque generated by water. The translational, configurational entropy of water is justifiably a pivotal component of the system free energy. A packing structure of F_1_-ATPase is stabilized so that this component can be almost maximized. During the ATP hydrolysis cycle which spontaneously occurs, however, the packing structure of the α_3_β_3_ complex is sequentially changed. The orientation of the γ subunit featuring its asymmetrical structure is also changed in accordance with the packing structure of the α_3_β_3_ complex for avoiding a water-entropy loss (the water entropy is strongly dependent on the orientation). The rotation of the γ subunit is thus induced.

## 4 Analyses on F_1_-ATPase already performed and those to be performed further

### 4.1 Measure of packing efficiency of a subunit or subcomplex

The PE and packing structure related to the water entropy can be analyzed only by a statistical-mechanical approach using a molecular model for water and accounting for the complex structure at the atomic level. The measure of the PE of a subunit or subcomplex with nothing bound, *η*, is defined as

$$\eta =“S_{1}\;of\;a\;subunit\;or\;subcomplex”$$ (1a)

Here, *S*_1_ is the loss of water entropy (its major component is the translational, configurational entropy) upon the creation of a cavity, which geometrically matches the polyatomic structure of the subunit or subcomplex, in a fixed position within water, and *S*_1_∼*S* where *S* is the hydration entropy (Supplementary Section S1.1) (Hikiri et al., 2019). Smaller | *η* | implies higher PE. The measure of the PE of a subunit or subcomplex with chemical compound *Y* bound, *η*_*Y*_, is defined as

$$\eta_{Y}=“S_{1}\;of\;a\;subunit\;or\;subcomplex\;with\;Y\;bound”-“S_{1}\;of\;Y”$$ (1b)

Smaller | *η*_*Y*_ | implies higher PE. For the comparison between a subunit or subcomplex with nothing bound and that with *Y* bound in terms of the PE, Eqs 1a, 1b can be replaced by Eqs 2a, 2b, respectively:

$$\eta ’=“S_{1}\;of\;a\;subunit\;or\;subcomplex”+“S_{1}\;of\;Y”$$ (2a)

$$\eta {’}_{Y}=“S_{1}\;of\;a\;subunit\;or\;subcomplex\;with\;Y\;bound”$$ (2b)

It is unequivocal that the following equation holds:

$$\eta -\eta_{Y}=\eta ’-\eta {’}_{Y}$$ (3)

### 4.2 Nucleotide bindings to two of β subunits

**FIGURE 2:**
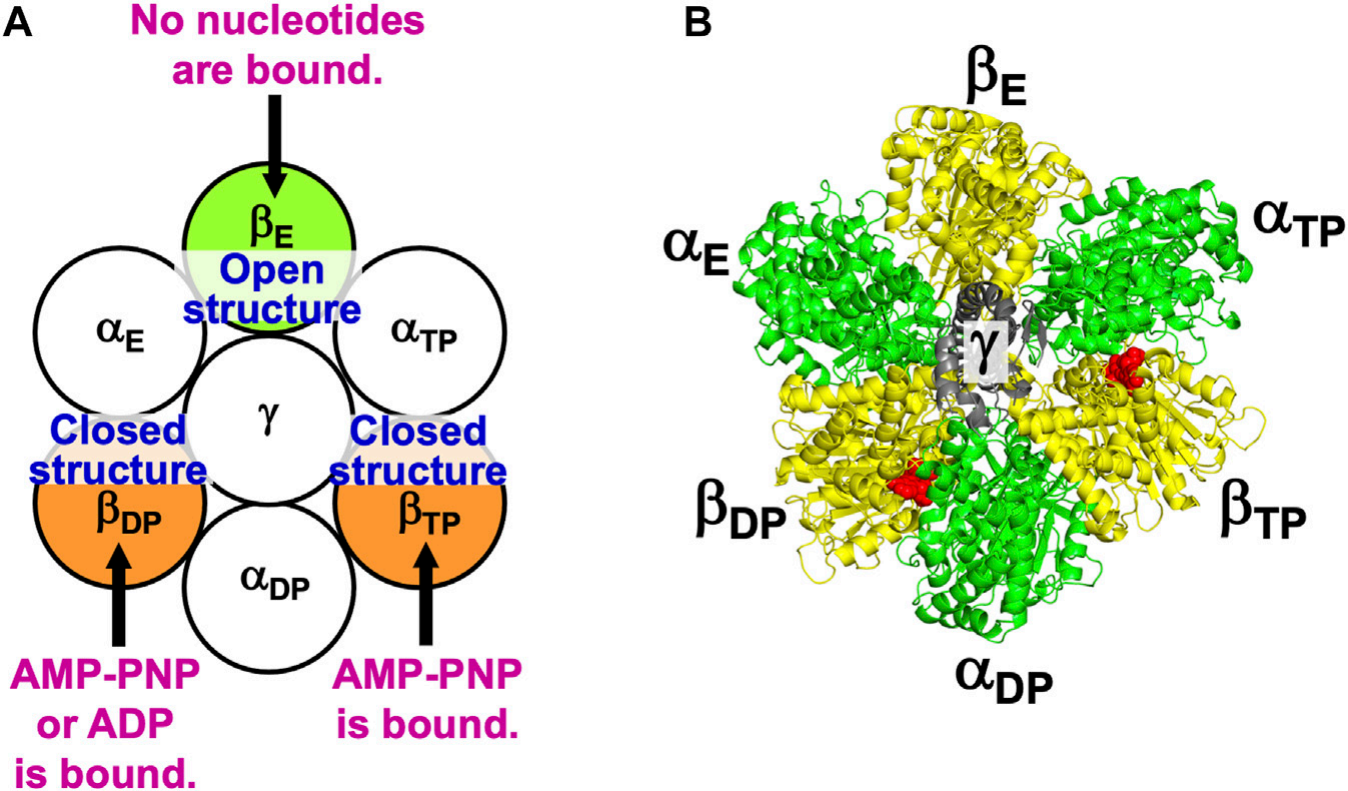
**(A)** State of F_1_-ATPase, α_3_β_3_γ complex, in which two nucleotides are bound to two of the three β subunits, respectively. “Nucleotides” signifies ATP, ADP, and related chemical compounds. Following the works of Walker and coworkers (Bowler et al., 2007), the three β subunits are named β_DP_, β_TP_, and β_E_, respectively, and the three α subunits are named α_DP_, α_TP_, and α_E_, respectively. In aqueous solution of AMP-PNP and ADP, the nucleotides bound to β_DP_ and β_TP_ are AMP-PNP and AMP-PNP, respectively. In aqueous solution of AMP-PNP, ADP, and azide, the nucleotides bound to β_DP_ and β_TP_ are ADP and AMP-PNP, respectively. Azide stabilizes the β subunit with ADP bound (Bowler et al., 2007). The structure of a β subunit with a nucleotide bound is closed whereas that without a nucleotide bound is open. **(B)** Top view of ribbon representation of α_3_β_3_γ-complex structure stabilized in aqueous solution of AMP-PNP and ADP (the Protein Data Bank (PDB) Code is 2JDI) (Bowler et al., 2007). The β subunits, α subunits, and γ subunit are colored yellow, green, and gray, respectively. AMP-PNP is represented by the red fused spheres. (AMP-PNP bound to each of the three α subunits is not shown.)

Figure 2 illustrates the state stabilized in aqueous solution of nucleotides, which was experimentally observed (Bowler et al., 2007). Two nucleotides are bound to two of the three β subunits, respectively. AMP-PNP is an analogue of ATP and not hydrolyzed. AMP-PNP, which can thus be regarded as ATP whose hydrolysis does not occur, is used when the solution of a crystal structure is undertaken. States where the number of nucleotides bound to the three β subunits is zero, one, and three should be less stable (Supplementary Section S5), which is also the case for V_1_-ATPase. (Hereafter, unless otherwise mentioned, ATP and AMP-PNP are not distinguished.) It was observed that β_DP_, β_TP_, and β_E_ take closed, closed, and open structures, respectively (Bowler et al., 2007). Namely, the backbone and side chains in a β subunit with a nucleotide bound is more closely packed than those in a β subunit with no nucleotides bound. Let β(*Y*) denote the PE of a β subunit to which chemical compound *Y* is bound and β denote the PE of a β subunit with nothing bound. It is experimentally definite that the following inequalities hold:

$$\beta \left(ATP\right)>\beta ;\;\beta \left(ADP\right)>\beta$$ (4)

The state illustrated in Figure 2A is stabilized so that the water entropy can be almost maximized.

### 4.3 Structural rotation of α_3_β_3_ complex without central shaft

In aqueous solution of ATP, ADP, and Pi, even without the γ subunit, the structural rotation in the counterclockwise direction, “β[open]-β[closed]-β[closed] shown in Figure 2A” → “β[closed]-β[open]-β[closed] after 120° rotation” → “β[closed]-β[closed]-β[open] after 240° rotation” → ⋅⋅⋅, occurs in the α_3_β_3_ complex (Uchihashi et al., 2011). Here, β[open], for instance, denotes a β subunit possessing open structure. This experimental result indicates that the rotation mechanism is programmed in the α_3_β_3_ complex.

### 4.4 Definitions of subcomplexes 1, 2, and 3

**FIGURE 3:**
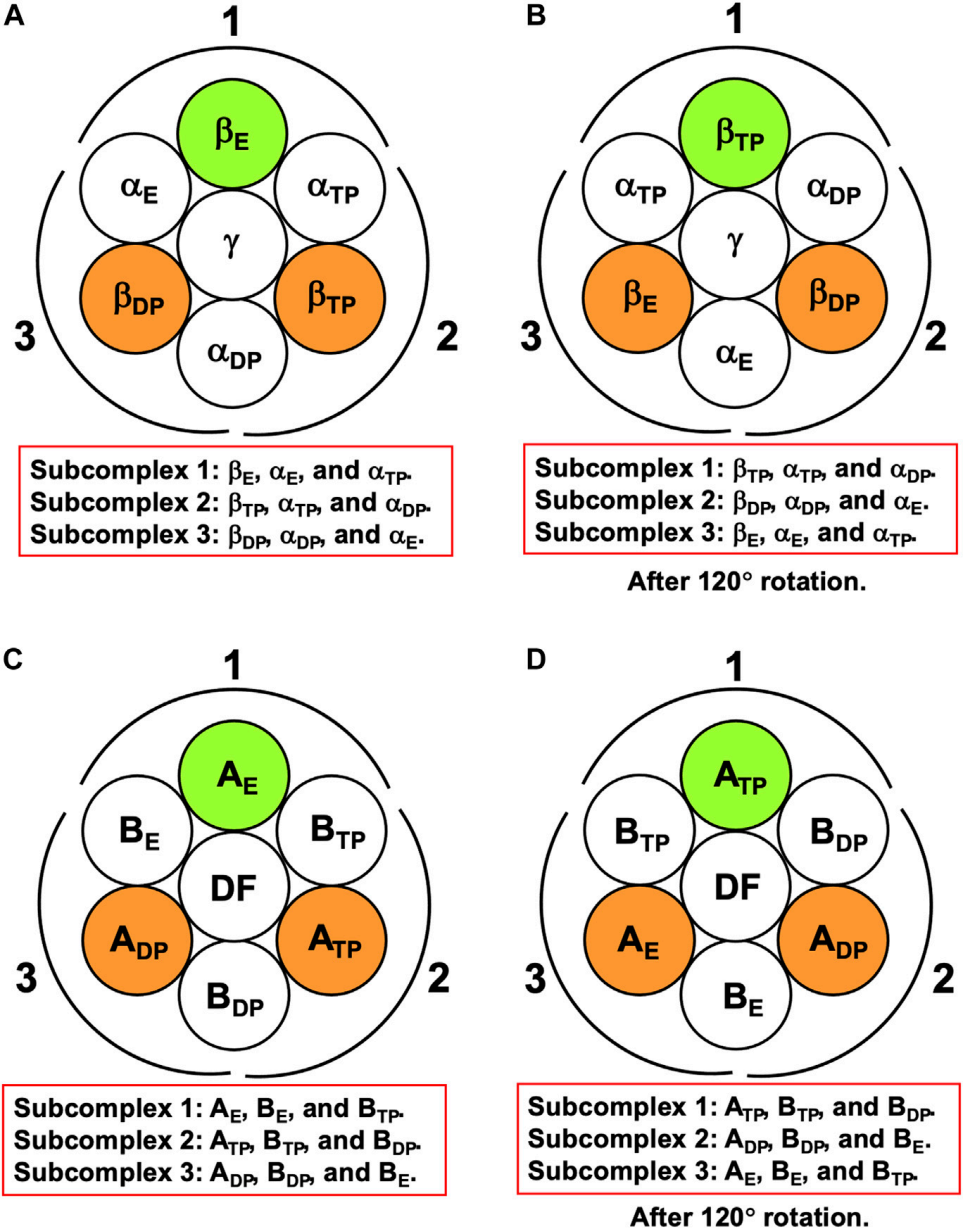
**(A)** Definition of subcomplexes 1, 2, and 3 for F_1_-ATPase. The three subcomplexes are named in respect of their positions. Two nucleotides are bound to β_DP_ and β_TP_, respectively. **(B)** Subcomplexes 1, 2, and 3 after 120° rotation of γ subunit. **(C)** Definition of subcomplexes 1, 2, and 3 for V_1_-ATPase. The three subcomplexes are named in respect of their positions. Two nucleotides are bound to A_DP_ and A_TP_, respectively. **(D)** Subcomplexes 1, 2, and 3 after 120° rotation of DF subunit.

For F_1_-ATPase, we then define three subcomplexes regardless of the presence of the γ subunit as follows (Figure 3A):

$$Subcomplex\;1:\beta_{E},\alpha_{E},and\;\alpha_{TP},$$

$$Subcomplex\;2:\beta_{TP},\alpha_{TP},and\;\alpha_{DP},$$

$$Subcomplex\;3:\beta_{DP},\alpha_{DP},and\;\alpha_{E}.$$

The three subcomplexes are named in respect of their positions. Hence, after the γ subunit rotates by 120° (Figure 3B), subcomplex 3, for instance, now comprises β_E_, α_E_, and α_TP_. β_DP_, β_TP_, and β_E_ take closed, closed, and open structures, respectively: The backbone and side chains are more closely packed in β_DP_ or β_TP_ than in β_E_.

It is the best to define the three subcomplexes for V_1_-ATPase here. They are defined as follows regardless of the presence of the DF subunit (Figure 3C):

$$Subcomplex\;1:A_{E},B_{E},and\;B_{TP},$$

$$Subcomplex\;2:A_{TP},B_{TP},and\;B_{DP},$$

$$Subcomplex\;3:A_{DP},B_{DP},and\;B_{E}.$$

**FIGURE 4:**
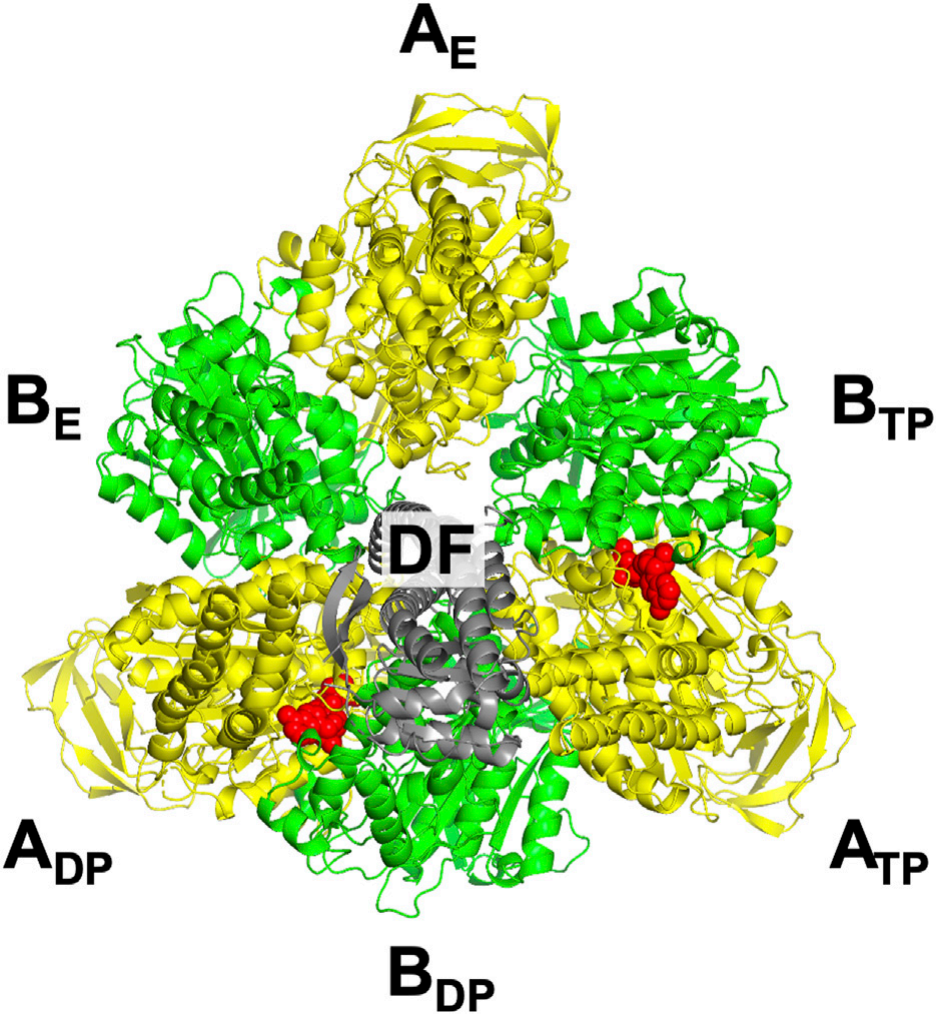
Top view of ribbon representation of A_3_B_3_DF-complex structure stabilized in aqueous solution of AMP-PNP and ADP. The A subunits, B subunits, and DF subunit are colored yellow, green, and gray, respectively. AMP-PNP is represented by the red fused spheres. (Unlike in the case of F_1_-ATPase, AMP-PNP bindings to the three B subunits do not occur.)

The three subcomplexes are named in respect of their positions. Hence, after the DF subunit rotates by 120° (Figure 3D), subcomplex 3, for instance, now comprises A_E_, B_E_, and B_TP_. In this study, V_1_-ATPase is viewed from the V_o_ side and the central shaft, the DF subunit, rotates in the counterclockwise direction. The structure of the A_3_B_3_DF complex stabilized in aqueous solution of AMP-PNP and ADP is depicted in Figure 4. The naming for the six subunits of V_1_-ATPase adopted is the same as that for the six subunits of F_1_-ATPase. Hence, a detailed comparison between these two rotary molecular motors can be made. (In Supplementary Table S1, we summarize the differences between previous studies by Murata and coworkers (Arai et al., 2013; Suzuki et al., 2016) and this study in naming the A-B subunit pairs.) The subscript “DP” or “TP” for “A” in the naming does not necessarily imply that ADP or ATP is bound to the A subunit.

### 4.5 Basal concepts hypothesized in the physical picture for F_1_-ATPase

Kinoshita’s physical picture was constructed on the basis of observations in single-molecule experiments by Noji and coworkers (Yasuda et al., 1998; Yasuda et al., 2001; Adachi et al., 2007; Watanabe et al., 2010), those by Yoshida and coworkers (Shimabukuro et al., 2003), and theoretical analyses performed by Kinoshita and coworkers (Yoshidome et al., 2011; Yoshidome et al., 2012). The basal concepts hypothesized in this physical picture can be summarized as follows.

(1) Irrespective of the presence of nucleotides bound and the incorporation of the γ subunit, the PE of each subcomplex is determined by that of the β subunit in it. For instance, when the PE for a β subunit follows the order, “β subunit in subcomplex 3”>“β subunit in subcomplex 2”>“β subunit in subcomplex 1”, the PE for a subcomplex follows the order, “subcomplex 3”>“subcomplex 2”>“subcomplex 1”.
(2) The PE of a β subunit is variable depending on the chemical compound bound to it: ATP, ATP(ATP-H_2_O), ATP-H_2_O, ADP+Pi, or Pi. Here, ATP-H_2_O denotes ATP just before the ATP hydrolysis reaction and ATP(ATP-H_2_O) is an intermediate between ATP and ATP-H_2_O. (ATP(ATP-H_2_O) and ATP-H_2_O are the activated complexes.) The following inequality is justified as an extension of Order (4):

$$\beta \left(Nucleotide\right)>\beta$$ (5)

where “Nucleotide” signifies ATP, ATP(ATP-H_2_O), ATP-H_2_O, ADP+Pi, or ADP. We showed that β>β(Pi) in Order (6) holds as explained in what follows. Crystal structures of yeast F_1_-ATPase were solved for the following two states: a state which can be regarded as the catalytic dwell state where Pi was bound to β_E_ and AMP-PNPs were bound to β_DP_ and β_TP_, respectively; and the state after 16° rotation of the γ subunit (the first one is the state before the 16° rotation) (Kabaleeswaran et al., 2006). Since the ATP hydrolysis does not occur in β_DP_, the rotation is triggered by the dissociation of Pi from β_E_. We analyzed the packing structures of the two states by statistical-thermodynamics theory (Yoshidome et al., 2012) and all-atom MD simulation (Ito et al., 2013), and a significant result obtained was β>β(Pi). This result and the discussion in Section 7.1.2 lead to the following inequalities:

$$\beta >\beta \left(Pi\right);\;\beta \left(Nucleotide\right)>\beta \left(Pi\right);\;\beta \left(ATP‐H_{2}O\right)>\beta \left(ADP+Pi\right)>\beta \left(Pi\right)$$ (6)

In the construction of the physical picture detailed in Section 7.1.2, it was assumed that the following inequality holds:

$$\begin{array}{c} \beta \left(ATP‐H_{2}O\right)>\beta \left(ATP\left(ATP‐H_{2}O\right)\right)>\beta \left(ATP\right);\;\beta \left(ATP‐H_{2}O\right)>\beta \left(ADP+Pi\right)>\beta \left(Pi\right);\\ \beta \left(ATP\right)>\beta >\beta \left(Pi\right). \end{array}$$ (7)

Comparing Order (7) with Orders (5) and (6), we notice that the inequality, β(ATP-H_2_O)>β(ATP(ATP-H_2_O))>β(ATP), is to be corroborated in further studies.

(3) The chemical compound bound to each β subunit is sequentially changed by the ATP binding to it, progress of ATP hydrolysis into ADP and Pi in it, and dissociation of ADP and Pi from it. As a result, the PEs of each β subunit and the subcomplex in which it is incorporated are also sequentially changed, which leads to sequential changes in packing structure of the α_3_β_3_ complex.
(4) The orientation of the γ subunit, on which the water entropy is strongly dependent, is changed in response to the change in packing structure of the α_3_β_3_ complex. That is, the sequential change in packing structure of the α_3_β_3_ complex induces the rotation of the γ subunit for retaining the water entropy.
(5) During each ATP hydrolysis cycle, the stabilized state of F_1_-ATPase, the state where two nucleotides are bound to two of the three β subunits, respectively, is maintained. In each cycle, one ATP molecule is hydrolyzed in bulk aqueous solution.

Some of the basal concepts described above are to be examined in further studies, and they are outlined in Table 1 (the others have already been justified). For F_1_-ATPase, however, crystal structures are experimentally known only for the α_3_β_3_γ complexes with no nucleotides bound and with two AMP-PNPs bound (Kinoshita, 2021a). A crystal structure of the α_3_β_3_γ complex with one AMP-PNP and one ADP bound was also solved, but the ADP binding was made by a bound azide ion (Bowler et al., 2007). There is not a crystal structure where ADP is bound to a β subunit without the azide ion. Hence, the difference in the PE between two β subunits to which ATP or AMP-PNP and ADP are bound, respectively, cannot be examined. Since crystal structures without the γ subunit (i.e., the α_3_β_3_ complex) are not found in the literature, effects due to the incorporation of the γ subunit cannot be analyzed. These problems can be solved by treating V_1_-ATPase instead.

**TABLE 1:** Basal concepts to be examined in further studies for physical picture of rotation mechanism of F_1_-ATPase. β(*Y*) denotes the packing efficiency (PE) of a β subunit in F_1_-ATPase to which chemical compound *Y* is bound. “Nucleotides” signifies ATP, ADP, and related chemical compounds. ATP-H_2_O denotes ATP just before the ATP hydrolysis reaction, and ATP(ATP-H_2_O) is an intermediate between ATP and ATP-H_2_O (ATP(ATP-H_2_O) and ATP-H_2_O are the activated complexes).

Basal concepts hypothesized in physical picture	Issues to be pursued in further studies
The PE of each subcomplex is determined by that of the β subunit in it	The concept mentioned in the left column was corroborated only for the α_3_β_3_γ complex with two ATPs bound (Kinoshita, 2021a)
This is true irrespective of the presence of nucleotides bound and the incorporation of the γ subunit	It is to be corroborated for the α_3_β_3_γ complex with no nucleotides bound, α_3_β_3_ complex with two ATPs bound, and α_3_β_3_ complex with no nucleotides bound
The PEs follow Order (7)	Examination of the order, β(ATP-H_2_O)>β(ATP(ATP-H_2_O))>β(ATP)
Bindings of ATP and ADP have large effects on the packing structure of F_1_-ATPase	Quantitative evaluation of the effects mentioned in the left column

## 5 Materials and methods

### 5.1 Crystal structures of V_1_-ATPase

Crystal structures were solved for the A_3_B_3_ complexes with no nucleotides bound (the PDB Code is 3VR2 and the resolution *R* is 2.8 Å) and two AMP-PNPs bound (3VR3 and *R* = 3.4 Å) and for the A_3_B_3_DF complexes with no nucleotides bound (3VR4 and *R* = 2.2 Å) and two AMP-PNPs bound (3VR6 and *R* = 2.7 Å) (Arai et al., 2013). A crystal structure is also available for the A_3_B_3_DF complex with two ADPs bound (5KNB and *R* = 3.3 Å): It is a state stabilized in aqueous solution of ADP in the absence of azide ions (Suzuki et al., 2016). Unlike for F_1_-ATPase, we can analyze not only the separate effects due to the binding of nucleotides and the incorporation of the DF subunit in the A_3_B_3_ complex but also the difference between two A subunits to which ATP and ADP are bound, respectively, in the PE. See Supplementary Section S6 for more information.

In this study, the hydration entropy is calculated by adopting fixed structures. We believe, however, that the basal aspects of our conclusions are not likely to be altered by accounting for the structural fluctuation.

### 5.2 Calculation of hydration entropy of a solute

For calculating the hydration entropy *S*_1_ (*S*_1_∼*S*: *S*_1_ can be referred to as the hydration entropy) of a solute (i.e., a chemical compound, protein, or protein complex) with a fixed structure, we employ our hybrid (Hikiri et al., 2019) of the angle-dependent integral equation theory (ADIET) combined with a multipolar model for water (Kusalik and Patey, 1988a; Kusalik and Patey, 1988b; Kinoshita, 2008) and the morphometric approach (MA) (Roth et al., 2006; Oshima and Kinoshita, 2015; Hayashi et al., 2018), the ADIET-MA method. Since the ADIET is employed, not only the translational contribution but also the orientational one is included in *S*_1_ though the latter is much smaller (Supplementary Section S1.1). More detailed descriptions of the ADIET and the MA are given in Supplementary Section S7. The high accuracy of the ADIET-MA method was corroborated in our earlier works (Hikiri et al., 2019; Yamada et al., 2019).

### 5.3 Analysis on packing efficiencies of A subunits and subcomplexes 1, 2, and 3

We analyze the PEs of A_DP_, A_TP_, and A_E_ and those of the three subcomplexes. The key quantity is *S*_1_, and smaller | *S*_1_ | implies that the protein or subcomplex is more closely packed and its PE is higher. We use the crystal structures of the A_3_B_3_ complexes with no nucleotides bound and two AMP-PNPs bound (A_3_B_3_⋅2AMP-PNP) and those of the A_3_B_3_DF complexes with no nucleotides bound, two AMP-PNPs bound (A_3_B_3_DF⋅2AMP-PNP), and two ADPs bound (A_3_B_3_DF⋅2ADP). In A_3_B_3_⋅2AMP-PNP and A_3_B_3_DF⋅2AMP-PNP, AMP-PNP and Mg^2+^ are bound to A_DP_ and A_TP_ but nothing is bound to A_E_. In the calculation of *S*_1_, AMP-PNP is replaced by ATP. Since A_E_ has fewer atoms than the other two A subunits, *S*_1_ of ATP-Mg^2+^ is added to *S*_1_ of any protein, protein pair, or protein complex including A_E_ for impartial comparison among their PEs (i.e., Eqs 2a, 2b are adopted). A similar treatment is made for A_3_B_3_DF⋅2ADP. For all the crystal structures, the slight overlaps of the protein atoms are removed using a standard energy-minimization technique (Yoshidome et al., 2011).

### 5.4 Analysis on packing efficiency of interface between two subunits

The water-entropy gain upon contact of subunits *I* and *J*, *ΔS*_*IJ*_, is given by

$$\Delta S_{IJ}=“S_{1}\;of\;subunit\;pair\;I-J”-\left(“S_{1}\;of\;subunit\;I”+“S_{1}\;of\;subunit\;J”\right)$$ (8)

Subunit pair *I*-*J* is taken from a complex, and subunits *I* and *J* are obtained by simply separating the pair. A-B, A-DF, and B-DF are considered as the subunit pairs. Larger *ΔS*_*IJ*_ represents that the interface between subunits *I* and *J* are more closely packed, i.e., the PE of the interface is higher. For the interface between an A subunit with ATP bound and a B subunit, *ΔS*_*IJ*_ is calculated for A**⋅**ATP+B→A**⋅**ATP-B (*I* = A**⋅**ATP and *J* = B where A**⋅**ATP is an A subunit with ATP bound).

### 5.5 Analysis on effect of ATP or ADP on affinity between A and B subunits

We calculate the water-entropy gain Δ*S*_1_ (∼Δ*S*) upon association of A_DP_ and B_DP_ or A_TP_ and B_TP_ for the three cases where nothing, ATP, and ADP bind to A_DP_ or A_TP_, respectively. Δ*S*_1_ upon A_DP_+ATP+B_DP_→A_DP_ **⋅**ATP-B_DP_, for example, is “*S*_1_ of A_DP_ **⋅**ATP-B_DP_”−(“*S*_1_ of A_DP_”+“*S*_1_ of ATP”+“*S*_1_ of B_DP_”). A_DP_, A_TP_, and B_DP_ are obtained by simply separating A_DP_ **⋅**ATP-B_DP_ in the calculation. Larger Δ*S*_1_ implies higher affinity between the A and B subunits and closer packing of their interface.

## 6 Results

### 6.1 Packing structure of A_3_B_3_DF complex with two ATPs (AMP-PNPs) bound, A_3_B_3_DF⋅2ATP

**FIGURE 5:**
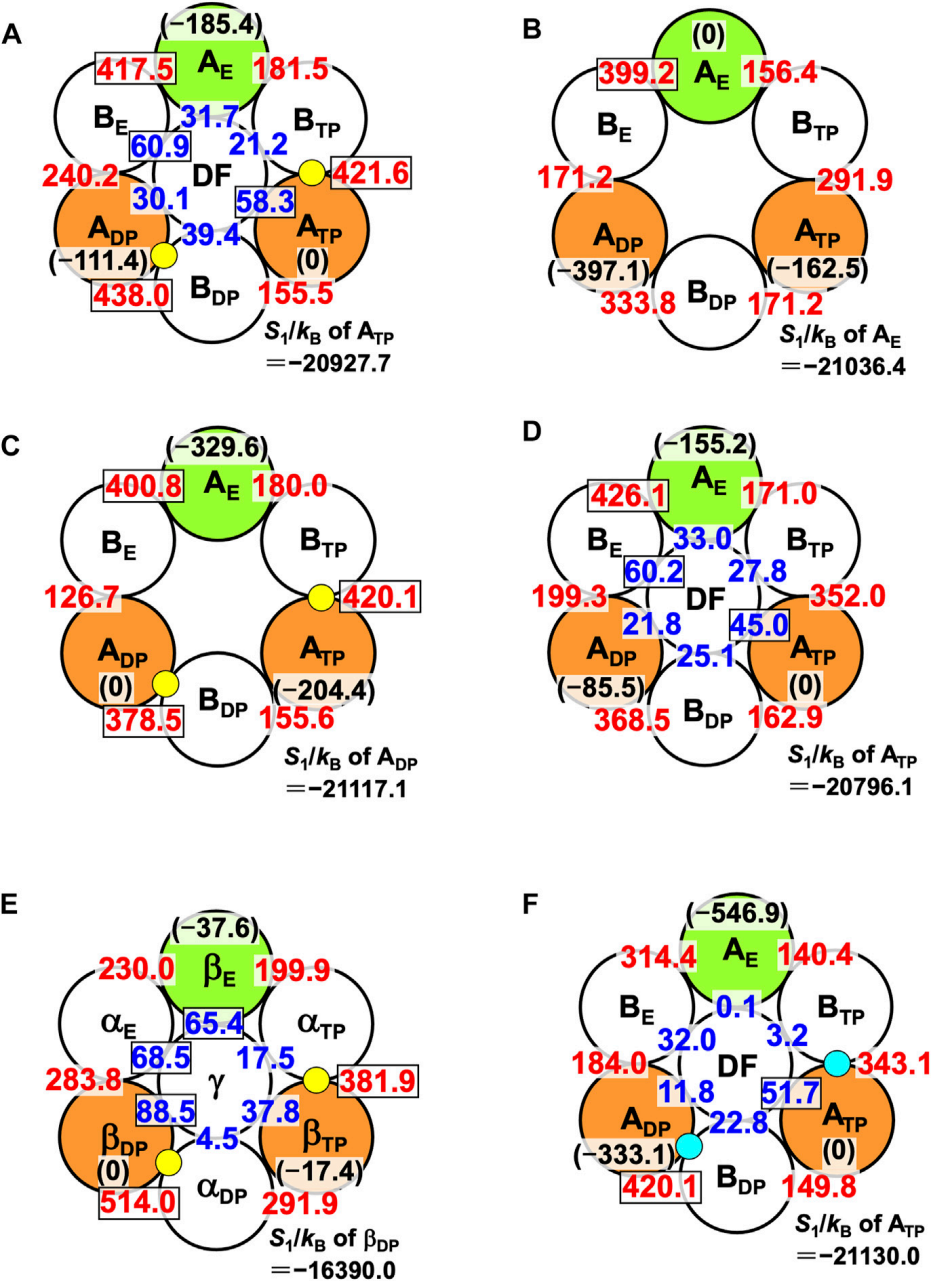
Packing structures of **(A)** A_3_B_3_DF⋅2ATP, **(B)** A_3_B_3_, **(C)** A_3_B_3_⋅2ATP, and **(D)** A_3_B_3_DF. A yellow circle denotes ATP. A number colored red quantifies *ΔS*_*IJ*_/*k*_B_ (subunit *I* is an A subunit and subunit *J* is a B subunit; see Eq. 8). For example, *ΔS*_*IJ*_/*k*_B_ in **(A)** for which subunits *I* and *J* denote A_DP_ and B_DP_, respectively, is 438.0. A number colored blue quantifies *ΔS*_*IJ*_/*k*_B_ (subunit *I* is an A subunit or a B subunit and subunit *J* is the DF subunit). For example, *ΔS*_*IJ*_/*k*_B_ in **(A)** for which subunits *I* and *J* denote B_E_ and the DF subunit, respectively, is 60.9. Larger *ΔS*_*IJ*_/*k*_B_ implies closer packing of the interface of subunit pair *I*-*J*. In **(A)**, for example, a number colored in black within parentheses denotes *S*_1_/*k*_B_ (*S*_1_/*k*_B_<0) of an A subunit relative to *S*_1_/*k*_B_ of A_TP_. For example, “*S*_1_/*k*_B_ of A_E_”−“*S*_1_/*k*_B_ of A_TP_” is −185.4. Smaller | *S*_1_/*k*_B_ | implies closer packing or higher PE of an A subunit. The PE follows the order, A_TP_>A_DP_>A_E_. Relatively large numbers are surrounded by rectangles. (In (B), a number colored in black within parentheses denotes *S*_1_/*k*_B_ (*S*_1_/*k*_B_<0) of an A subunit relative to *S*_1_/*k*_B_ of A_E_. For example, “*S*_1_/*k*_B_ of A_TP_”−“*S*_1_/*k*_B_ of A_E_” is −162.5.) *S*_1_/*k*_B_ of the A subunit which is the most closely packed among the three A subunits is also given. **(E)** Packing structures of catalytic dwell states for F_1_-ATPase (α_3_β_3_γ complex with two ATPs bound). *S*_1_/*k*_B_ of the A or β subunit which is the most closely packed among the three A or β subunits is also given. **(F)** Packing structure of A_3_B_3_DF⋅2ADP. A right-blue circle denotes ADP. *S*_1_/*k*_B_ of the A subunit which is the most closely packed among the three A subunits is also given.

The packing structure of A_3_B_3_DF**⋅**2ATP, one of the most stable states, is illustrated in Figure 5A. The hydration entropies of subcomplexes 1, 2, and 3 in this complex, which provide information on their PEs, are given in Table 2. (See Supplementary Section S9 emphasizing that the calculation results are reliable.) We find the following:

“PE of subcomplex 2”>“PE of subcomplex 3”>“PE of subcomplex 1” (9)

$$“PE\;of\;A_{TP}”>“PE\;of\;A_{DP}”>“PE\;of\;A_{E}”$$ (10)

**TABLE 2:** Hydration entropies of subcomplexes 1, 2, and 3 in (A) A_3_B_3_DF⋅2ATP, (B) A_3_B_3_, (C) A_3_B_3_⋅2ATP, (D) A_3_B_3_DF, and (F) A_3_B_3_DF⋅2ADP. Subcomplexes 1, 2, and 3 are defined in Figure 7A. “1 in (A)”, for example, denotes subcomplex 1 in the A_3_B_3_DF complex with two ATPs bound (A_3_B_3_DF⋅2ATP). The “relative value of *S*_1_/*k*_B_” signifies *S*_1_/*k*_B_ (*S*_1_/*k*_B_<0) of a subcomplex relative to *S*_1_/*k*_B_ of the subcomplex with the smallest value of | *S*_1_/*k*_B_ |. For example, “*S*_1_/*k*_B_ of subcomplex 2 in (B)”−“*S*_1_/*k*_B_ of subcomplex 1 in (B)” = −524.4. Smaller | *S*_1_/*k*_B_ | implies closer packing or higher packing efficiency (PE) of the subcomplex. In (B), for example, the PE follows the order, subcomplex 1> subcomplex 2> subcomplex 3.

Subcomplex	Relative value of *S*_1_/*k*_B_
1 in (A)	(−295.5)
2 in (A)	(0)
3 in (A)	(−189.2)
1 in (B)	(0)
2 in (B)	(−524.4)
3 in (B)	(−718.4)
1 in (C)	(−255.5)
2 in (C)	(−57.5)
3 in (C)	(0)
1 in (D)	(−61.2)
2 in (D)	(0)
3 in (D)	(−18.2)
1 in (F)	(−594.7)
2 in (F)	(0)
3 in (F)	(−457.0)

Thus, the PE of each subcomplex is determined by that of the A subunit in it, as in the case of α_3_β_3_γ**⋅**2ATP. Among the six A-B interfaces, A_DP_-B_DP_, A_TP_-B_TP_, and A_E_-B_E_ are relatively more closely packed (A_DP_-B_DP_ is the most closely packed). Among the six A-DF and B-DF interfaces, B_E_-DF and A_TP_-DF are relatively more closely packed (B_E_-DF is the most closely packed).

The packing structure of V_1_-ATPase (A_3_B_3_DF**⋅**2ATP) (Figure 5A) can be compared with that of F_1_-ATPase (α_3_β_3_γ**⋅**2ATP) (Figure 5E). The packing structure of F_1_-ATPase was unveiled in Ref. (Yoshidome et al., 2011). In F_1_-ATPase, “PE of subcomplex 3”>“PE of subcomplex 2”>“PE of subcomplex 1” and “PE of β_DP_”>“PE of β_TP_”>“PE of β_E_”.

We now discuss the values of *ΔS*_*IJ*_/*k*_B_ for the DF-A and DF-B interfaces. *ΔS*_*IJ*_/*k*_B_ is largest (60.9) for the DF-B_E_ interface and smallest (21.2) for the DF-B_TP_ interface (Figure 5A), and the difference between these two values, ∼40, is significantly large. The orientation of the DF subunit is determined so that the sum of the six values of *ΔS*_*IJ*_/*k*_B_ can be maximized. The sum is ∼242. If the DF subunit took an unfavorable orientation and, for example, *ΔS*_*IJ*_/*k*_B_ became 21.2 for all the six interfaces, the sum would be ∼127, giving rise to quite a large water-entropy loss of ∼115*k*_B_. Thus, the water entropy is strongly dependent on the orientation of the DF subunit for a packing structure of the A_3_B_3_DF complex given.

### 6.2 Packing structure of A_3_B_3_ complex with no nucleotides bound, A_3_B_3_

The packing structure of A_3_B_3_ is illustrated in Figure 5B. The hydration entropies of subcomplexes 1, 2, and 3 in this complex are included in Table 2. We find the following:

“PE of subcomplex 1”>“PE of subcomplex 2”>“PE of subcomplex 3” (11)

$$“PE\;of\;A_{E}”>“PE\;of\;A_{TP}”>“PE\;of\;A_{DP}”$$ (12)

The PE of each subcomplex is determined by that of the A subunit in it. However, Orders (11) and (12) are different from Orders (9) and (10), respectively. Unlike in A_3_B_3_DF**⋅**2ATP, A_E_-B_E_ is the most closely packed among the six A-B interfaces.

Even the structure of A_3_B_3_, the complex with no nucleotides bound and without the DF subunit, is asymmetric in the sense that the structures of the three A subunits (and the three B subunits) are different from one another. This result is in line with the feature of the crystal structure solved by Murata and coworkers (Arai et al., 2013). For F_1_-ATPase, on the other hand, it was observed using the high-speed atomic force microscopy that the structure of the α_3_β_3_ complex with no nucleotides bound and without the γ subunit is symmetric (Uchihashi et al., 2011).

### 6.3 Packing structure of A_3_B_3_ complex with two ATPs (AMP-PNPs) bound, A_3_B_3_⋅2ATP

Figure 5C illustrates the packing structure of A_3_B_3_ **⋅**2ATP. The hydration entropies of subcomplexes 1, 2, and 3 in this complex are included in Table 2. We find the following:

“PE of subcomplex 3”>“PE of subcomplex 2”>“PE of subcomplex 1” (13)

$$“PE\;of\;A_{DP}”>“PE\;of\;A_{TP}”>“PE\;of\;A_{E}”$$ (14)

The PE of each subcomplex is determined by that of the A subunit in it. Comparing the packing structures in Figures 5B, C, we notice that by the ATP binding to A_DP_ and A_TP_, A_DP_-B_DP_ and A_TP_-B_TP_ become much more closely packed and A_E_, which was the most closely packed among the three A subunits, becomes the least closely packed (in other words, A_DP_ and A_TP_ become considerably more closely packed than A_E_). Among the six A-B interfaces, A_DP_-B_DP_, A_TP_-B_TP_, and A_E_-B_E_ are relatively more closely packed as in the case of A_3_B_3_DF⋅2ATP (compare Figures 5A, C). The ATP binding to an A subunit results in not only higher PE of this A subunit but also closer packing of the interface between this A subunit and one of its adjacent B subunits.

### 6.4 Packing structure of A_3_B_3_DF complex with no nucleotides bound, A_3_B_3_DF

The packing structure of A_3_B_3_DF is illustrated in Figure 5D. The hydration entropies of subcomplexes 1, 2, and 3 in this complex are included in Table 2. We find the following:

“PE of subcomplex 2”>“PE of subcomplex 3”>“PE of subcomplex 1” (15)

$$“PE\;of\;A_{TP}”>“PE\;of\;A_{DP}”>“PE\;of\;A_{E}”$$ (16)

The PE of each subcomplex is determined by that of the A subunit in it. Among the six A-B interfaces, A_E_-B_E_ is the most closely packed. Among the six A-DF and B-DF interfaces, B_E_-DF and A_TP_-DF are relatively more closely packed. Comparing the packing structures in Figures 5B, D, we notice that by the incorporation of the DF subunit, A_DP_-B_DP_ and A_TP_-B_TP_ become more closely packed and A_E_, which was the most closely packed among the three A subunits, becomes the least closely packed.

### 6.5 Roles of ATP bindings to A_DP_ and A_TP_ and incorporation of DF subunit

Comparison among Figures 5B–D leads us to the conclusion that the change in packing structure caused by the incorporation of the DF subunit is similar to that caused by the ATP bindings to A_DP_ and A_TP_. Namely, the basal characteristics of packing structure shown in Figure 5A are constructed by the interplay of the two factors, the ATP bindings to two of the A subunits (factor 1) and the incorporation of the DF subunit (factor 2). The feature that all of A_DP_-B_DP_, A_TP_-B_TP_, and A_E_-B_E_ are quite closely packed, which is found only in Figures 5A, C, originates primarily from factor 1. Factor 2 makes a larger contribution to the establishment of Orders (9) and (10) than factor 1. A_DP_-B_DP_ becomes the most closely packed by factors 1 and 2. Another significant finding is that | *S*_1_/*k*_B_ | of the A_3_B_3_ complex decreases by ∼1,670 upon the incorporation of the DF subunit, indicating that the DF subunit largely stabilizes the A_3_B_3_ complex.

### 6.6 Packing structure of A_3_B_3_DF complex with two ADPs bound, A_3_B_3_DF⋅2ADP

The packing structure and the hydration entropies of subcomplexes 1, 2, and 3 of A_3_B_3_DF**⋅**2ADP can be compared to those of A_3_B_3_DF**⋅**2ATP in Figures 5A, F and in Table 2. Orders (9) and (10) hold in both of the two complexes. However, the packing structure of A_3_B_3_DF**⋅**2ADP possesses the following characteristics in comparison with that of A_3_B_3_DF**⋅**2ATP: The interfaces of the A_TP_-B_TP_ and A_DP_-B_DP_ pairs are looser; and the PEs of the three A subunits and the complex are considerably lower. It is important to look at the values of *ΔS*_*IJ*_/*k*_B_ for the DF-A and DF-B interfaces. The sum of the six values of *ΔS*_*IJ*_/*k*_B_ in A_3_B_3_DF**⋅**2ATP is ∼242 whereas that in A_3_B_3_DF**⋅**2ADP is only ∼122. This result is indicative that the A_3_B_3_ complex is much more stabilized in terms of the water entropy by the incorporation of the DF subunit in the presence of two ATPs bound than in that of two ADPs. On the basis of these results and the discussion on Figure 2A given above, the stability of the A_3_B_3_DF complex follows the order, A_3_B_3_DF**⋅**2ATP>A_3_B_3_DF**⋅**ATP**⋅**ADP>A_3_B_3_DF**⋅**2ADP, and the stability difference is quite large.

### 6.7 Effect of ATP or ADP on affinity between A and B subunits

Table 3 presents the water-entropy gains upon association of A_DP_ and B_DP_ or A_TP_ and B_TP_ for the three cases where nothing, ATP, and ADP bind to A_DP_ or A_TP_, respectively. In the Table, A_DP_, A_DP_-B_DP_, and A_DP_ **⋅**ATP-B_DP_, for example, denote isolated A_DP_, complex of A_DP_ and B_DP_, and complex of A_DP_ with ATP bound and B_DP_, respectively. We find that the participation of ATP or ADP in the association increases the affinity between the A and B subunits. The increase is larger for ATP than for ADP.

**TABLE 3:** Water-entropy gain upon association of B subunit and A subunit. Three cases where nothing, ATP, and ADP bind to A_DP_ or A_TP_, respectively, are considered.

Association	Water-entropy gain (*k*_B_)
A_DP_+B_DP_→A_DP_-B_DP_	368.5
A_DP_+ATP+B_DP_→A_DP_ **⋅**ATP-B_DP_	520.7
A_DP_+ADP+B_DP_→A_DP_ **⋅**ADP-B_DP_	498.9
A_TP_+B_TP_→A_TP_-B_TP_	352.0
A_TP_+ATP+B_TP_→A_TP_ **⋅**ATP-B_TP_	510.4
A_TP_+ADP+B_TP_→A_TP_ **⋅**ADP-B_TP_	416.7

### 6.8 Packing efficiency of A subunit with nothing, ATP, or ADP bound

Significant results are as follows: Upon binding of a nucleotide, the PE of an A subunit becomes higher. A single-molecule experiment for V_1_-ATPase suggested that after ATP is hydrolyzed into ADP and Pi, Pi dissociates first (Iida et al., 2019). Therefore, the chemical compounds of interest are ATP, ADP+Pi, ADP, ATP-H_2_O, and ATP(ATP-H_2_O). As for the PE of an A subunit to which a chemical compound is bound, “A(ATP)>A(ADP)>A” is shown in this study (“β(ATP)>β(ADP)>β” should be applicable to F_1_-ATPase as well). We assume that the order for V_1_-ATPase corresponding to Order (7) for F_1_-ATPase is the following:

$$\begin{array}{c} A\left(ATP\right)>A\left(ATP\left(ATP‐H_{2}O\right)\right)>A\left(ATP‐H_{2}O\right);\\ A\left(ATP‐H_{2}O\right)>A\left(ADP+Pi\right)>A\left(ADP\right)>A;A\left(ATP\right)>A \end{array}$$ (17)

Here, A(*Y*) denotes the PE of an A subunit to which chemical compound *Y* is bound, and A denotes the PE of an A subunit with nothing bound. When the chemical compound binding to an A subunit changes from ATP to ADP, for example, the PE of this A subunit becomes lower (i.e., its packing becomes looser).

In Supplementary Section S10, we briefly discuss a case where protein side chains within the protein-ligand or protein-protein binding interface are significantly flexible (Rosenzweig and Kay, 2014; Wand and Sharp, 2018), possibly more flexible than those in an isolated protein, leading to an increase in the total conformational entropy upon binding (such a case is not applicable to F_1_- and V_1_-ATPases).

## 7 Discussion

### 7.1 Physical picture of rotation mechanism of F_1_-ATPase

First, we review the physical picture recently developed by Kinoshita (Kinoshita, 2021a; Kinoshita, 2021b).

#### 7.1.1 Packing structure of α_3_β_3_γ complex in catalytic dwell state

**FIGURE 6:**
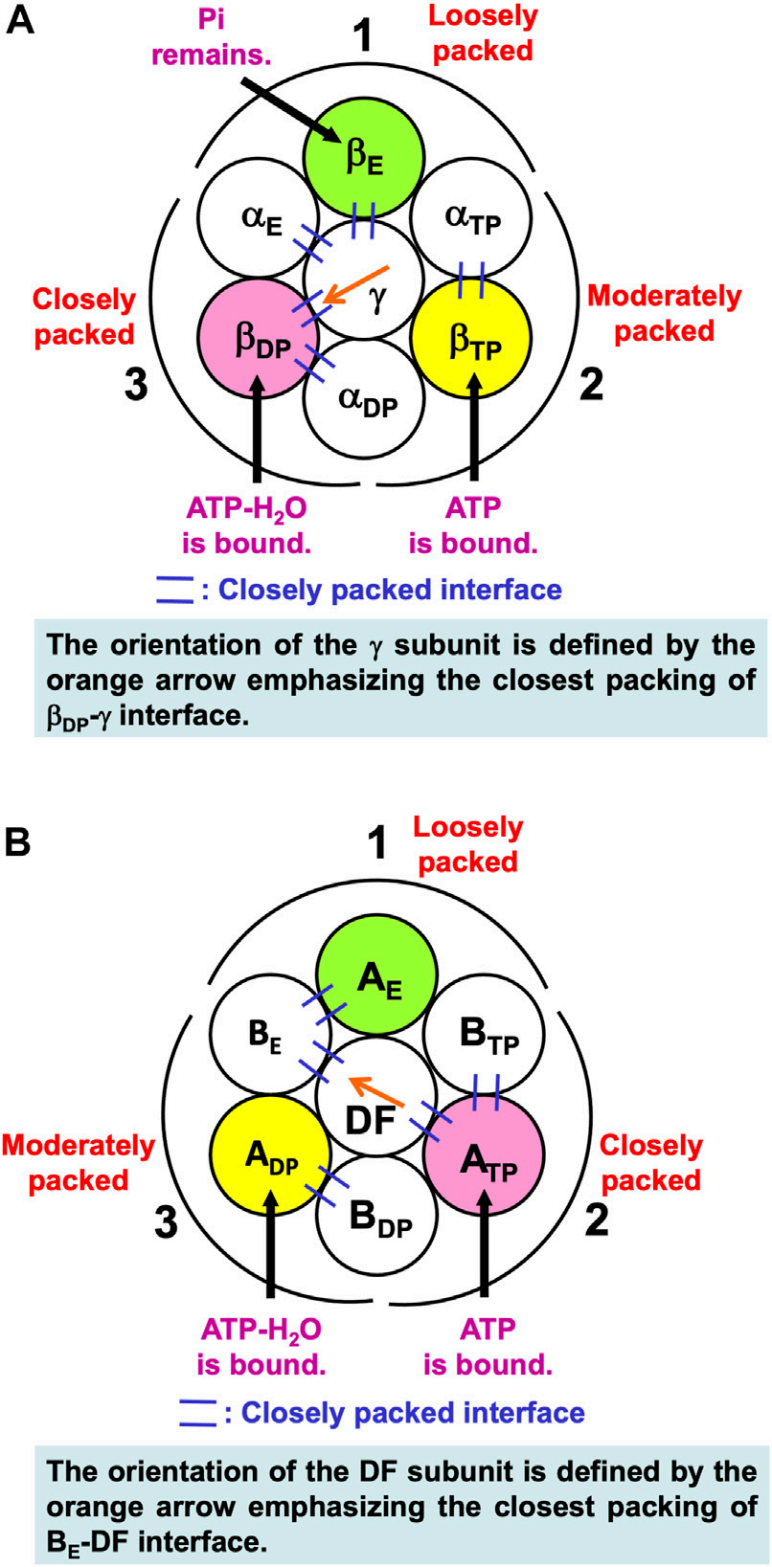
**(A)** Packing structure of catalytic dwell state of α_3_β_3_γ complex (F_1_-ATPase) stabilized in aqueous solution of ATP, ADP, and Pi. ATP-H_2_O and ATP are bound to β_DP_ and β_TP_, respectively, and Pi remains in β_E_. ATP-H_2_O denotes ATP just before the ATP hydrolysis reaction (ATP-H_2_O is an activated complex). The packings of backbones and side chains in subcomplexes 1, 2, and 3 are relatively loose, moderate, and close, respectively: The packing efficiencies (PEs) of subcomplexes 1, 2, and 3 are relatively low, moderate, and high, respectively. **(B)** Packing structure of catalytic dwell state of A_3_B_3_DF complex (V_1_-ATPase) stabilized in aqueous solution of ATP, ADP, and Pi. ATP-H_2_O and ATP are bound to A_DP_ and A_TP_, respectively, and nothing is bound to A_E_. The packings of backbones and side chains in subcomplexes 1, 2, and 3 are relatively loose, close, and moderate, respectively.

Noji and coworkers (Watanabe et al., 2010) reported that after the ATP hydrolysis into ADP and Pi within a β subunit, ADP dissociates from it first and Pi remains in it. The structures of states where any two of the nucleotides are bound to β_DP_ and β_TP_, respectively, are similar. These states are collectively referred to as the “catalytic dwell state” (Abrahams et al., 1994; Bowler et al., 2007; Masaike et al., 2008; Okuno et al., 2008; Sieladd et al., 2008). In the catalytic dwell state considered here, the initial state in the ATP hydrolysis cycle, ATP-H_2_O and ATP are bound to β_DP_ and β_TP_, respectively, and Pi remains in β_E_: It is one of the most stable states. The characteristics of its packing structure unveiled in our earlier work (Yoshidome, et al., 2011) are illustrated in Figure 6A. For comparison, the packing structure of the initial state in the ATP hydrolysis cycle, the catalytic dwell state, for V_1_-ATPase is illustrated in Figure 6B which is discussed in Section 7.2.1.

In Figure 6A, among the six β-α interfaces, β_DP_-α_DP_ and β_TP_-α_TP_ are relatively more closely packed (β_DP_-α_DP_ is the most closely packed). β_DP_-α_DP_, for example, denotes the interface between β_DP_ and α_DP_. Among the six β-γ and α-γ interfaces, β_DP_-γ, α_E_-γ, and β_E_-γ are relatively more closely packed (β_DP_-γ is the most closely packed). The same conclusion was drawn from a study using all-atom MD simulation (Ito and Ikeguchi, 2010) where the number of stable contacts within each of β-α, β-γ, and α-γ interfaces were analyzed. Here, the stable contact is defined as an inter-subunit residue pair maintaining its inter-atomic distance less than 4.5 Å for 98% of the snap shots along the MD trajectory.

#### 7.1.2 Mechanism of unidirectional rotation of central shaft in F_1_-ATPase

**FIGURE 7:**
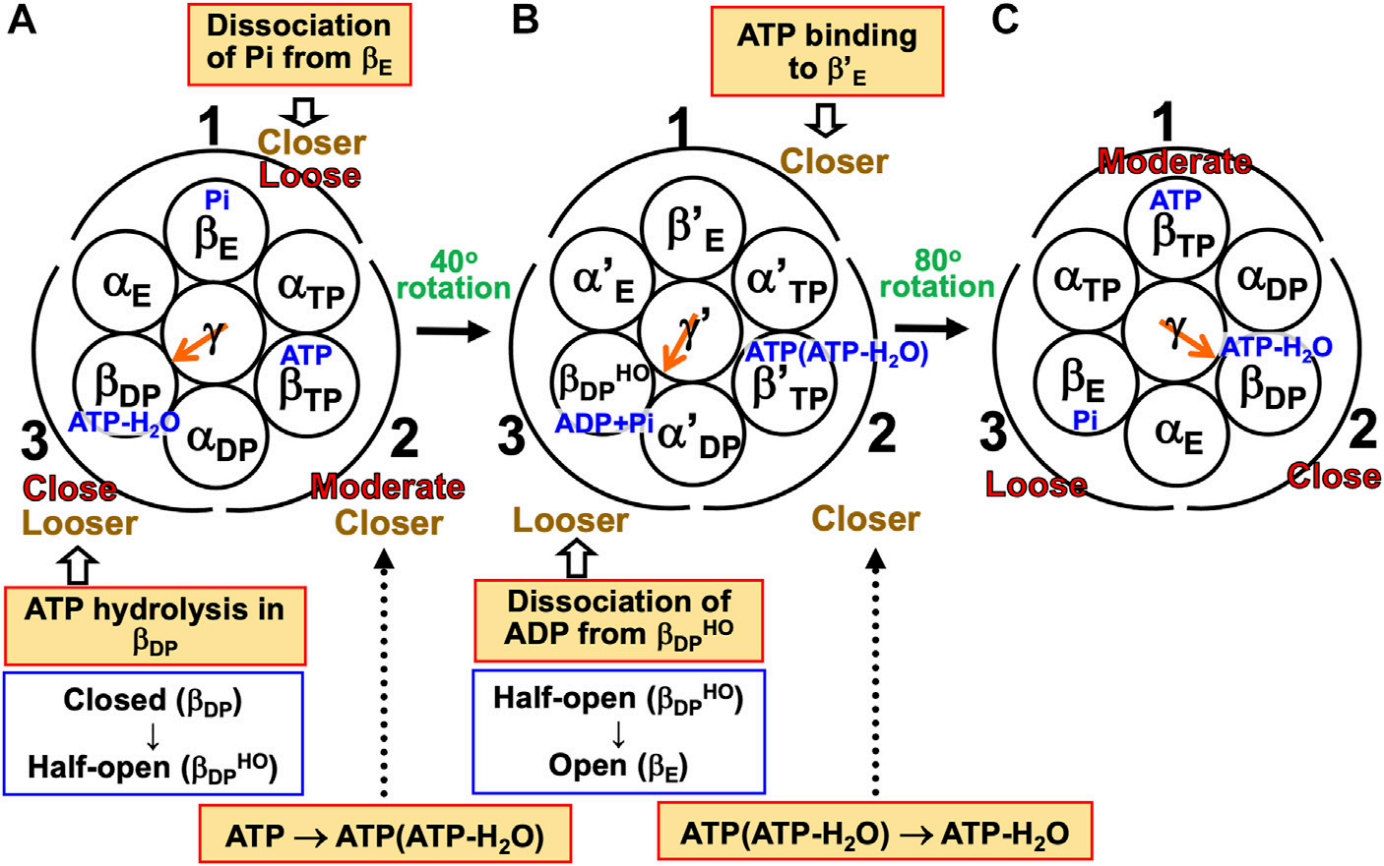
Physical picture of unidirectional rotation of γ subunit in F_1_-ATPase during one ATP hydrolysis cycle. In state **(A)**, Pi, ATP, and ATP-H_2_O are bound to β_E_, β_TP_, and β_DP_, respectively. In **(B)**, ATP(ATP-H_2_O) and ADP+Pi are bound to β′_TP_ and β_DP_^HO^, respectively. The packings of β_E_ and subcomplex 1 are loose in state **(A)** but they become closer in state change **(A)**→**(B)**. Those of β′_E_ and subcomplex 1 become further closer in **(B)**→**(C)**. Those of β_TP_ and subcomplex 1 are moderate in **(C)**. Those of β_TP_ and subcomplex 2 are moderate in **(A)** but they become closer in **(A)**→**(B)**. Those of β′_TP_ and subcomplex 2 become further closer in **(B)**→**(C)**. Those of β_DP_ and subcomplex 2 are close in **(C)**. Those of β_DP_ and subcomplex 3 are close in **(A)** but they become looser in **(A)**→**(B)**. Those of β_DP_^HO^ and subcomplex 3 become further looser in **(B)**→**(C)**. Those of β_E_ and subcomplex 3 are loose in **(C)**. As mentioned in Section 4.3, even without the γ subunit, the α_3_β_3_ complex exhibits the structural rotation (Uchihashi et al., 2011; Yoshidome et al., 2011) illustrated in this figure.

Kinoshita’s physical picture can be recapitulated as follows (Figure 7). (Recall that under the aqueous-solution condition assumed, the ATP hydrolysis cycle occurs spontaneously as the dominant process.)

(1) The α_3_β_3_γ complex is in state (A), the catalytic dwell state shown in Figure 2A, where ATP-H_2_O, ATP, and Pi are bound to β_DP_, β_TP_, and β_E_, respectively. It was experimentally observed that β_DP_, β_TP_, and β_E_ take closed, closed, and open structures, respectively (i.e., β(Nucleotide)>β(Pi) in Order (6) holds). The state changes as follows. The ATP hydrolysis (ATP-H_2_O→ADP+Pi), change of ATP to ATP(ATP-H_2_O), and Pi dissociation spontaneously occur in β_DP_, β_TP_, and β_E_, respectively. It was experimentally observed that the structure of β_DP_ becomes half-open (Watanabe et al., 2010) (i.e., β(ATP-H_2_O)>β(ADP+Pi) in Orders (6) and (7) holds): The PE of β_DP_ is lowered by the ATP hydrolysis. We denote the β subunit with this half-open structure by β_DP_^HO^. The PE of subcomplex 3 also becomes lower. ATP in β_TP_ changes to ATP(ATP-H_2_O) with the result that the PE of β_TP_ becomes higher (β(ATP(ATP-H_2_O))>β(ATP) in Order (7)). The β subunit with ATP(ATP-H_2_O) bound is denoted by β′_TP_. The PE of subcomplex 2 also becomes higher. Pi dissociates from β_E_, and the PE of β_E_ becomes higher (β>β(Pi) in Order (7)). The PE of subcomplex 1 also becomes higher. The β subunit with nothing bound is denoted by β′_E_. The γ subunit rotates by 40° during state change (A)→(B) (Yasuda et al., 1998; Shimabukuro et al., 2003; Adachi et al., 2007; Watanabe et al., 2010) in response to the change in packing structure of the α_3_β_3_ complex.
(2) The α_3_β_3_γ complex is in state (B) where ADP+Pi, ATP(ATP-H_2_O), and nothing are bound to β_DP_^HO^, β′_TP_, and β′_E_, respectively. The state changes as follows. The ADP dissociation, change of ATP(ATP-H_2_O) to ATP-H_2_O, and ATP binding spontaneously occur in β_DP_^HO^, β′_TP_, and β′_E_, respectively. It was experimentally observed that the half-open structure of β_DP_^HO^ becomes open (i.e., the packing of β_DP_^HO^ becomes looser) upon dissociation of ADP from β_DP_^HO^ (β(ADP+Pi)>β(Pi) in Orders (6) and (7) holds). The packing of subcomplex 3 also becomes looser. ATP(ATP-H_2_O)→ATP-H_2_O occurs in β′_TP_ with the result of its higher PE (β(ATP-H_2_O)>β(ATP(ATP-H_2_O)) in Order (7)). The packing of β′_TP_ and that of subcomplex 2 become closer. ATP binds to β′_E_, leading to its higher PE (β(ATP)>β in Order (7)). The packing of β′_E_ and that of subcomplex 1 become closer. The γ subunit rotates by 80° during (B)→(C) (Yasuda et al., 1998; Shimabukuro et al., 2003; Adachi et al., 2007; Watanabe et al., 2010) by responding to the change in packing structure of the α_3_β_3_ complex.
(3) The α_3_β_3_γ complex is now in state (C) where ATP-H_2_O, ATP, and Pi are bound to β_DP_, β_TP_, and β_E_, respectively. Changes of β_DP_→β_DP_^HO^→β_E_, β_TP_→β′_TP_→β_DP_, and β_E_→β′_E_→β_TP_ have occurred in subcomplexes 3, 2, and 1, respectively. The change in packing structure of the α_3_β_3_ complex has been followed by a rotation of 120° of the γ subunit in the counterclockwise direction, recovering the closely packed γ-β_DP_, γ-α_E_, and γ-β_E_ interfaces.

For (A)→(B), it was experimentally and theoretically shown that the dissociation of Pi from β_E_ as well as the ATP hydrolysis in β_DP_ induces a rotation of the γ subunit in the counterclockwise direction (Kabaleeswaran et al., 2006; Yoshidome et al., 2012). The state where only two of ATP-H_2_O, ATP(ATP-H_2_O), ATP, and ADP+Pi are bound to two of the β subunits, respectively, is one of the most stable states. Nothing is bound to one of the β subunits (β′_E_) in state (B), allowing the binding of ATP to it, which is followed by the dissociation of ADP from an adjacent β subunit (β_DP_^HO^): (B)→(C) is induced. The packing structure stabilized as state (A) is maintained during each ATP hydrolysis cycle for avoiding a water-entropy loss.

The 120° rotation splits into the two substep rotations in (A)→(B) and (B)→(C). The rotation angle in (B)→(C) is larger than that in (A)→(B), because (B)→(C) includes the ATP binding by which a relatively larger state change is caused. It is desired that the structure of state (B) be solved experimentally for elucidating why the angles of the two substep rotations are 40° and 80°, respectively (Supplementary Section S17).

#### 7.1.3 Free-energy change during one ATP hydrolysis cycle

During one ATP hydrolysis cycle, the dissociation of Pi, dissociation of ADP, binding of ATP, and ATP hydrolysis within a β subunit leads to decreases in the system free energy of Δ*G*_1_<0, Δ*G*_2_<0, Δ*G*_3_<0, and Δ*G*_4_<0, respectively. The free-energy change due to the structural reorganization of the α_3_β_3_γ complex is kept quite small for the following reasons: When the PE of one of the subcomplexes is lowered, for example, the PEs of the other two subcomplexes are heightened so that the hydration entropy of the α_3_β_3_γ complex can be kept almost constant (i.e., the loss of water entropy can be avoided) together by the reorientation of the γ subunit. Though the overall structures of F_1_-ATPase before and after the 120° rotation are the same, one ATP molecule has been hydrolyzed in bulk aqueous solution, accompanying a decrease in system free energy of Δ*G*_1_+Δ*G*_2_+Δ*G*_3_+Δ*G*_4_ = Δ*G*∼−20*k*_B_ *T*∼−12 kcal/mol at *T* = 298 K (Supplementary Section S3), the free-energy change upon the ATP hydrolysis in bulk aqueous solution. | Δ*G*_4_ | is much smaller than | Δ*G* | (Supplementary Section S11).

### 7.2 Physical picture of rotation mechanism of V_1_-ATPase

#### 7.2.1 Packing structure of A_3_B_3_DF complex in catalytic dwell state

The packing structure of the initial state in the ATP hydrolysis cycle, the catalytic dwell state, is illustrated in Figure 6B. This state in which ATP and ATP-H_2_O are bound to two of the three A subunits, respectively, is one of the most stable states. The PEs of subcomplexes 1, 2, and 3 are relatively low, high, and moderate, respectively. Among the six A-B interfaces, A_DP_-B_DP_, A_TP_-B_TP_, and A_E_-B_E_ are relatively more closely packed (A_DP_-B_DP_ is the most closely packed). Among the six A-DF and B-DF interfaces, B_E_-DF and A_TP_-DF are relatively more closely packed (B_E_-DF is the most closely packed).

#### 7.2.2 Mechanism of unidirectional rotation of central shaft in V_1_-ATPase

**FIGURE 8:**
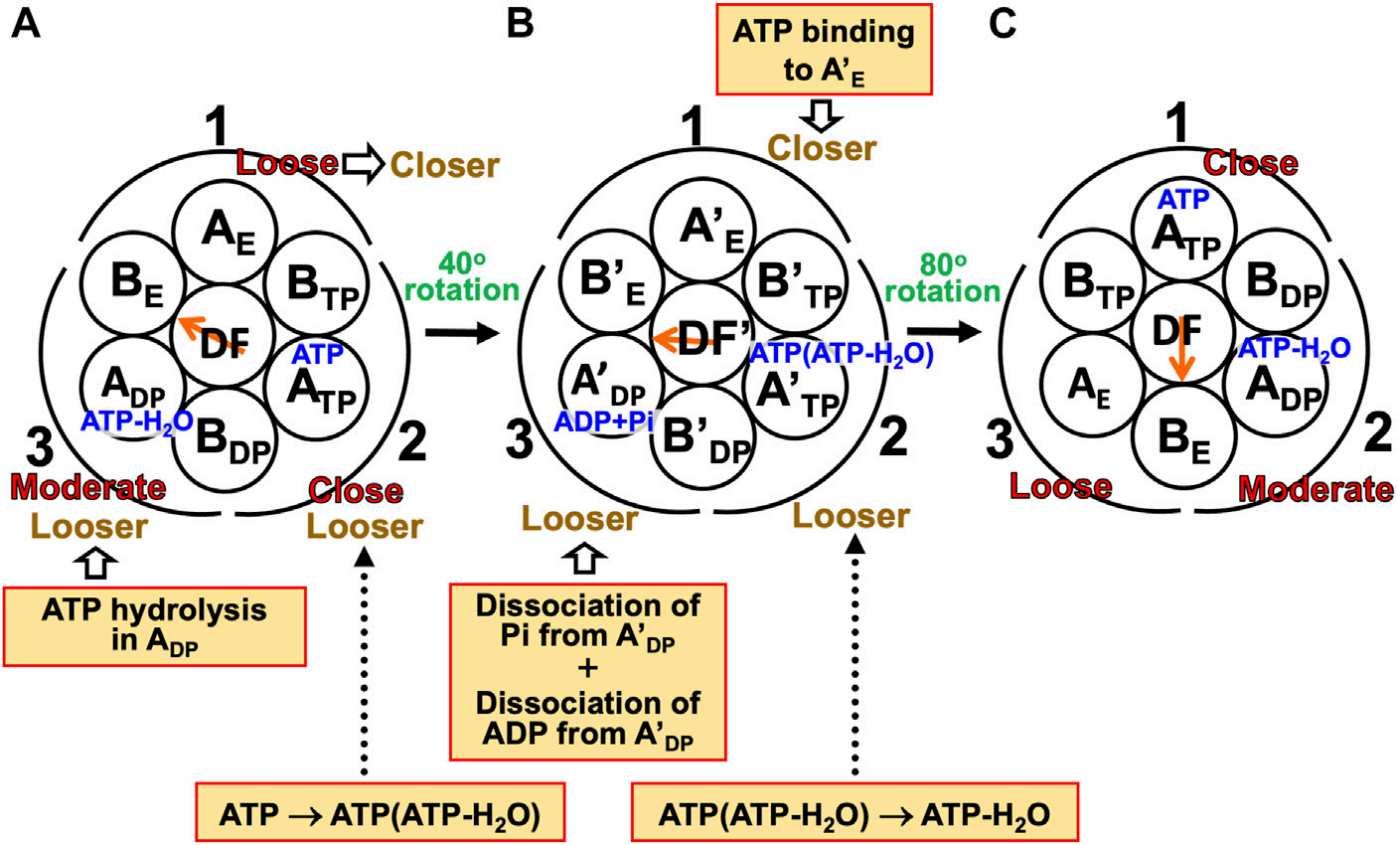
Physical picture of unidirectional rotation of DF subunit in V_1_-ATPase during one ATP hydrolysis cycle. In state **(A)**, ATP and ATP-H_2_O are bound to A_TP_ and A_DP_, respectively. In **(B)**, ATP(ATP-H_2_O) and ADP+Pi are bound to A’_TP_ and A’_DP_, respectively. ATP-H_2_O denotes ATP just before the ATP hydrolysis reaction, and ATP(ATP-H_2_O) is an intermediate between ATP and ATP-H_2_O (ATP(ATP-H_2_O) and ATP-H_2_O are the activated complexes). The packings of A_E_ and subcomplex 1 are loose in state **(A)** but they become closer in state change **(A)**→**(B)**. Those of A’_E_ and subcomplex 1 become further closer in **(B)**→**(C)**. Those of A_TP_ and subcomplex 1 are close in **(C)**. Those of A_TP_ and subcomplex 2 are close in **(A)** but they become looser in **(A)**→**(B)**. Those of A’_TP_ and subcomplex 2 become further looser in **(B)**→**(C)**. Those of A_DP_ and subcomplex 2 are moderate in **(C)**. Those of A_DP_ and subcomplex 3 are moderate in **(A)** but they become looser in **(A)**→**(B)**. Those of A’_DP_ and subcomplex 3 become further looser in **(B)**→**(C)**. Those of A_E_ and subcomplex 3 are loose in **(C)**.

A single-molecule experiment for V_1_-ATPase showed that the 120° rotation of the DF subunit comprises 40° and 80° substeps (Iida et al., 2019). It also suggested the emergence of a stabilized state with three nucleotides bound, but we do not follow this suggestion for the reason mentioned above. On the basis of the calculation results described above, we can suggest the following rotation mechanism for V_1_-ATPase (Figure 8).

(1) The A_3_B_3_DF complex is in state (A) where ATP-H_2_O, ATP, and nothing are bound to A_DP_, A_TP_, and A_E_, respectively, the catalytic dwell state shown in Figure 6B. The state changes as follows. The ATP hydrolysis and the change of ATP to ATP(ATP-H_2_O) spontaneously occur in A_DP_ and A_TP_, respectively. The PE of A_DP_ is lowered by the ATP hydrolysis, ATP-H_2_O→ADP+Pi, due to A(ATP-H_2_O)>A(ADP+Pi) in Order (17). The A subunit with ADP+Pi bound is denoted by A’_DP_. The PE of subcomplex 3 also becomes lower. ATP in A_TP_ changes to ATP(ATP-H_2_O) with the result that the PE of A_TP_ becomes lower due to A(ATP)>A(ATP(ATP-H_2_O)) in Order (17). The A subunit with ATP(ATP-H_2_O) bound is denoted by A’_TP_. The PE of subcomplex 2 also becomes lower. The lowering of the PEs of A_DP_ and A_TP_ induces the heightening of the PE of A_E_. The PE of subcomplex 1 also becomes higher. A_E_ changes to A’_E_. The DF subunit rotates by 40° during state change (A)→(B) by responding to the change in packing structure of the A_3_B_3_ complex.
(2) The A_3_B_3_DF complex is in state (B) where ADP+Pi, ATP(ATP-H_2_O), and nothing are bound to A’_DP_, A’_TP_, and A’_E_, respectively. The state changes as follows. Pi and ADP dissociate from A’_DP_ with the result of its lower PE due to A(ADP+Pi)>A(ADP)>A in Order (17) (the PE becomes lower upon Pi dissociation and it becomes further lower upon ADP dissociation). The packing of A’_DP_ and that of subcomplex 3 become looser. The change, ATP(ATP-H_2_O)→ATP-H_2_O, occurs in A’_TP_ with the result of its lower PE (A(ATP(ATP-H_2_O))>A(ATP-H_2_O) in Order (17)). The packing of A’_TP_ and that of subcomplex 2 become looser. ATP binds to A’_E_, leading to its higher PE (A(ATP)>A in Order (17)). The packing of A’_E_ and that of subcomplex 1 become closer. The DF subunit rotates by 80° during (B)→(C) in response to the change in packing structure of the A_3_B_3_ complex.
(3) The A_3_B_3_DF complex is now in state (C) where ATP-H_2_O, ATP, and nothing bound to A_DP_, A_TP_, and A_E_, respectively. Changes of A_DP_→A’_DP_→A_E_, A_TP_→A’_TP_→A_DP_, and A_E_→A’_E_→A_TP_ have occurred in subcomplexes 3, 2, and 1, respectively. The change in packing structure of the A_3_B_3_ complex has been followed by a rotation of 120° of the DF subunit in the counterclockwise direction, recovering the closely packed DF-A_TP_ and DF-B_E_ interfaces.

During each ATP hydrolysis cycle, the stabilized state of V_1_-ATPase, the state where only two of ATP-H_2_O, ATP(ATP-H_2_O), ATP, and ADP+Pi are bound to two of the three A subunits, respectively, is maintained. If one of the two chemical compounds bound is replaced by ADP, the stability becomes considerably lower.

#### 7.2.3 Occurrence of ATP hydrolysis reaction in not A_TP_ but A_DP_

Here, we assume, unlike in the case of state (A) shown in Figure 8, that ATP, ATP-H_2_O, and nothing are bound to A_DP_, A_TP_, and A_E_, respectively, and the ATP hydrolysis reaction occurs in A_TP_ in (A)→(B). If the packings of backbones and side chains in subcomplexes 2 and 3 are relatively close and moderate, respectively, the PE of A_TP_ should be higher than that of A_DP_ [i.e., A(ATP-H_2_O)>A(ATP)]. Hence, we assume that Order (7) where “β” is replaced by “A” holds. The DF subunit would then rotate in the inverse (i.e., clockwise) direction as illustrated in Supplementary Figure S2. On the other hand, with the assumption that the packings of backbones and side chains in subcomplexes 2 and 3 are relatively moderate and close, respectively, and Order (17) holds, the DF subunit would again rotate in the inverse direction as illustrated in Supplementary Figure S3. These results can readily be understood, because if the ATP hydrolysis reaction occurs in A_TP_, A_TP_ with ATP-H_2_O bound changes to A_E_ with nothing bound upon the 120° rotation, indicating the inverse rotation. Thus, the occurrence of ATP hydrolysis reaction in A_DP_ as well as the packing structure of the A_3_B_3_DF complex is highly relevant to the rotation of central shaft in the normal (i.e., counterclockwise) direction observed in a single-molecule experiment (Suzuki et al., 2016).

We then discuss why the ATP hydrolysis reaction occurs in not A_TP_ but A_DP_. For A_3_B_3_⋅2ATP and A_3_B_3_DF⋅2ATP, we calculate the values of the distance between E261 and ATP, *L*, in A_DP_ and A_TP_. E261 is essential in the ATP hydrolysis reaction as shown in previous works (Liu et al., 1997; Dittrich et al., 2003). *L* is closely related to the PE of the A-B interface. The calculation result is given in Table 4. that Comparing the packing structures shown in Figures 5A, C, we notice that A_TP_-B_TP_ is more closely packed than A_DP_-B_DP_ and *L* in A_TP_ is shorter in A_3_B_3_⋅2ATP, whereas the opposite is true in A_3_B_3_DF⋅2ATP. In A_3_B_3_⋅2ATP, since *L*’s in A_DP_ and A_TP_ are almost the same, the ATP hydrolysis reaction can occur either in A_DP_ or A_TP_ with almost equal probability. However, the incorporation of the DF subunit makes *L* in A_DP_ shorter but *L* in A_TP_ longer, which can lead to the preferential occurrence of the hydrolysis reaction in A_DP_. As for the packing structure of the α_3_β_3_γ complex (F_1_-ATPase), since β_DP_-α_DP_ is more closely packed than β_TP_-α_TP_ as observed in Figures 5A, E, it should be the case that *L* in β_DP_ is shorter. Hence, the ATP hydrolysis reaction occurs in β_DP_, which is commensurate with the discussion made by Walker and coworkers (Bowler et al., 2007).

**TABLE 4:** Distance between E261 and ATP, *L*. *L* is defined as the distance between centers of the phosphorus atom (P) in the γ-phosphate of ATP and the carbon atom (C) in the carboxyl group of the side chain of E261. Shorter *L* implies higher probability of occurrence of the ATP hydrolysis reaction in the A subunit.

A subunit	*L* (Å)
A_DP_ in A_3_B_3_ **⋅**2ATP	5.98
A_TP_ in A_3_B_3_ **⋅**2ATP	5.73
A_DP_ in A_3_B_3_DF**⋅**2ATP	5.69
A_TP_ in A_3_B_3_DF**⋅**2ATP	6.34

On the basis of the above discussion, we hypothesize for V_1_-ATPase that the incorporation of the central shaft in the A_3_B_3_ complex plays an essential role in the occurrence of ATP hydrolysis reaction in A_DP_. Without the central shaft, the ATP hydrolysis reaction occurs either in A_DP_ or A_TP_ with almost equal probability, giving rise to unattainability of the unidirectional rotation. Unlike in the case of the α_3_β_3_ complex (without the central shaft) for F_1_-ATPase, there is a possibility that the structural rotation of the A_3_B_3_ complex may not occur.

### 7.3 Leading roles played by water entropy effect

Kinoshita (Kinoshita, 2021a; Kinoshita, 2021b) claimed that water plays leading roles in the functional expression of F_1_-ATPase. In what follows, we refine his claim and describe it in detail. The statements are applicable to V_1_-ATPase when “α”, “β”, and “γ” are replaced by “B”, “A”, and “DF”, respectively.

#### 7.3.1 Entropic torque generated by water for rotating central shaft

First, we consider F-actin and a myosin head [e.g., myosin subfragment 1 (S1) (Kitamura et al., 1999)]. We assume that the position and structure of F-actin are fixed. Water (the aqueous solution is referred to simply as water when the water roles are emphasized) is included in the system. The free energy of the system is denoted by *G*_System_. (Since a structural change of a protein and a protein-ligand binding occur with the system volume being almost unchanged even under the isobaric condition (Yoshidome et al., 2008; Yamada et al., 2019; Inoue et al., 2020a), we use “free energy” instead of “Gibbs free energy” or “Helmholtz free energy.”) The structure and position of the myosin head change in the direction of lowering *G*_System_. For a prescribed structure of the myosin head, *G*_System_ is variable depending on the position of the myosin head, and *G*_System_ can be represented as a function of the Cartesian coordinates of the center of gravity of the myosin head, (*x*, *y*, *z*). The origin of the coordinate system (0, 0, 0) is chosen to be, for example, the left edge of F-actin. The *x*-axis is taken to be in the direction of forward movement of the myosin head along F-actin. *Φ*(*x*, *y*, *z*) defined as

$$\Phi \left(x,y,z\right)=G_{System}\left(x,y,z\right)-G_{System}\left(+\infty ,+\infty ,+\infty \right)$$ (18)

represents the spatial distribution of the potential of mean force, the water-mediated potential, between F-actin and the myosin head (Kinoshita, 2021a; Kinoshita, 2021b). The mean force acting on the myosin head, ***f***, is expressed as

$$f=f_{x}i+f_{y}j+f_{z}k,f_{x}=-\partial \Phi /\partial x,f_{y}=-\partial \Phi /\partial y,f_{z}=-\partial \Phi /\partial z$$ (19)

where **i**, **j**, and **k** denote the direction unit vectors. ***f*** (*x*_0_, *y*_0_, *z*_0_) represents the force induced between F-actin and the myosin head averaged over all the possible configurations of water molecules in the entire system with (*x*, *y*, *z*) being fixed at (*x*_0_, *y*_0_, *z*_0_). Thus, a potential or force field acts on the myosin head due to the presence of F-actin near it. The myosin head makes a Brownian motion in the absence of F-actin whereas its motion is influenced by the potential or force field in the presence of F-actin.

The potential or force field is strongly dependent on the structure of the myosin head which is variable depending on the chemical compound (nothing, ATP, ADP+Pi, or ADP) bound to the myosin head. Upon a structural change of the myosin head, *Φ*(*x*, *y*, *z*) and ***f*** (*x*, *y*, *z*) also exhibit significantly large changes. That is, the potential or force field acting on myosin exhibits sequential changes during the ATP hydrolysis cycle (binding of ATP to the myosin head, hydrolysis of ATP into ADP and Pi in it, release of Pi from it, and release of ADP from it) which can lead to the unidirectional movement of the myosin head along F-actin as explained in Supplementary Section S13.

We then consider F_1_-ATPase. The system free energy is denoted by *G*_System_. For a structure of the α_3_β_3_ complex given, *G*_System_ is a function of the rotation angle *θ* defined for the γ subunit in the counterclockwise direction with the origin suitably chosen. The structure of the γ subunit is also variable depending on *θ*. Denoting the mean torque acting on the γ subunit by *τ*(*θ*), we can express it as

$$\tau \left(\theta \right)=-\left(\partial G_{System}/\partial \theta \right)$$ (20)

*τ*(*θ*_0_) represents the torque acting on the γ subunit, which is averaged over all the possible translational configurations of water molecules in the entire system with *θ* being fixed at *θ*_0_. Since the dependence of *G*_System_ on *θ* is strong, *τ*(*θ*) is also strong. *G*_System_ sharply becomes minimum at *θ* = *θ*_min_ and *τ*(*θ*_min_) = 0. During the ATP hydrolysis cycle, the chemical compounds bound to the three β subunits, the structure of the α_3_β_3_ complex, and *τ*(*θ*) and *θ*_min_ exhibit sequential changes. This leads to the unidirectional rotation of the γ subunit (see the discussions in Section 7.1.2, Section 7.3.3, and Section 7.3.4).

As detailed in Supplementary Section S13, when we discuss *ΔG*_System_, the water entropy *S*_Water_ can be regarded as a principal component of *G*_System_:

$${\Delta G}_{System}∼-{T\Delta S}_{Water}$$ (21)

Here, *ΔX* signifies the change in *X* caused by structural and positional changes of the myosin head or by structural changes of the α_3_β_3_ complex and the γ subunit and an orientational change of the γ subunit. We can take the view that the structure and position of the myosin head or the structure of the α_3_β_3_ complex and the structure and orientation of the γ subunit are determined so that the water entropy can be increased. Thus, *G*_System_ in Eqs 18, 20 can be replaced by −*TΔS*_Water_. *τ*(*θ*) can be referred to as the “entropic torque”.

Taken together, the force moving the myosin head and the torque rotating the central shaft are generated by water. No input of chemical free energy of ATP is required for the force or torque generation. It is just that in an ATP hydrolysis cycle the system free energy decreases by the free-energy change upon the ATP hydrolysis in bulk aqueous solution. We note, however, that this never means that ATP plays no significant roles. In the absence of ATP, the unidirectionality of the movement or rotation is not achievable: Once the myosin head with a prescribed structure, for example, is stabilized in the position where *G*_System_ is minimized, its movement is stopped unless it can overcome the free-energy barrier because the potential or force field is not changed. The presence of ATP is certainly indispensable as a trigger of the force or torque generation. For actomyosin, the extension of the physical picture to the unidirectional movement of myosin V, a double-headed myosin, along F-actin is to be considered in future studies. In any case, the conformation of actomyosin (i.e., structures, orientations, and positions of myosin and F-actin) or that of F_1_-ATPase is perturbed by the ATP hydrolysis cycle (Section 3.1) but adjusted so that the system free energy can be lowered, which leads to the unidirectional movement or rotation.

When *C*_ATP_ is sufficiently higher than *C*_ADP_ and *C*_Pi_, the overall reaction is unidirectional: It is not ATP synthesis but ATP hydrolysis. F_1_-ATPase is involved in the ATP hydrolysis reaction through a cycle comprising ATP binding to it, hydrolysis of ATP into ADP and Pi in it, and dissociation of ADP and Pi from it, with the result that the structure of the α_3_β_3_ complex undergoes sequential changes during the cycle. It is not the chemical free energy of ATP but the effect of translational, configurational entropy of water that generates the torque for rotating the γ subunit. ATP just triggers the structural change of the α_3_β_3_ complex. The ATP hydrolysis cycle and the maintenance of the stabilized state of F_1_-ATPase, where only two of ATP-H_2_O, ATP(ATP-H_2_O), ATP, and ADP+Pi are bound to two of the β subunits, respectively, are in concert with each other (Figure 7). The ATP binding can occur when the nucleotide-binding site of one of the three β subunits (β′_E_ in state (B)) is vacant. Upon the ATP binding, ADP in one of the other two β subunits (β_DP_^HO^ in state (B)) needs to dissociate from it.

Here, we discuss the following two cases: Even in the absence of ATP, when the trailing head of myosin V is artificially detached from F-actin, it makes the forward (unidirectional) movement (case 1) (Kodera and Ando, 2014; Ngo et al., 2016); and in the absence of ATP, the rotation of the central shaft in F_1_-ATPase does not occur (case 2) (Noji et al., 1997). These two cases can be explained as follows.

Case 1: A myosin head or the trailing head of myosin V is detached from F-actin when its structure is changed upon ATP binding to it (Supplementary Section S13). In the absence of ATP, the detachment does not occur, and it keeps binding to F-actin. Even in the absence of ATP, when the myosin head is artificially detached, the potential or force field acting on it is changed, which moves it in the forward direction (Supplementary Section S13). When the trailing head is artificially detached, the conformation of actomyosin is perturbed and adjusted so that the system free energy can be lowered, leading to the forward movement.

Case 2: In the absence of ATP, the sequential changes in chemical compounds bound to the three β subunits are also absent. None of the structure of the α_3_β_3_ complex, *τ*(*θ*), and *θ*_min_ exhibits the sequential changes. Therefore, the rotation of the central shaft does not occur.

#### 7.3.2 Tight coupling between ATP hydrolysis or synthesis reaction and rotation of central shaft in normal or inverse direction

Kinoshita showed for F_1_-ATPase that the following four rotation modes, modes (i) through (iv), can be elucidated within the same theoretical framework (Kinoshita, 2021a). Three of them are (i) rotation in the normal (counterclockwise) direction; (ii) rotation in the inverse (clockwise) direction; and (iii) rotations in random directions where the normal and inverse directions take place with the same frequency. Modes (i), (ii), and (iii) are induced under the aqueous-solution conditions that the overall reaction is ATP hydrolysis, it is ATP synthesis, and ATP hydrolysis and synthesis reactions are in equilibrium, respectively. The last mode is (iv) rotation in the inverse direction even under the aqueous-solution condition that the overall reaction should be ATP hydrolysis, which is driven by sufficiently strong external torque imposed on the γ subunit, leading to the occurrence of ATP synthesis as the overall reaction.

As described in Supplementary Section S3, even under the aqueous-solution condition that the overall reaction is ATP hydrolysis, the frequency of ATP synthesis reaction is not zero. In fact, it was experimentally observed in mode (i) that rotation in the inverse direction accompanying ATP synthesis takes place with low frequency (Yasuda et al., 1998). Mode (ii) is useful as a thought experiment. In mode (iv) the overall reaction becomes ATP synthesis even under the aqueous-solution condition that the overall reaction should be ATP hydrolysis. Kinoshita’s physical pictures (Kinoshita, 2021a) for modes (ii) and (iv) are revisited in Section 7.3.3 and Section 7.3.4, respectively. Taken together, the ATP hydrolysis or synthesis reaction is tightly coupled to the rotation in the normal or inverse direction through the water-entropy effect.

#### 7.3.3 Inverse rotation of γ subunit in F_1_-ATPase with ATP synthesis

**FIGURE 9:**
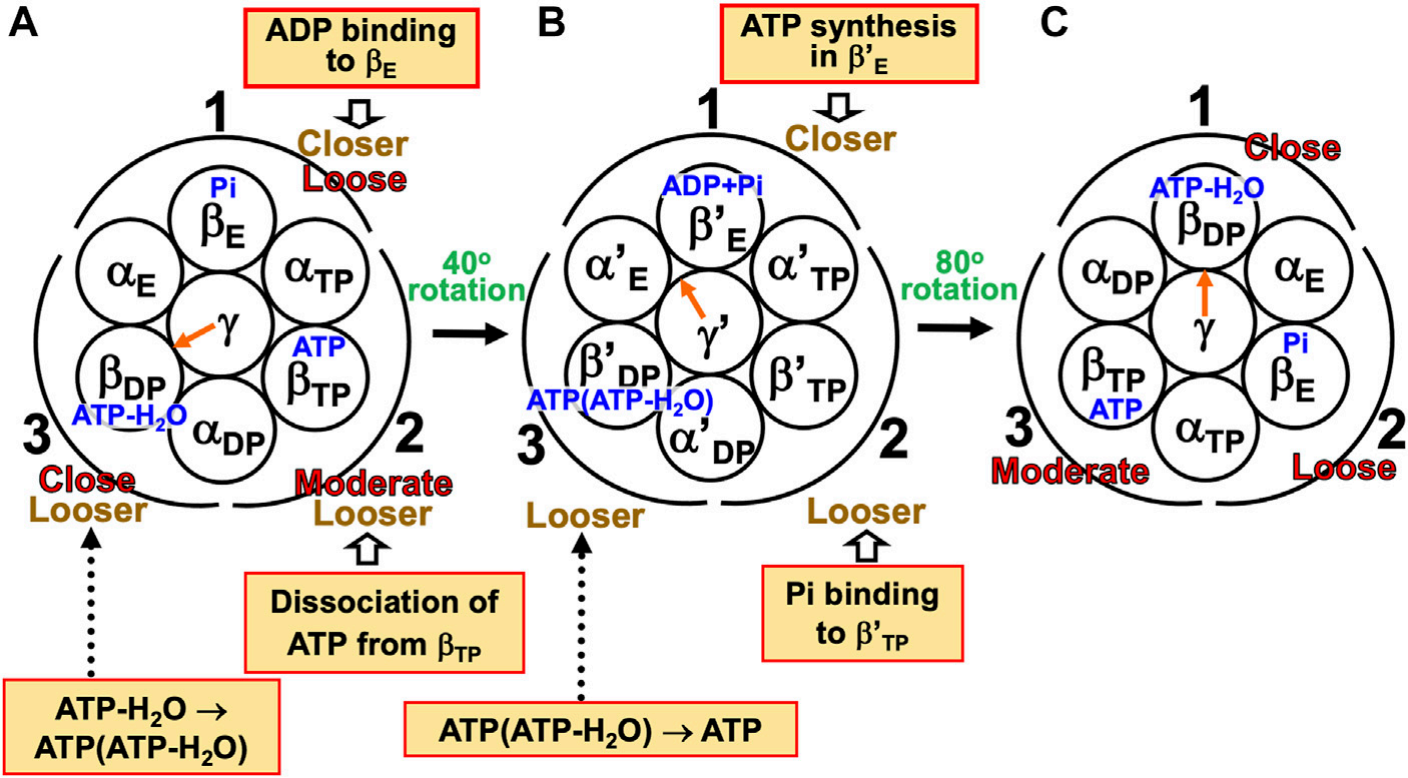
Physical picture of unidirectional rotation of γ subunit in F_1_-ATPase during one ATP synthesis cycle. In state **(A)**, Pi, ATP, and ATP-H_2_O are bound to β_E_, β_TP_, and β_DP_, respectively. In **(B)**, ADP+Pi and ATP(ATP-H_2_O) are bound to β′_E_ and β′_DP_, respectively. The packings of β_E_ and subcomplex 1 are loose in state **(A)** but they become closer in state change **(A)**→**(B)**. Those of β′_E_ and subcomplex 1 become further closer in **(B)**→**(C)**. Those of β_DP_ and subcomplex 1 are close in **(C)**. Those of β_TP_ and subcomplex 2 are moderate in **(A)** but they become looser in **(A)**→**(B)**. Those of β′_TP_ and subcomplex 2 become further looser in **(B)**→**(C)**. Those of β_E_ and subcomplex 2 are loose in **(C)**. Those of β_DP_ and subcomplex 3 are close in **(A)** but they become looser in **(A)**→**(B)**. Those of β′_DP_ and subcomplex 3 become further looser in **(B)**→**(C)**. Those of β_TP_ and subcomplex 3 are moderate in **(C)**.

The mechanism of inverse, unidirectional rotation of the γ subunit accompanying ATP synthesis can be outlined as follows (Figure 9) (Kinoshita, 2021a). Under the aqueous-solution condition assumed, Δ*G* defined in Supplementary Section S3 is positive, and the free-energy change becomes negative for ATP synthesis. The ATP synthesis cycle composed of binding of ADP and Pi to, synthesis of ADP and Pi into ATP in, and dissociation of ATP from the α_3_β_3_γ complex occurs spontaneously for lowering the system free energy.

(1) The α_3_β_3_γ complex is in state (A) where ATP-H_2_O, ATP, and Pi are bound to β_DP_, β_TP_, and β_E_, respectively (i.e., the catalytic dwell state shown in Figure 6A). The state changes as follows. The change of ATP-H_2_O to ATP(ATP-H_2_O), ATP dissociation, and ADP binding spontaneously occur in β_DP_, β_TP_, and β_E_, respectively. The PE of β_DP_ is lowered by the change due to β(ATP-H_2_O)>β(ATP(ATP-H_2_O)) in Order (7). The PE of subcomplex 3 also becomes lower. ATP dissociates from β_TP_ with the result that the PE of β_TP_ becomes lower due to β(ATP)>β in Order (7). The PE of subcomplex 2 also becomes lower. ADP binds to β_E_, and the PE of β_E_ becomes higher (β(ADP+Pi)>β(Pi) in Order (7)). The β subunits to which ADP+Pi, nothing, and ATP(ATP-H_2_O) are bound are denoted by β′_E_, β′_TP_, and β′_DP_, respectively. The γ subunit rotates by 80° in the inverse direction during state change (A)→(B) in response to the change in packing structure of the α_3_β_3_ complex.
(2) The α_3_β_3_γ complex is in state (B). The state changes as follows. The change of ATP(ATP-H_2_O) to ATP, Pi binding, and ATP synthesis (ADP+Pi→ATP-H_2_O) spontaneously occur in β′_DP_, β′_TP_, and β′_E_, respectively. ATP(ATP-H_2_O)→ATP occurs in β′_DP_ with the result of its lower PE due to β(ATP(ATP-H_2_O))>β(ATP) in Order (7). The packing of β′_DP_ and that of subcomplex 3 become looser. Pi binds to β′_TP_ with the result of its lower PE (β>β(Pi) in Order (7)). The packing of β′_TP_ and that of subcomplex 2 become looser. The ATP synthesis occurs in β′_E_, leading to its higher PE (β(ATP-H_2_O)>β(ADP+Pi) in Order (7)). The packings of β′_E_ and subcomplex 1 become closer. The γ subunit rotates by 40° in the inverse direction during (B)→(C) by responding to the change in packing structure of the α_3_β_3_ complex.
(3) The α_3_β_3_γ complex is now in state (C) where ATP-H_2_O, ATP, and Pi are bound to β_DP_, β_TP_, and β_E_, respectively. Changes of β_DP_→β′_DP_→β_TP_, β_TP_→β′_TP_→β_E_, and β_E_→β′_E_→β_DP_ have occurred in subcomplexes 3, 2, and 1, respectively. The change in packing structure of the α_3_β_3_ complex has been followed by a rotation of 120° of the γ subunit in the clockwise direction, recovering the closely packed γ-β_DP_, γ-α_E_, and γ-β_E_ interfaces. Upon the 120° rotation, one ATP molecule is synthesized in bulk aqueous solution, accompanying a decrease in system free energy.

#### 7.3.4 Inverse rotation of γ subunit in F_1_-ATPase forcibly executed by imposing external torque

We discuss mode (iv) (Toyabe et al., 2011; Toyabe and Muneyuki, 2015). The system is under the aqueous-solution condition that the overall reaction should be ATP hydrolysis: *ΔG* defined as Eq. (S10) is negative. As the external torque increases from zero, the frequency of the inverse rotation accompanying the ATP synthesis becomes higher. When the external torque multiplied by 2π/3 (120°) is −*ΔG* > 0, F_1_-ATPase is in the stalled state where the frequency of the normal rotation accompanying the ATP hydrolysis becomes equal to that of the inverse rotation (Toyabe and Muneyuki, 2015). As it becomes further stronger, the frequency of the normal rotation accompanying the ATP hydrolysis becomes lower. Eventually, the inverse rotation occurs exclusively.

The packing structure of the α_3_β_3_ complex prescribed, the water entropy is strongly dependent on the orientation of the γ subunit. In mode (i), the orientation of the γ subunit is changed in accordance with the change in packing structure of the α_3_β_3_ complex induced by the ATP hydrolysis cycle. In mode (iv), on the other hand, the packing structure of α_3_β_3_ complex is changed in accordance with the orientational change of the γ-subunit. It is important to note that the change in packing structure is controlled by the variation of chemical compounds bound to the three β subunits.

**FIGURE 10:**
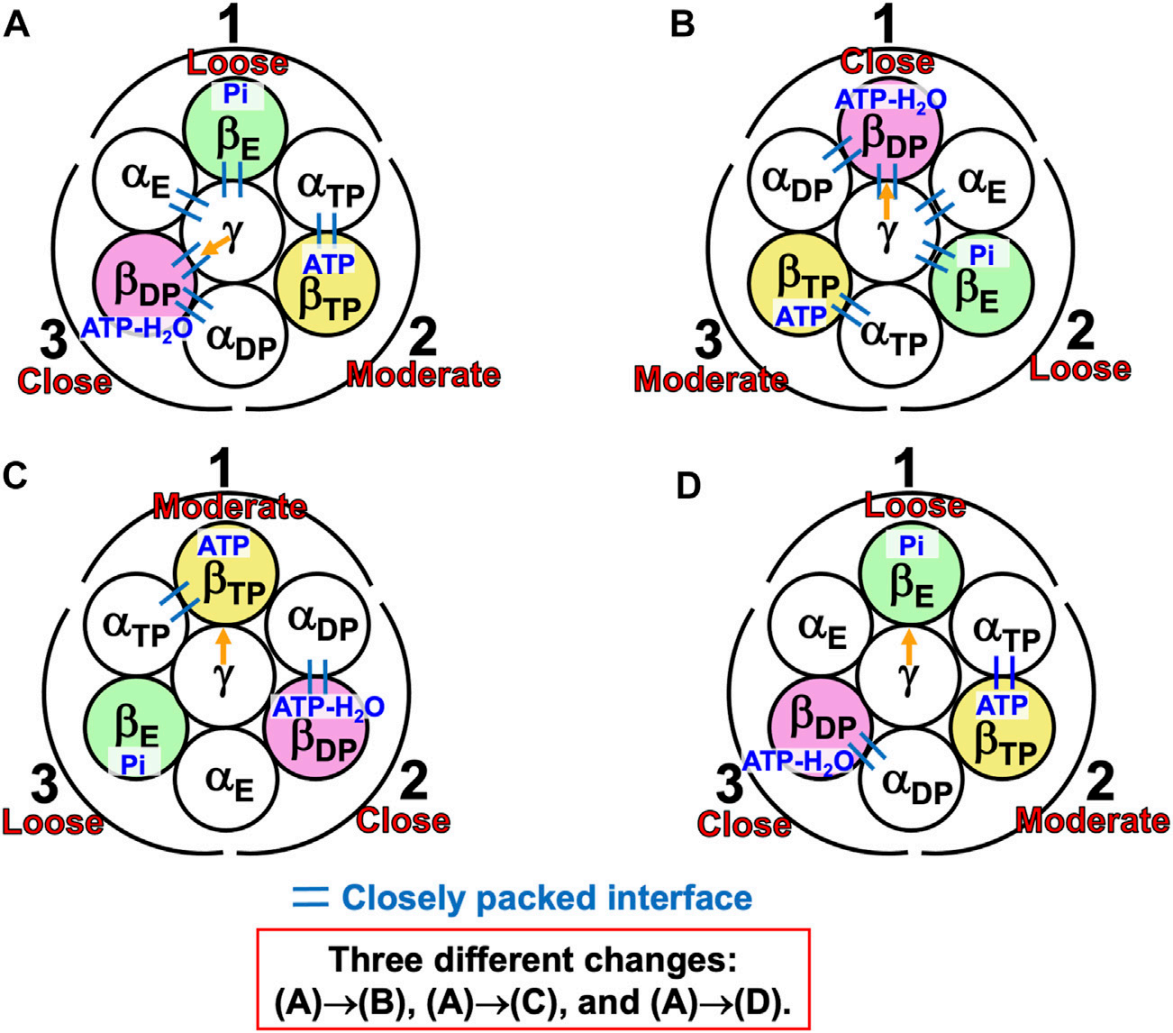
Three different state changes per cycle, **(A)**→**(B)**, **(A)**→**(C)**, and **(A)**→**(D)**. The aqueous solution is under the condition that the ATP hydrolysis reaction should occur. However, the γ subunit is forcibly rotated it in the inverse direction by a sufficiently strong external torque imposed on it. The α_3_β_3_γ complex in state **(B)** corresponds to that in state **(C)** shown in Figure 9. The α_3_β_3_ complex in state **(C)** corresponds to that in state **(C)** shown in Figure 7.

We consider three different state changes per cycle, (A)→(B), (A)→(C), and (A)→(D) as illustrated in Figure 10. In states (C) and (D), since the γ subunit is not orientated toward β_DP_, the close packings of β_DP_-γ, α_E_-γ, and β_E_-γ are lost. State change (A)→(B), during which chemical compounds bound to the three β subunits vary as in Figure 9 and the ATP synthesis reaction occurs, does not cause a water-entropy loss. Since the system is under the aqueous-solution condition that the overall reaction should be ATP hydrolysis, the ATP synthesis cycle composed of binding of ADP and Pi to, synthesis of ADP and Pi into ATP in, and dissociation of ATP from the α_3_β_3_γ complex, which occurs as the overall process, gives rise to an increase in the system free energy. This is not thermodynamically inconsistent because there is an energy input per cycle through the external torque. During (A)→(C), chemical compounds bound to the three β subunits vary as in Figure 7 and the ATP hydrolysis occurs. ATP binding to, ATP hydrolysis in, and ADP and Pi dissociation from the α_3_β_3_γ complex leads to a decrease in the system free energy. During (A)→(D), chemical compounds bound to the three β subunits remain unchanged. Neither of the ATP hydrolysis and synthesis reactions occurs, which does not contribute to a change in system free energy. Upon (A)→(C) or (A)→(D), an acceptably large loss of water entropy is unavoidably caused. The free-energy increase arising from this water-entropy loss is much larger than the free-energy change due to the ATP hydrolysis or synthesis reaction (Section 6.1). Therefore, (A)→(C) and (A)→(D) are prohibitive, and (A)→(B) occurs exclusively in mode (iv). See Supplementary Section S14 as well.

Muneyuki and coworkers (Toyabe and Muneyuki, 2015; Tanaka et al., 2020) performed intriguing experiments for a mutant of F_1_-ATPase where a mutation of E190D is made for every β subunit. They observed that even by a weak external torque, the γ subunit often rotates in the inverse direction without the occurrence of ATP synthesis reaction (i.e., state change (A)→(D) in Figure 10 takes place). For the wild type, the catalytic dwell state is sufficiently stable, this stability is accomplished by the nonuniformity of PEs of the three β subunits, the PE of each β subunit is intimately correlated with the chemical compound bound, and the orientation of the γ subunit strongly influences the state stability. Hence, when the orientation of the γ subunit is forcibly changed, the PEs of the three β subunits need to be changed, and to this end the chemical compounds bound to them are also changed. These features are lost for the mutant. The water-entropy loss accompanying (A)→(D) is much smaller than for the wild type, and the increase in system free energy due to this loss can be comparable in magnitude to the energy received by the system. The tight coupling between ATP hydrolysis or synthesis reaction and the rotation in the normal or inverse direction is no more attainable for the mutant.

## 8 Concluding remarks

The theoretical analyses performed for V_1_-ATPase have enabled us to obtain the following results.

(1) The packing structure of the catalytic dwell state illustrated in Figure 6B is constructed by the interplay of ATP bindings to two of the A subunits (A_TP_ and A_DP_) (more strictly, ATP-H_2_O is bound to A_DP_) and incorporation of the DF subunit. Replacing the two ATPs by two ADPs, respectively, lowers the packing efficiencies (PEs) of the three A subunits and markedly reduces the stability of the A_3_B_3_DF complex.
(2) The A_3_B_3_ complex is largely stabilized by the incorporation of the DF subunit. We can take the view that the orientation of the DF subunit follows the packing structure of the A_3_B_3_ complex thus largely stabilized (Section 7.2.2). There is the optimum orientation of the DF subunit for a packing structure of the A_3_B_3_ complex given, and even a slight shift from it gives rise to a significantly large loss of water entropy.
(3) Irrespective of the presence of nucleotides bound and the incorporation of the DF subunit, the PE of each subcomplex in V_1_-ATPase (Figure 5A) is determined by that of the A subunit. That is, Orders (9) and (10), (11) and (12), (13) and (14), and (15) and (16) hold for A_3_B_3_DF⋅2ATP, A_3_B_3_, A_3_B_3_⋅2ATP, and A_3_B_3_DF, respectively.
(4) The binding of ATP or ADP to an A subunit increases the affinity between this subunit and one of its neighbor B subunits, and thereby the ATP or ADP binding contributes to the formation of closer packing of the aforementioned A-B interface. The degree of the increase is much higher for the ATP binding. These results are in accord with the experimental observations reported by Murata and coworkers (Arai et al., 2020).
(5) Not only the packing structure of the A_3_B_3_DF complex but also the occurrence of ATP hydrolysis reaction in not A_TP_ but A_DP_ is highly relevant to the rotation of central shaft in the normal (i.e., counterclockwise) direction (Section 7.2.3).
(6) The packing efficiency (PE) of an A subunit (and that of the subcomplex in which it is included) is variable depending on the chemical compound bound to it. The packing structure where two nucleotides are bound to V_1_-ATPase is the most stable in terms of the water entropy. The most stable packing structure is characterized by the nonuniformity that the three A subunits are closely, moderately, and loosely packed, respectively, and the optimized orientation of the DF subunit. During each ATP hydrolysis cycle, when the chemical compound bound to one of the three A subunits changes in a direction that the PE of this A subunit becomes lower, the chemical compounds bound to the other two A subunits change in directions that their PEs become higher, thus maintaining the most stable packing structure (this view is supported by an experimental evidence (Kabaleeswaran et al., 2006): see Supplementary Section S15 for more details). Presumably, thanks to this maintenance, a significantly large free-energy barrier for the rotation of the DF subunit can be avoided. The ATP hydrolysis cycle and the maintenance of the most stable packing structure are in concert with each other (Section 7.3.1), accompanying a smooth rotation.
(7) It is possible that the incorporation of the central shaft in the A_3_B_3_ complex plays an essential role in the occurrence of ATP hydrolysis reaction in A_DP_.

The qualitative aspects of statements (1)−(6) should be applicable to F_1_-ATPase as well when “B”, “A”, and “DF” are replaced by “α”, “β”, and “γ”, respectively. [Statement (7) is not applicable to F_1_-ATPase as described below.] There are similarities and differences between F_1_-ATPase and V_1_-ATPase. The differences revealed in this study are summarized in Table 5. There can be another difference between the two rotary molecular motors. For F_1_-ATPase, since it was experimentally observed that the α_3_β_3_ complex fulfills its structural rotation in the normal direction (Uchihashi et al., 2011; Kinoshita, 2021a), the ATP hydrolysis should occur in β_DP_ even without the central shaft. For V_1_-ATPase to which statement (7) is applicable, on the other hand, it is possible that the rotation mechanism is programmed in not the A_3_B_3_ complex but the A_3_B_3_DF complex. Hence, the structural rotation of the A_3_B_3_ complex may not occur, pending experimental studies. As understood in statements (2), (5), and (7), the effect of incorporation of the central shaft on the structure and properties is considerably large in V_1_-ATPase, presumably larger than in F_1_-ATPase. Once the central shaft is incorporated, however, the two rotary molecular motors share essentially the same rotation mechanism despite the differences revealed (Figures 7, 8).

**TABLE 5:** Differences between F_1_-ATPase and V_1_-ATPase.

F_1_-ATPase	V_1_-ATPase
The structure of the α_3_β_3_ complex with no nucleotides bound is symmetric	The structure of the A_3_B_3_ complex with no nucleotides bound is asymmetric
In the catalytic dwell state shown in Figure 6A, Pi remains in β_E_ (Watanabe et al., 2010)	In the catalytic dwell state shown in Figure 6B, nothing remains in A_E_ (Iida et al., 2019)
In the catalytic dwell state, the packing efficiencies follow the orders, “subcomplex 3> subcomplex 2> subcomplex 1” and “β_DP_>β_TP_>β_E_”. See Figures 5E, 6A	In the catalytic dwell state, the packing efficiencies follow the orders, “subcomplex 2> subcomplex 3> subcomplex 1” and “A_TP_>A_DP_>A_E_”. See Figures 5E, 6B
After the ATP hydrolysis into ADP and Pi within β_DP_, ADP dissociates from it first (Watanabe et al., 2010)	After the ATP hydrolysis into ADP and Pi within A_DP_, Pi dissociates from it first (Iida et al., 2019)
The packing efficiency of a β subunit with a chemical compound bound follows Order (7)	The packing efficiency of an A subunit with a chemical compound bound follows Order (17)
In the catalytic dwell state, the γ subunit forms the most closely packed interface with β_DP_. See Figure 6A	In the catalytic dwell state, the DF subunit forms the most closely packed interface with A_E_. See Figure 6B

The system, which comprises not only F_1_-ATPase or V_1_-ATPase but also the aqueous solution of ATP, ADP, and Pi in which it is immersed, performs essentially no mechanical work during one ATP hydrolysis cycle. The central shaft is rotated by the torque generated by water with no input of chemical free energy of ATP. Even under the aqueous-solution condition that the overall reaction should be ATP hydrolysis, the central shaft rotates in the inverse direction when sufficiently strong external torque is imposed on it, performing the ATP synthesis (Toyabe et al., 2011; Toyabe and Muneyuki, 2015). This experimental evidence can also be explained as described in Section 7.3.4, which is an appealing point of our concept. The ATP hydrolysis or synthesis reaction is tightly coupled to the rotation in the normal or inverse direction through the water-entropy effect. Our proposal is in line with the recent experimental results observed for actomyosin (Section 1) and the theoretical efforts (Amano et al., 2010; Suzuki et al., 2017; Kinoshita, 2018) showing that the force for moving myosin unidirectionally along F-actin is generated through the effect of hydration of actomyosin (see Supplementary Section S16 and Section 2.9 in the recently published book (Kinoshita, 2021a)). In one of the theoretical efforts (Suzuki et al., 2017), the force acting on S1 was estimated to be several piconewtons, as strong as the experimentally observed force. The free energy of the system is variable depending on the conformation of actomyosin (i.e., structures, orientations, and positions of myosin and F-actin) or F_1_-ATPase. The conformation is perturbed by the ATP hydrolysis cycle but adjusted so that the system free energy can be lowered, which leads to the unidirectional movement or rotation.

There are two more reasons for which we request that the chemo-mechanical coupling be reconsidered:

Reason 1. Though the overall structures of F_1_-ATPase before and after the 120° rotation are the same, one ATP molecule has been hydrolyzed in bulk aqueous solution, accompanying a decrease in system free energy of Δ*G*_1_+Δ*G*_2_+Δ*G*_3_+Δ*G*_4_ = Δ*G*∼−12 kcal/mol (see Section 7.1.3 for the physical meanings of Δ*G*_1_ through Δ*G*_4_). Importantly, |Δ*G*_4_| is much smaller than | Δ*G* | (| Δ*G*_1_ | and | Δ*G*_3_ | can be relatively larger). In actomyosin, the free energy change upon the ATP hydrolysis in myosin (not in bulk aqueous solution), M⋅ATP+H_2_O→M⋅ADP-Pi (M denotes the myosin head), which corresponds to Δ*G*_4_<0, is only ∼−1 kcal/mol (Kodama, 1985). In the proposition that actomyosin is the system and the aqueous solution is the surroundings, the decrease in system free energy which can be transduced to mechanical work (the input of chemical free energy of ATP utilized by myosin) is neither Δ*G*∼−12 kcal/mol nor Δ*G*°∼−7 kcal/mol (Supplementary Section S3): It should be ∼ −1 kcal/mol which seems to be too small to perform the mechanical work for moving myosin unidirectionally. (In a study on F_1_-ATPase (Mukherjee and Warshel, 2015), the input of chemical free energy was unreasonably set at −8 kcal/mol though the aqueous solution was regarded as the surroundings.).

Reason 2. In the view based on the chemo-mechanical coupling, the chemical free energy of ATP needs to be input for the unidirectional movement or rotation. It is often necessitated to assume that the free energy is stored in actomyosin or F_1_-ATPase and utilized for the so-called mechanical event or mechanical events occurring afterwards. However, this assumption is not justifiable for the following reason. The state of actomyosin or F_1_-ATPase is driven to change so that the free energy of the system including the aqueous solution can become lowest. It was theoretically and experimentally shown that the relaxation of vibrational energy in a protein occurs on a time scale of picoseconds to nanoseconds at room temperature (Xie et al., 2001; Fujisaki and Straub, 2005). Likewise, it should be improbable that only actomyosin or F_1_-ATPase remains being in a state with higher free energy for a time scale of milliseconds on which the step movement or rotation takes place (Kitamura et al., 1999; Iwaki et al., 2018; Iida et al., 2019).

In the concept of chemo-mechanical coupling, the force or torque field generated by water acting on myosin or the central shaft (Section 7.3.1) is overlooked with the result that the chemical free energy of ATP is considered to be the only source of the force or torque generation.

Last, the subjects to be theoretically investigated in further studies are summarized in Supplementary Section S17.

## Data Availability

The raw data supporting the conclusion of this article will be made available by the authors, without undue reservation.
