# Supplementary material for: Physical pictures of rotation mechanisms of F1- and V1-ATPases: Leading roles of translational, configurational entropy of water
Source: Front Mol Biosci. 2023 Jun 9;10:1159603. doi: 10.3389/fmolb.2023.1159603 (PMC10288849; doi:10.3389/fmolb.2023.1159603)
Supplement: Supplementary file 2 [file DataSheet2.docx]

Supplementary Material

Physical pictures of rotation mechanisms of F_1_- and V_1_-ATPases: Leading roles of translational, configurational entropy of water

Satoshi Yasuda,^1,2,3^ Tomohiko Hayashi,^4,5^ Takeshi Murata,^1,2,3*^ and Masahiro Kinoshita^1,5,6*^

^1^Department of Chemistry, Graduate School of Science, Chiba University, Chiba, 265-8522, Japan

^2^Department of Quantum Life Science, Graduate School of Science, Chiba University, Chiba, 265-8522, Japan

^3^Membrane Protein Research and Molecular Chirality Research Centers, Chiba University, 1-33 Yayoi-cho, Inage, Chiba 263-8522, Japan

^4^Interdisciplinary Program of Biomedical Engineering, Assistive Technology, and Art and Sports Sciences, Faculty of Engineering, Niigata University, 8050 Ikarashi 2-no-cho, Nishi-ku, Niigata, 950-2181, Japan

^5^Institute of Advanced Energy, Kyoto University, Uji, Kyoto 611-0011, Japan

^6^Center for the Promotion of Interdisciplinary Education and Research, Kyoto University, Yoshida-honmachi, Sakyo-ku, Kyoto-shi, 606-8501, Japan

*Authors to whom correspondence should be addressed.

Tel: +81-43-290-2794; E-mail: t.murata@faculty.chiba-u.jp

Tel: +81-75-585-8372; E-mail: qq8d9fe9@wonder.ocn.ne.jp

# Imperative roles of translational, configurational entropy of water in biological self-assembly

## Decomposition of solute hydration into processes 1, 2-vdW, and 2-ES

The hydration of a solute (e.g., a protein) with a fixed structure comprises processes 1, 2-vdW, and 2-ES as illustrated in Figure S1.^1^ In process 1, the hydrophobic hydration, a cavity which geometrically matches the polyatomic structure of the solute is created in water. The solute can be modeled as a set of fused hard spheres. Process 1 is considered under the isochoric condition. This is because a biological self-assembly (e.g., receptor-ligand binding and protein folding) under the isobaric condition is accompanied by only a small change in system volume in the sense that the changes in hydration enthalpy and entropy are almost the same as those in hydration energy and entropy under the isochoric condition, respectively.^2−4^ (At 277 K, since the isobaric thermal expansion coefficient of water is zero, the changes in hydration enthalpy and entropy under the isobaric condition are exactly the same as those in hydration energy and entropy under the isochoric condition.^5^) In processes 2-vdW and 2-ES, solute-water van der Waals (vdW) and electrostatic (ES) potentials are incorporated, respectively. (In process 2-vdW, the solute atom-water hard sphere potential is replaced by the Lennard-Jones potential.)


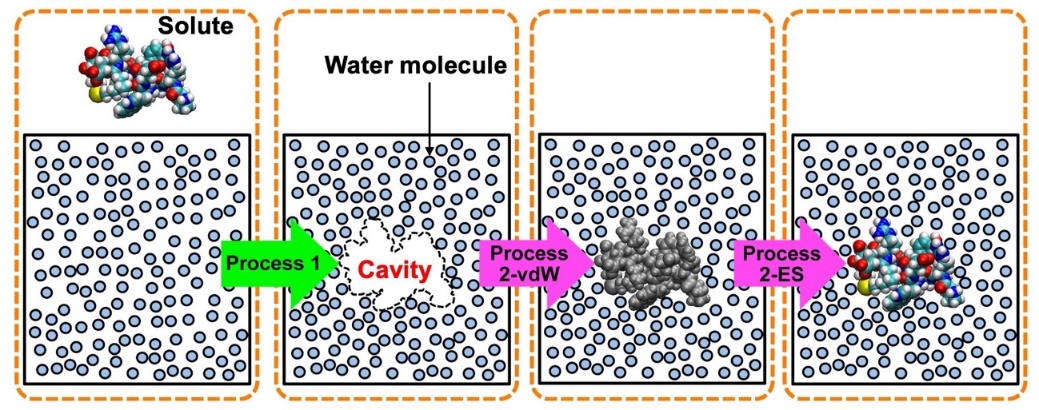


FIGURE S1

Decomposition of hydration of a solute with a fixed structure into processes 1, 2-vdW, and 2-ES.

A thermodynamic quantity of hydration in process 1 is defined as the change in free energy, energy, or entropy of water upon the cavity creation in a fixed position within water. For example, *S*_1_ is the loss of water entropy upon the cavity creation. A thermodynamic quantity of hydration in process 2-vdW or 2-ES is defined as the change in free energy, energy, or entropy of the system upon the incorporation of solute-water vdW or ES potential. The following equations hold:

*μ*_1_=*ε*_1_−*TS*_1_, (S1)

*μ*_2-vdW_­=*ε*_2-vdW_−*TS*_2-vdW_, (S2)

*μ*_2-ES_­=*ε*_2-ES_−*TS*_2-ES_, (S3)

*μ*=*ε*−*TS*, (S4)

*μ*=*μ*_1_+*μ*_2-vdW_+*μ*_2-ES_, (S5)

*S*=*S*_1_+*S*_2-vdW_+*S*_2-ES_, (S6)

*ε*=*ε*_1_+*ε*_2-vdW_+*ε*_2-ES_. (S7)

Here, *μ*, *ε*, and *S*, respectively, denote the hydration free energy, energy, and entropy, and the subscripts “1”, “2-vdW”, and “2-ES” signify processes 1, 2-vdW, and 2-ES, respectively. The hydration free energy *μ*=*μ*_1_+*μ*_2_, the free-energy change upon transfer of a solute with a fixed structure from vacuum to water (= free energy of water-solute system − (free energy of the solute + free energy of water)), can thus be decomposed into a variety of energetic and entropic components. We note that *ε*_1_, *S*_1_, *ε*_2-vdW_, *S*_2-vdW_, *ε*_2-ES_, and *S*_2-ES_ are all negative, and *μ*_1_ is positive whereas *μ*_2-vdW_ and *μ*_2-ES_ are negative. For solutes whose sizes are equal to or larger than the dipeptides, the following inequality holds:^1,3,4^

⎢*S*_1_⎢>>⎢*S*_2-vdW_⎢+⎢*S*_2-ES_⎢~⎢*S*_2-ES_⎢. (S8)

A dominant contributor to *S*_1_ is the loss of the translational, configurational entropy of water (the loss of the orientational entropy of water is much smaller^6^). ⎢*S*_1_⎢ and ⎢*ε*_1_⎢ become progressively larger as the excluded volume (EV) of the solute increases. When the solute is a protein, its EV is quite large. Consequently, the effect of the hydrophobic hydration dominates despite that polar and charged groups as well as nonpolar groups are exposed to water. Not only the solute but also a water molecule generates an EV for the other water molecules with the result that all the water molecules are entropically correlated. This entropic correlation is termed the “water crowding”. The conventional view of the hydrophobic effect focused on the water structure near a nonpolar group or surface should be reconsidered. ^3,4,7-14^ In most cases, the mitigation of water crowding in the entire system is more significant than the reduction in area of the nonpolar surface exposed to water in the hydrophobic effect.^3,4,8-13^

## Crucial importance of thermodynamic quantities of hydration relevant to process 1

The structures formed by biomolecules through the self-assembly are collapsed by the application of high pressures. Paradigmatic examples are the denaturation of a protein,^14^ dissociation of F-actin into actin monomers,^15,16^ and destruction of amyloid fibril,^17^ which take place upon pressure rise. The power of forming the structures becomes significantly weaker at low temperatures. Paradigmatic examples are the denaturation of a protein,^3,18^ weakening of the binding affinity of myosin for F-actin,^19^ and dissociation of protein aggregates,^20^ which occur upon temperature lowering. As relevant phenomena, at lowered temperatures, the solubility of methane in water becomes higher^6,21^ and the critical micelle concentration increases and the average size of micelles decreases for nonionic amphiphilic molecules.^22^ The native state of a protein is stabilized by addition of hydrophilic cosolvents (e.g., sugars^23^ and polyols^24^) or salts producing strongly hydrated ions (e.g., Na_2_SO_4_) but destabilized by addition of rather hydrophobic cosolvents (e.g., propanol) or salts producing weakly hydrated ions (e.g., (CH_3_)_4_NBr).^25^ In nonaqueous environments such as alcohol and nonpolar chains of lipid molecules of a membrane, only α-helices are formed as the secondary structures (i.e, β-sheets are formed only in aqueous environments).^26,27^

The experimentally known facts described above strongly suggest that water or biomolecule hydration is imperative in the self-assembly and a certain physical factor universally plays dominant roles as the driving force of the self-assembly. What is this physical factor? Kinoshita and coworkers showed that it is the biomolecule-water many-body (i.e., triplet and higher-order) correlation component of *S*_1_.^3,10,11,14,23-25^ The water crowding is the water-water pair and many-body correlations, and the mitigation of water crowding due to the self-assembly originates from the change in biomolecule-water many-body correlation component of *S*_1_. As mentioned above, the principal driving force in the hydrophobic effect is the mitigation of water crowding.

The temperature lowering leads to the reinforcement of hydrogen-bonding network of water molecules in the system. As a consequence, the translational motion of water molecules becomes less active, which is followed by the less significant water crowding and the weakened hydrophobic effect.^3^ At an elevated pressure, on the other hand, the hydrophobic effect is strengthened due to the higher number density of water molecules and the more significant water crowding. Interestingly, cold denaturation of a protein is caused by the weakening of hydrophobic effect,^3^ whereas the pressure denaturation is attributed to the severely strengthened hydrophobic effect.^14,28,29^ The structures of the pressure-denatured state,^14,28,29^ which are much more compact than those of the cold-denatured state,^30^ are referred to as the “swelling structures” characterized by the penetration of water molecules into the protein interior. This penetration mitigates the water crowding in the system, whereas the translational freedom of water molecules inside the protein is significantly more restricted. The water-entropy gain stemming from the former predominates over the water-entropy loss due to the latter at high pressures.^14,28,29^ As for the effects of cosolvents or salts, the native state of a protein becomes more stable when the effect of water crowding is enlarged by the cosolvent or salt addition.^23−25^

Taken together, *S*_1_ is the key quantity in arguing the biological self-assembly such as protein folding. All of the pressure and cold denaturations of a protein and the cosolvent and salt effects on the protein stability described above can be elucidated only by a theory where *S*_1_ is treated as a pivotal factor. The elucidation is possible even by a theory accounting for *S*_1_ alone.^9,23-25,28,29^ A protein comprises not only nonpolar residues but also polar and charged (both positively and negatively) residues. Nevertheless, its behavior is dominantly influenced by the hydrophobic hydration of process 1. For sodium ions, for example, their sizes are quite small for their net charges with the result that the thermodynamic quantities relevant to process 2 are much more influential than those relevant to process 1.

## Thermodynamics of protein folding

In this section, we give a more detailed description of the protein-folding mechanism elucidated by Kinoshita and coworkers^1,3^ using their recently developed statistical-mechanical method enabling us to accurately calculate *μ* of a protein with a moderate computational burden. By the method, *μ* can be decomposed into a variety of energetic and entropic components explained above. The Boltzmann-quasi-harmonic (BQH) method^4,14,31^ is the most accurate tool for estimating the protein intramolecular (conformational) entropy. We define the change in *X* (*X* is a thermodynamic quantity) upon protein folding, Δ*X*, as

Δ*X*=“*X* of folded state”−“*X* of unfolded state”. (S9)

Upon protein folding, the area of surface exposed to water becomes smaller. Much more protein intramolecular hydrogen bonds are formed but significantly many protein-water hydrogen bonds are broken. As a result, though Δ*E*_ES_ (*E*_ES_ is the protein intramolecular electrostatic energy) is negative (i.e., promoting the folding) and very large, Δ*ε*_2-ES_ is positive (i.e., opposing the folding) and very large. They are compensating, and Δ*E*_ES_+Δ*ε*_2-ES_ is positive and small. Protein folding is accompanied by the gain of protein intramolecular vdW interactions and the loss of protein-water vdW interactions: Though Δ*E*_vdW_ (*E*_vdW_ is the protein intramolecular vdW energy) is negative and large, Δ*ε*_2-vdW_ is positive and large. They are compensating, and Δ*E*_vdW_+Δ*ε*_2-vdW_ is negative and small. Δ*E*_ES_+Δ*ε*_2-ES_+Δ*E*_vdW_+Δ*ε*_2-vdW_ is positive and very small. Δ*S*_2-vdW_ can be neglected and Δ*S*_2-ES_>0 is significantly small. As a consequence, the thermodynamic quantities of hydration in process 1 dominate. Δ*ε*_1_ is positive and medium. The total-energy change, Δ*E*_ES_+Δ*ε*_2-ES_+Δ*E*_vdW_+Δ*ε*_2-vdW_+Δ*ε*_1_, is positive, implying that protein folding is entropy-driven. Upon protein folding, the EV generated by the protein becomes smaller. Therefore, Δ*S*_1_ (~Δ*S*) is positive and very large. Δ*S*_C_ (*S*_C_ is the conformational entropy) is negative and large, but Δ*S*_1_+Δ*S*_C_ is positive and significantly large. Moreover, ⎢−*T*Δ*S*_1_⎢>Δ*ε*_1_. Taken together, protein is driven to fold by the entropic component of the hydrophobic hydration, *S*_1_. It was shown in a study using a novel experimental technique which makes apoplastocyanin (a protein with 99 residues) fold at 298 K that protein folding is entropy-driven.^2^

A fact suggesting that the conventional view of the hydrophobic effect is not relevant is the following: By analyses on ultra-high resolution structures of 178 proteins, it was found that ~59% of the average water-accessible surface area is nonpolar, ~23% of it is polar, and ~19% of it is charged.^32^ For a protein which is heterogeneous in the sense that nonpolar, polar, and charged residues are disorderedly distributed in it, the formation of the so-called hydrophobic core is impossible,^7^ and the most important factor is to closely pack the backbone and side chains and reduce the EV as much as possible for mitigating the water crowding in the system.

It is true that *μ* of the unfolded state is lower than that of the folded state due to the dominance of Δ*ε*_2-ES_>0, Δ*ε*_2-vdW_>0, Δ*ε*_1_>0, and −*T*Δ*S*_C_>0.^1,3^ This fact has led several authors (see, for example, Ref. 33) to the wrong physical picture that water destabilizes the native state of a protein. The correct physical picture, which can be constructed only by decomposing *μ* into the variety of energetic and entropic components explained above, is that the native state of a protein is stabilized by the water-entropy effect (i.e., the entropic component of the hydrophobic hydration). The crucial importance of the water-entropy effect is reflected in the characteristics of the native state described in Subsec. 2.1. (The authors of Ref. 33 concluded that even when all the partial charges of protein atoms were set at zero and the backbone and side chains were made nonpolar, *μ* of the unfolded state was lower than that of the folded state. This conclusion, which must have arisen from an artifact of their method for calculating *μ*, is wrong. The result from our accurate method^1^ indicates that *μ* of the folded state is much lower than that of the unfolded state when all the partial charges of protein atoms are set at zero.)

## Thermodynamics of structural change of a protein complex

There is no reason why the thermodynamics of protein folding is not applicable to that of structural change of a protein complex. Let Δ*X* be the change in *X* upon the structural change. An argument which is similar to that for protein folding can be made. For example, if the number of hydrogen bonds within the complex increases, that of complex-water hydrogen bonds decreases. Δ*E*_ES_ and Δ*ε*_2-ES_, Δ*E*_vdW_ and Δ*ε*_2-vdW_, and Δ*E*_ES_+Δ*ε*_2-ES_ and Δ*E*_vdW_+Δ*ε*_2-vdW_ are all compensating. Furthermore, ⎪Δ*S*_1_⎪>⎪Δ*S*_C_⎪ and Δ*S*_1_ is dominant. It can be rationalized that the translational, configurational entropy of water is treated as a pivotal factor in the physical picture of unidirectional rotation of the γ subunit in F_1_-ATPase.

## Uniqueness of water as a solvent

There are a variety of solvents including water. For a solvent, its entropic effect becomes larger as its packing fraction increases (i.e., it becomes denser) and its molecular size becomes smaller.^7,10,12,34,35^ Due to the hydrogen bonds, water exists as a dense liquid at ambient temperature and pressure despite its exceptionally small molecular size. Water exhibits the largest entropic effect among the ordinary liquid solvents in nature.

Here, we refer to puzzling roles of water in the receptor-ligand binding. In usual binding processes, the binding interface is closely packed with the atomic-level shape complementarity, leading to a sufficiently large overlap of the EVs generated by the receptor and the ligand followed by the mitigation of water crowding: The binding is entropy-driven. However, there are exceptions. It is experimentally known that significantly many host-guest complexation processes are entropy-opposed (i.e., enthalpy-driven).^36,37^ It was found for a model cavity-ligand (both of them are nonpolar) binding by an MD simulation that it is entropy-opposed.^38^ Kinoshita and Hayashi^5^ showed that these results can also be explicated. The binding is entropy-opposed if the following two characteristics are found: The receptor and ligand surfaces which form the interface upon the binding are dominantly nonpolar; and a significant amount of space remains within the binding interface (i.e., a significant amount of space confined between the receptor and ligand surfaces remains) even after the binding is completed due to the lack of the shape complementarity. The water near each of the two dominantly nonpolar surfaces is enthalpically unstable because it can form only fewer hydrogen bonds than bulk water. For this reason, the number density of water near it becomes lower than that of bulk water. As the two surfaces approach each other, the enthalpic instability is enhanced and the number density of the confined water is made progressively lower. This is ascribed to the release of the water molecules forming only an insufficient number of hydrogen bonds to bulk water for recovering hydrogen bonds. Therefore, upon the binding, the total number of hydrogen bonds becomes significantly larger than in the case where the receptor and the ligand are well separated, leading to a decrease in enthalpy (factor A). However, the release of water molecules to bulk water increases the water crowding and the overlap of the EVs generated by the receptor and the ligand upon the binding is small due to the lack of the shape complementarity, causing a water-entropy loss (factor B). If gaining factor A is more important than avoiding factor B for lowering the system free energy, the binding is entropy-opposed (consult Ref. 5 for more details).

# Electrostatics in aqueous environment

It is often necessitated to determine which physical factors are more important than the others. This determination can be achieved only when an accurate model accounting for all the physical factors is employed and their relative magnitudes are quantitatively compared. On the basis of a series of studies along this line,^3,4,12,14,34^ we drew the conclusion that in aqueous environment the translational, configurational entropy of water originating from the EV effect is a pivotal physical factor. (Graziano and coworkers^32^ also suggested that the dominant force of protein folding is not the electrostatics but the EV effect.) Matubayasi and coworkers,^39,40^ using their accurate, versatile all-atom molecular dynamics (MD) simulation with explicit water, analyzed the energetics for Trp-cage (20 residues), protein G (56 residues), and ubiquitin (76 residues) in water at their native (folded) and heat-denatured (unfolded) states. The principal objective was to determine which of the electrostatic, van der Waals (vdW), and EV components of the interactions in the protein-water system governs the relative stabilities between the folded and unfolded structures. They found the following: The electrostatic interaction does not correspond to the preference order of the two structures; the EV and vdW components, on the other hand, provide the right order of preference at probabilities of almost unity; and a useful modeling of protein folding is possible on the basis of the EV effect. These results originate from, for example, the fact that an energy decrease due to a gain of electrostatic attractive interaction between portions of a protein (e.g., the formation of a protein intramolecular hydrogen bond) is always accompanied by an energy increase due to the penalty of electrostatic component of the energetic dehydration (e.g., the break of protein-water hydrogen bonds): The latter is often larger than the former,^12,34^ which can be reproduced only by a molecular model for water. The intermolecular electrostatic interaction for solutes immersed in aqueous solution is in effect much shorter-ranged and weaker than those in vacuum.^41^

It was claimed that the electrostatics predominates over the other physical factors in the rotation mechanism of F_1_-ATPase.^42^ On the other hand, according to experimental studies^43,44^ focused on electrostatic, attractive interaction between positively charged residues of Arg and Lys on the protruding portion of the γ subunit and negatively charged, acidic residues of Asp and Glu in one of the three β subunits, the rotation is not affected by mutating all the acidic residues to alanines. The authors of Ref. 42 claimed that even these experimental results (i.e., the effects of amino-acid mutations) could be reproduced by their theory emphasizing the dominance of electrostatics. However, the predominance of the electrostatics pointed out by them is not convincing because their theory suffers the following drawbacks. First, they represent a side chain in a protein as a simplified united atom which is spherical:^45^ The geometric properties of side chains, which are intimately related to the translational, configurational entropy of water and crucially important in the structure and properties of a protein,^10,12,26,27^ are completely lost; and the oversimplification of a side chain fails to account for the individuality of a residue. Second, a molecular model is not employed for water: The Coulomb potential between like- or unlike-charges is divided by an *effective* dielectric constant whose physical meaning is rather ambiguous. Third, the hydrophobic effect considered by them is based on the conventional, inappropriate method accounting for only the water structure near a nonpolar portion of a protein (see Subsec. S1.3) and the EV effect is neglected. Therefore, the translational, configurational entropy of water is not incorporated and even the electrostatics is not suitably taken into consideration.

Many of the biological self-assembly processes in aqueous environment are driven by not the intramolecular and intermolecular electrostatic attractive interactions of biomolecules but a gain of translational, configuration entropy of water. Some of the essential physical aspects of protein folding and denaturation can be captured using theoretical methods where only the water-entropy effect is taken into consideration.^2,10,11,28,29^ The electrostatics is important in the following respect. When charged groups are buried upon protein folding or protein-ligand binding, the contact of oppositely charged groups (i.e., gain of electrostatic attractive interaction between groups with positive and negative charges) are essential for compensating for the electrostatic energetic dehydration penalty arising from the loss of electrostatic attractive interactions between the group with a positive charge and water oxygens and between the group with a negative charge and water hydrogens. (Water oxygens and hydrogens carry negative and positive partial charges, respectively.) For the rotation of the central shaft in V_1_-ATPase, using all-atom MD simulations with explicit water, in which the translational, configuration entropy of water as well as the electrostatics is taken into account, Singharoy and coworkers^46,47^ showed that the electrostatic interactions at a specific residue-residue level and between a binding pocket and ATP, ADP+Pi, or ADP play important roles in the control of unidirectionality and rate of the rotation. During the rotation, it is essential to maintain the charge complementarity which was already achieved in F_1_- or V_1_-ATPase. For instance, the break of charge complementarity for a residue-residue pair must be compensated with the formation of charge complementarity for another residue-residue pair, and this is a significant factor in the rotation mechanism.

# Thermodynamics of ATP hydrolysis and synthesis reactions

We consider the chemical reaction, ATP+H_2_O→ADP+Pi (when the charge balance is emphasized, this is written as ATP^4−^+H_2_O→ADP^3−^+Pi^2−^+H^+^), in aqueous solution. The free-energy change upon this chemical reaction, *ΔG*, is expressed as the following approximate equation:

*ΔG*=*ΔG*°+*RT*ln([ADP][Pi]/[ATP]). (S10)

Here, Δ*G*°, *R*, and [*X*] denote the standard free energy change, gas constant, and dimensionless concentration of *X* (i.e., the concentration of *X* in mol/L divided by 1 mol/L), respectively. In the standard state, [ATP]=[ADP]=[Pi]=1. Δ*G*° is “free energy of aqueous solution containing 1 mol of ADP” + “free energy of aqueous solution containing 1 mol of Pi” − “free energy of aqueous solution containing 1 mol of ATP”. Δ*G*°~−12*k*_B_*T*~−7 kcal/mol at *T*=298 K (*k*_B_ is the Boltzmann constant and *T* is the absolute temperature set at 298 K).^48^ It is assumed that the chemical potential of water in aqueous solution is equal to that of pure water. A value of [ADP][Pi]/[ATP] can be validated only for given values of pH and [Mg^2+^].^49,50^

For Δ*G*<0, the reaction frequency in the normal direction (ATP hydrolysis: ATP+H_2_O→ADP+Pi) is much higher than that in the inverse direction (ATP synthesis: ATP+H_2_O←ADP+Pi). The overall reaction is ATP hydrolysis. Under the physiological condition, Δ*G*~−20*k*_B_*T*~−12 kcal/mol at *T*=298 K.^51^ For Δ*G*>0, the overall reaction is ATP synthesis. For Δ*G*=0, the state is in chemical equilibrium, and the reaction frequencies in the normal and inverse directions are the same (the reactions are not stopped). Let *C_X_* (mol/L) be the concentration of *X*. Δ*G*<0 is equivalent to ln([ADP][Pi]/[ATP])<−Δ*G*°/(*RT*), and Δ*G*<0 is true when *C*_ATP_ is sufficiently higher than *C*_ADP_ and *C*_Pi_. Δ*G*>0, which is equivalent to ln([ADP][Pi]/[ATP])>−Δ*G*°/(*RT*), is true when *C*_ATP_ is sufficiently lower than *C*_ADP_ and *C*_Pi_.

# Thermodynamics of ATP, ADP, or Pi binding to β or A subunit

We consider ATP, ADP, or Pi binding to a β subunit in F_1_-ATPae or an A subunit in V_1_-ATPase, U+Z→UZ where U denotes ATP, ADP, or Pi and Z denotes the β or A subunit. The free-energy change upon this binding, *ΔG*, is expressed as the following approximate equation:

*ΔG*=*ΔG*°+*RT*ln{[UZ]/([U][Z])}. (S11)

Here, Δ*G*° and [*X*] denote the standard free energy change and the dimensionless concentration of *X*, respectively. Δ*G*° is “free energy of aqueous solution containing 1 mol of UZ” − (“free energy of aqueous solution containing 1 mol of U” + “free energy of aqueous solution containing 1 mol of Z”).

For Δ*G*<0, the frequency of binding of U to Z (U+Z→UZ) is much higher than that of dissociation of U from Z (U+Z←UZ): Overall, the binding occurs. For Δ*G*>0, overall, the dissociation occurs. Δ*G*<0, for example, is equivalent to ln{[UZ]/([U][Z])}<−Δ*G*°/(*RT*), and Δ*G*<0 is true when the concentration of U is sufficiently high. The results shown in Table V suggest the following: Δ*G*°<0 for U=ATP or ADP. Nevertheless, ln{[UZ]/([U][Z])}>−Δ*G*°/(*RT*) (i.e., Δ*G*>0) is true when the concentration of U (ATP or ADP) is sufficiently low, leading to the dissociation of U.

# Nucleotide bindings to two of β subunits

In the state stabilized in aqueous solution of nucleotides, two nucleotides are bound to two of the three β subunits, respectively (Figure 2). AMP-PNP is an analogue of ATP and not hydrolyzed. AMP-PNP, which can thus be regarded as ATP whose hydrolysis does not occur, is used when the solution of a crystal structure is undertaken. States where the number of nucleotides bound to the three β subunits is zero, one, and three should be less stable. In aqueous solution of AMP-PNP and ADP, two AMP-PNPs are bound to two of the three β subunits, respectively, and the binding of ADP does not occur unless azide is added to the aqueous solution (only in the presence of azide, AMP-PNP and ADP are bound to β_TP_ and β_DP_, respectively).^52^ Thus, the state with two AMP-PNPs bound to the three β subunits is more stable than the states with two ADPs bound and with AMP-PNP and ADP bound. (Hereafter, unless otherwise mentioned, ATP and AMP-PNP are not distinguished.) Importantly, it was observed that β_DP_, β_TP_, and β_E_ take closed, closed, and open structures, respectively.^52^ Namely, the backbone and side chains in a β subunit with a nucleotide bound is more closely packed than those in a β subunit with no nucleotides bound.

A question arises: Why does ATP not bind to β_E_ when the α_3_β_3_γ complex is in the state where two ATPs are bound to β_DP_ and β_TP_, respectively? In our view, when the two binding processes, “α_3_β_3_γ+3ATP→α_3_β_3_γ**⋅**2ATP+ATP” and “α_3_β_3_γ+3ATP→α_3_β_3_γ**⋅**3ATP”, are compared, the water-entropy gain upon the latter is much smaller than that upon the former: It is not large enough to make the free-energy change negative. Here, α_3_β_3_γ**⋅**2ATP, for example, denotes the α_3_β_3_γ complex with two ATPs bound. It was previously shown by Kinoshita and coworkers^53,54^ that in a protein complex the close packing of all the protein interfaces is particularly important in terms of the water entropy. If ATP bound to β_E_, the structure of β_E_ would be modified to the closed one. However, it would become impossible to realize the close packing of all the α-β interfaces with all the three β subunits taking the closed structures. Thus, the ATP binding to β_E_, which causes a decrease of the water entropy, is not allowed.

# Crystal structures for V_1_-ATPase

In addition to the crystal structures described in Subsec. 5.1, a crystal structure was also reported for the A_3_B_3_DF complex with two ADP·AlF_4_s (7VW7 and *R*=3.8 Å) are bound.^47^ ADP·AlF_4_ can be regarded as ATP(ATP-H_2_O) or ATP-H_2_O.^55,56^ However, since its resolution exceeding 3.5 Å may be unacceptably low, it will be used in our future study after the refinement using all-atom MD simulation. According to the experimental studies of Murata and coworkers,^57^ three nucleotides can hardly bind to V_1_-ATPase. In aqueous solution of ADP, the number of ADPs bound was two in an ADP concentration of 20 μM. It became three only when the ADP concentration was increased to quite a high value (e.g., 2 mM). In aqueous solution of AMP-PNP, the number of AMP-PNPs bound was two even when the AMP-PNP concentration was made quite high.

# Angle-dependent integral equation theory and morphometric approach

In the angle-dependent integral equation theory (ADIET),^6,58,59^ the potential between two water molecules is dependent on their orientations represented by the Euler angles. The potential between an atom in the solute and a water molecule is also dependent on the orientation of the water molecule. A water molecule is modeled as a hard sphere with a diameter of 0.28 nm in which a point dipole and a point quadrupole of tetrahedral symmetry are embedded.^58,60^ The influence of molecular polarizability of water is included by employing the self-consistent mean field (SCMF) theory.^58,60^ At the SCMF level the many-body induced interactions are reduced to pairwise additive potentials involving an effective dipole moment. The effective dipole moment thus determined is about 1.42 times larger than the bare gas-phase dipole moment. The correlations are functions of multiple variables. The resultant mathematical complexity emerging in the case where the solute possesses polyatomic structure is avoided by combining the ADIET with the MA.

In the MA, *S*_1_ is expressed as a linear combination of the four geometric measures of the cavity, the EV, water-accessible surface area, and integrated mean and Gaussian curvatures of the water-accessible surface.^11,27,61^ We refer to this linear combination as the “morphometric form”. The four coefficients in the morphometric form, which are independent of geometric characteristics of the cavity, are determined beforehand for spherical cavities with sufficiently many different diameters using the ADIET. Since the ADIET is employed, not only the translational contribution but also the orientational one is included in *S*_1_ (Subsec. S1.1)^6,58−60^ though the latter is much smaller.

For a solute structure given, which is modeled as fused hard spheres (a hard sphere corresponds to an atom in the solute), the four geometric measures are calculated from the *x*-, *y*-, and *z*-coordinates and diameters of all the hard-sphere atoms constituting the solute. The diameter of each atom is set at the *σ*-value in the Lennard-Jones potential parameters which are taken from CHARMM22.^62^ *S*_1_ of a solute is obtained directly from the morphometric form. The high accuracy of the ADIET-MA method was corroborated in our earlier works.^11,27^

# Differences between previous studies by Murata and coworkers and this study in naming A-B subunit pairs in V_1_-ATPase

In Table S1, we summarize the differences between previous studies by Murata and coworkers^57,63^ and this study in naming the A-B subunit pairs.

TABLE S1 Differences between previous studies by Murata and coworkers^57,63^ and this study in naming A-B subunit pairs in V_1_-ATPase. Murata and coworkers viewed V_1_-ATPase from the top of the A_3_B_3_DF complex: The DF subunit rotates in the clockwise direction.

| State | Previous studies | This study |
| --- | --- | --- |
| A_3_B_3_DF complex with no nucleotides bound  (PDB Code: 3VR4)^63^ | Empty | A_E_-B_E_ |
|  | Bound | A_TP_-B_TP_ |
|  | Tight | A_DP_-B_DP_ |
| A_3_B_3_DF complex with two AMP-PNPs bound  (PDB Code: 3VR6)^63^ | Empty | A_E_-B_E_ |
|  | Bound | A_TP_-B_TP_ |
|  | Tight | A_DP_-B_DP_ |
| A_3_B_3_DF complex with two ADPs bound  (PDB Code: 5KNB)^57^ | Bindable-like | A_E_-B_E_ |
|  | Bound | A_TP_-B_TP_ |
|  | ADP-bound | A_DP_-B_DP_ |
| A_3_B_3_ complex with no nucleotides bound  (PDB Code: 3VR2)^63^ | Empty | A_E_-B_E_ |
|  | Bindable | A_TP_-B_TP_ |
|  | Bound | A_DP_-B_DP_ |
| A_3_B_3_ complex with two AMP-PNPs bound  (PDB Code: 3VR3)^63^ | Empty | A_E_-B_E_ |
|  | Bound | A_TP_-B_TP_ |
|  | Bound | A_DP_-B_DP_ |

# Reliability of water-entropy gain upon binding of two subunits calculated

The small numbers of water-entropy gains upon binding of two subunits of V_1_-ATPase (e.g., those in Figure 5) are calculated as the differences between large numbers of the hydration entropies. However, this causes no problems for the following reasons:

(1) The water-entropy gain upon binding of two subunits is calculated using Eq. (8) in which the two subunits are taken from a complex and the two subunits before binding are obtained by simply separating them. The hydration entropy originates from two portions of each subunit, a portion which is buried in the binding interface (portion 1) and the remaining portion which is not (portion 2). Only the hydration entropy relevant to portion 2 is large and that relevant to portion 1 is much smaller. The hydration entropy of a subunit is large just because that relevant to portion 2 is large. Importantly, the hydration entropies relevant to portions 2 of two subunits are cancelled out when the difference is taken. Thus, the difference is obtained, in essence, as that between the much smaller numbers for portions 1 of two subunits and should be free from large errors. In the calculation, we do not take account of the structural changes of two subunits upon the aforementioned separation and the structure fluctuations. However, we note that the quantitatively high accuracy is not crucial in our discussions. The crucial points are the following: The packing structures of the A_3_B_3_ complex in V_1_-ATPases are not uniform and, for example, the packings of the three A subunits are relatively close, moderate, and loose, respectively, and some of the binding interfaces are more closely packed than the others; and the orientation of the DF subunit is changed in response to the change in packing structure of the A_3_B_3_ complex. See the discussions on Figures 6 through 10. (We would mention that the numbers of water-entropy gains upon binding of two subunits are significantly large; see Sec. S10.)

(2) The statistical-mechanical calculations are performed using the crystal structures of V_1_-ATPase experimentally determined. Only one structural data is registered for each structure in the Protein Data Bank.^57,63^ Hence, we cannot estimate the error bars, but the estimation is not important for the reason described in (1).

(3) As a matter of fact, we calculated binding free energies for an oncoprotein MDM2 and three different peptides^4^ using the same method for calculating the water-entropy gain upon binding. All of the hydration entropies and energies and the intramolecular (conformational) entropies and energies of the protein-peptide complex, protein, and peptide were incorporated in the calculation. Moreover, the structural fluctuations of the protein-peptide complex, protein, and peptide were taken into account. Nevertheless, it was shown that the difference in terms of the binding free energy between any two of the three peptides calculated are in *quantitatively* good accord with the experimentally measured values.^4^ Thus, the reliability of our method for calculating the water-entropy gain upon binding has already been corroborated.

# Packing of atoms within binding interface of protein-protein complexes

It is true that the packing of atoms within the binding interface of a protein-protein or protein-ligand complex can be loose. We discuss such a case where the binding becomes enthalpy-driven in Subsec. S1.5. However, it has been suggested that there is also a case where protein side chains within the binding interface are significantly flexible, possibly more flexible than those in an isolated protein, which may lead to an increase in the conformational entropy of side chains and drive the binding.^64,65^ We note, however, that a sufficiently accurate theoretical tool is required for the corroboration of such an increase: The corroboration has not been achieved yet.

Importantly, the case where the conformational entropy of side chains may increase upon binding is not applicable to F_1_- and V_1_-ATPases for the following reason:

A large gain of water entropy upon binding occurs only when the atoms in the binding interface are closely packed (i.e., the shape complementarity at the atomic level is achieved in the binding interface). The close packing leads to a large decrease in the total excluded volume, a large increase in the total volume available for the translational displacement of water molecules in the entire system, and a large gain of water entropy. In the case where the total conformational entropy may increase, a large gain of water entropy does not occur. The significantly large water-entropy gains upon binding of two subunits of V_1_-ATPase given in Table 3 (even the smallest number is 368.5*k*_B_), which are calculated using the crystal structures of V_1_-ATPase experimentally determined,^57,63^ manifests the close packing of the atoms within the binding interface. The same can be mentioned for F_1_-ATPase considered in our earlier work.^53^ Even when we mention that the packing of the atoms within a binding interface is loose for F_1_- or V_1_-ATPase, we mean that it is *only relatively looser*. The close packing of the protein atoms in the A_3_B_3_DF complex (V_1_-ATPase) with two AMP-PNPs bound (PDB Code: 3VR6)^63^ is illustrated in Figure S2.

Using all-atom MD simulations with explicit water for binding of an oncoprotein MDM2 and three different peptides^4^ and actin-myosin binding,^66^ we verified that the side chains buried within the binding interface of the complex undergo freezing of their structural flexibilities and large water-entropy gains occur upon binding. (We analyzed a number of the snapshot structures of isolated MDM2, peptides, actin, and myosin as well as the protein-peptide and protein-protein complexes obtained during the MD simulations.) In the case of the MDM2-peptide binding, we estimated the change in the total conformational　entropy upon binding by employing the BQH method.^4,14,31^ The change is certainly negative, indicating that the binding is accompanied by a large loss of the total conformational entropy. The estimated value was incorporated in the binding free energy calculated (see (3) in Sec. S9). Thus, the binding process is usually accompanied by a significantly large loss of the total conformational entropy. This is particularly true in protein-ligand binding in which the ligand undergoes a large loss of its conformational entropy because the ligand does not take a well-defined structure when it is isolated.

We note that it is not an easy task to calculate the change in the conformational entropy of protein side chains as well as the change in the total conformational entropy upon protein-ligand or protein-protein binding with sufficient accuracy even with the BQH method^31^ combined with all-atom MD simulations: We have found that the calculation result suffers a large error unless special cares are taken (unpublished results). We are writing a paper reporting this which is intended for publication. We emphasize that the special cares were taken in our calculations^4^ referred to above. Furthermore, as pointed out by McCammon and coworkers,^67^ upon binding, the volume available for the translational displacement of the ligand decreases from 1 L/mol to the volume of the binding region and the rotational freedom of the ligand is more restricted. This effect, which leads to a decrease in the entropy of the ligand, is also to be taken into consideration. As a future study, it is interesting to check if there is a case where the conformational entropy of protein side chains or the total entropy of the protein-protein or protein-ligand pair actually increases upon binding (and this increase works as a driving force) by employing the BQH method with the special cares mentioned above and correcting the change in total entropy in compliance with the suggestion by McCammon and coworkers.


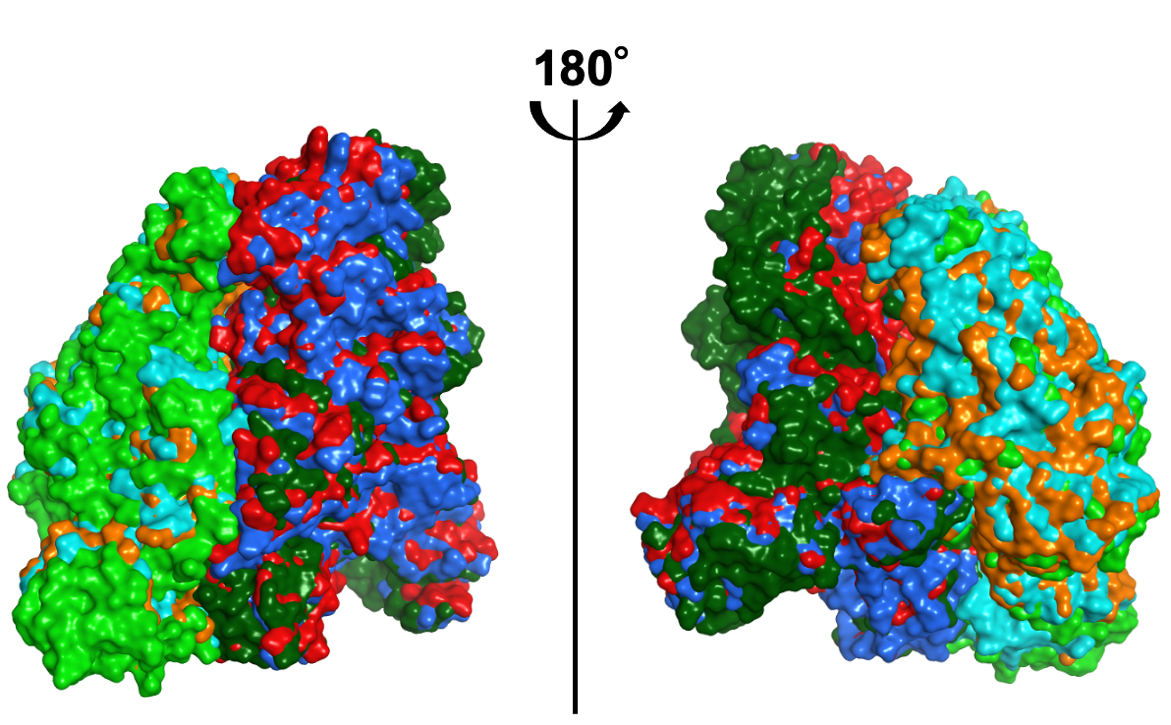


FIGURE S2

Visualization of close packing of protein atoms in the A_3_B_3_DF complex (V_1_-ATPase) with two AMP-PNPs bound (PDB Code: 3VR6).^63^ The right structure is obtained by rotating the left one by 180°. The atoms in A_E_, B_E_, A_TP_, B_TP_, A_DP_, and B_DP_ are colored dark green, yellow green, red, orange, blue, and light blue, respectively. The atoms of AMP-PNPs are buried in the interior and cannot be seen.

# Energetics for F_1_-ATPase

The authors of Ref. 68, in its Supporting Information, criticized the energetics for F_1_-ATPase published in our earlier paper.^53^ They stated that our theory did not reproduce the correct energetics because it produced around 400 kcal/mol for the steps considered instead of around 10−15 kcal/mol, making the discussion rather irrelevant. This statement is wrong. We wonder where “400 kcal/mol” came from. It is probable that they refer to such a large number in one of the Tables in Ref. 53 and, for example, in Figure 5 of this study: They misunderstand the physical meanings of the numbers given. We discuss the energetics in Subsec. 7.1.3 for excluding such a misunderstanding. The free-energy changes in state changes (A)→(B) and (B)→(C) are smaller than −20*k*_B_*T*~−12 kcal/mol (*T*=298 K): Upon state change (A)→(C), the free energy becomes lower by ~−12 kcal/mol.

# Occurrence of ATP hydrolysis reaction in not ATP but ADP

In connection with the discussion in Subsec. 7.2.3, we present the following two figures.


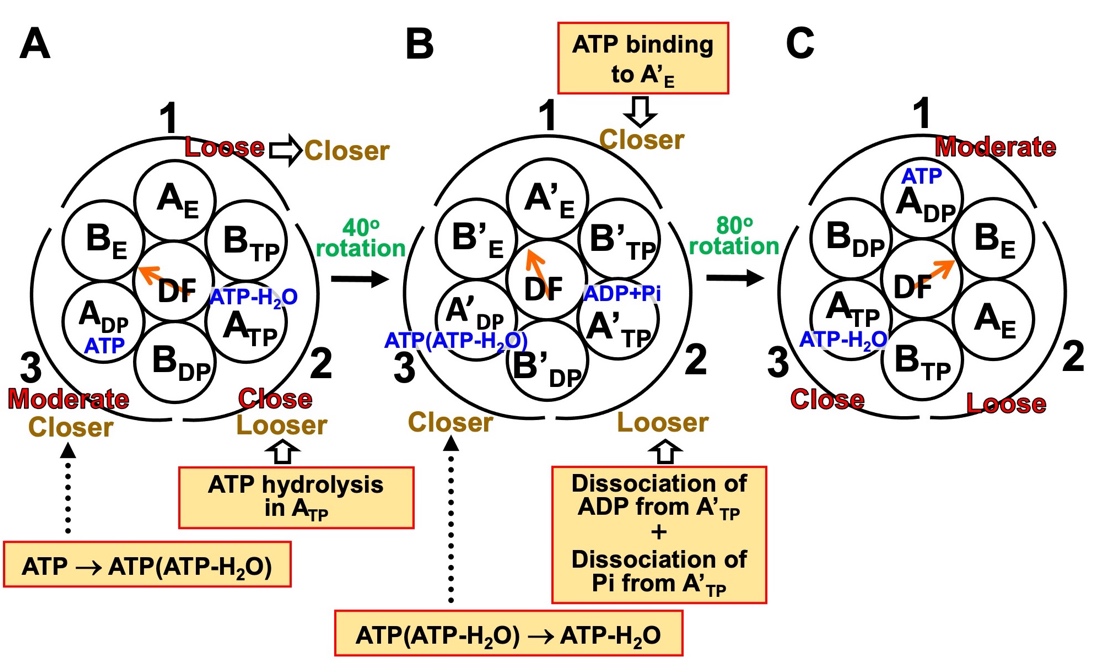


FIGURE S3

Rotation of DF subunit in inverse (i.e., clockwise) direction during one ATP hydrolysis cycle. It is assumed that ATP, ATP-H_2_O, and nothing are bound to A_DP_, A_TP_, and A_E_, respectively, the ATP hydrolysis occurs in ATP in step (1), and Order (7) with “β” being replaced by “A” is followed. See the caption of Figure 8 as well.


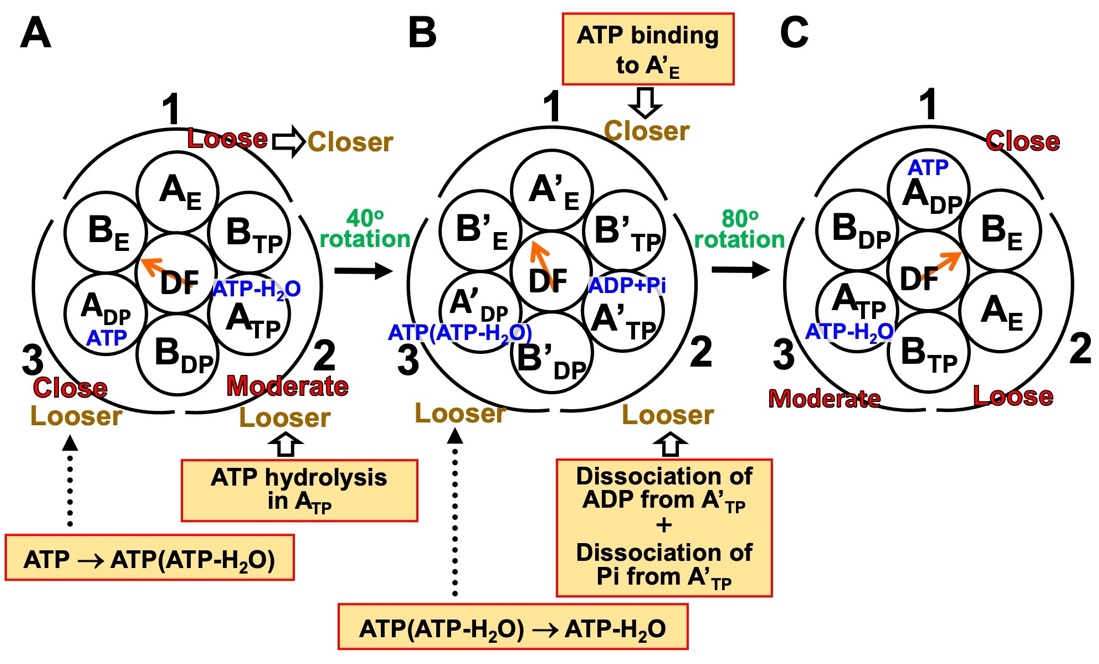


FIGURE S4

Rotation of DF subunit in inverse (i.e., clockwise) direction during one ATP hydrolysis cycle. It is assumed that ATP, ATP-H_2_O, and nothing are bound to A_DP_, A_TP_, and A_E_, respectively, the ATP hydrolysis occurs in ATP in step (1), and Order (17) is followed. See the caption of Figure 8 as well.

# Mechanism of force or torque generation for moving myosin along F-actin or rotating central shaft of F_1_-ATPase

First, we consider F-actin and a myosin head (e.g., myosin subfragment 1 (S1)).^69^ We assume that the position and structure of F-actin are fixed. It should be noted that the system comprises not only F-actin and the myosin head but also water (the aqueous solution is referred to simply as water when the water roles are emphasized) in which they are immersed. The free energy of the system is denoted by *G*_System_. (Since a structural change of a protein and a protein-ligand binding occur with the system volume being almost unchanged even under the isobaric condition,^2−4^ we use “free energy” instead of “Gibbs free energy” or “Helmholtz free energy”.) The structure and position of the myosin head change in the direction of lowering *G*_System_. For a prescribed structure of the myosin head, *G*_System_ is a function of the position of the myosin head which can be represented by the Cartesian coordinates of the center of gravity of the myosin head, (*x*, *y*, *z*). The origin of the coordinate system (0, 0, 0) is chosen to be, for example, the left edge of F-actin. The *x*-axis is taken to be in the direction of forward movement of the myosin head along F-actin. *Φ*(*x*, *y*, *z*) defined as

*Φ*(*x*, *y*, *z*)=*G*_System_(*x*, *y*, *z*)−*G*_System_(+∞, +∞, +∞) (S12)

represents the spatial distribution of the potential of mean force (PMF), the water-mediated potential, between F-actin and the myosin head.^34,70^ The mean force (MF) acting on the myosin head, ***f***, is expressed as

***f***=*f_x_***i**+*f_y_***j**+*f_z_***k**, *f_x_*= −∂*Φ*/∂*x*, *f_y_*= −∂*Φ*/∂*y*, *f_z_*= −∂*Φ*/∂*z* (S13)

where **i**, **j**, and **k** denote the direction unit vectors. ***f***(*x*_0_, *y*_0_, *z*_0_) represents the force induced between F-actin and the myosin head averaged over all the possible configurations of water molecules in the entire system with (*x*, *y*, *z*) being fixed at (*x*_0_, *y*_0_, *z*_0_). The concept of PMF or MF is based on the assumption that water is always in equilibrium with each configuration of F-actin and the myosin head. This assumption is justifiable because in the real system the hydration structure of a protein steadies in picoseconds,^71^ while the movement of the myosin head occurs in milliseconds.^69,72^ Thus, a potential or force field acts on the myosin head due to the presence of F-actin near it. If the myosin head was isolated, its motion would be random: It would make a Brownian motion in the absence of F-actin. When the myosin head is near F-actin, however, its motion is influenced by the potential or force field. For example, it tends to be moved in the direction of lowering the potential but it cannot overcome a potential barrier which is much higher than *k*_B_*T*.

Importantly, the potential or force field is strongly dependent on the structure of the myosin head which is variable depending on the chemical compound (nothing, ATP, ADP+Pi, or ADP) bound to the myosin head. Upon a structural change of the myosin head, *Φ*(*x*, *y*, *z*) and ***f***(*x*, *y*, *z*) also exhibit significantly large changes. That is, the potential or force field acting on the myosin head exhibits sequential changes during the ATP hydrolysis cycle (binding of ATP to the myosin head, hydrolysis of ATP into ADP and Pi in it, release of Pi from it, and release of ADP from it) which can lead to the unidirectional movement of the myosin head along F-actin. (In each cycle, *G*_System_ decreases by the free-energy change upon the ATP hydrolysis in bulk aqueous solution, Δ*G*~−20*k*_B_*T*~−12 kcal/mol at *T*=298 K.).

In what follows, we recapitulate our preliminary physical picture of the unidirectional movement of myosin subfragment 1 (S1) along F-actin, which was constructed using simplified models for S1 and F-actin.^73,74^ In the experiment by Kitamura et al.,^69^ S1 is constrained on F-actin using a novel technique, and it moves only along the *x*-axis (Figure S5A). The PMF between F-actin and S1, *Φ*(*x*), calculated is shown in Figure S5B. The black curve represents the PMF for S1 with nothing or ADP bound and the red curve represents the PMF for S1 with ATP or ADP+Pi bound. The two curves share the same periodicity, ~5.3 nm, that is only slightly smaller than the size of G-actin, ~5.5 nm. Initially, the black curve acts on S1 with nothing bound. S1 is stabilized at the position where the potential takes a local minimum value due to a potential barrier which cannot be overcome. Upon binding of ATP to S1, the PMF acting on S1 switches from the black curve to the red one. After the ATP hydrolysis reaction is completed in S1, upon release of Pi from S1, the PMF acting on S1 switches from the red curve to the black one. S1 then moves in the forward direction by a single step of ~5.5 nm as indicated by the blue broken line (Figure S5B). Since the potential barriers in the red curve are not significant higher than *k*_B_*T*, S1 can move in the forward direction by two or more steps. It often returns to the same position or sometimes moves in the backward direction. These results are in good accord with the experimental observations.^69^ In a strict sense, the structure of S1 exhibits not a step change but a gradual one. When the effect of this gradual change is taken into account, since the structure of S1 changes so that *G*_System_ can always be lowered, the red curve gradually shifts in the negative direction as S1 moves and its structural change proceeds (this is not taken into account in Figure S5B). The effect of the gradual change can enhance the unidirectionality in the biased Brownian motion in the presence of the red curve. Refining the physical picture or extending it to the unidirectional movement of myosin V, a double-headed myosin, along F-actin is to be considered in future studies.


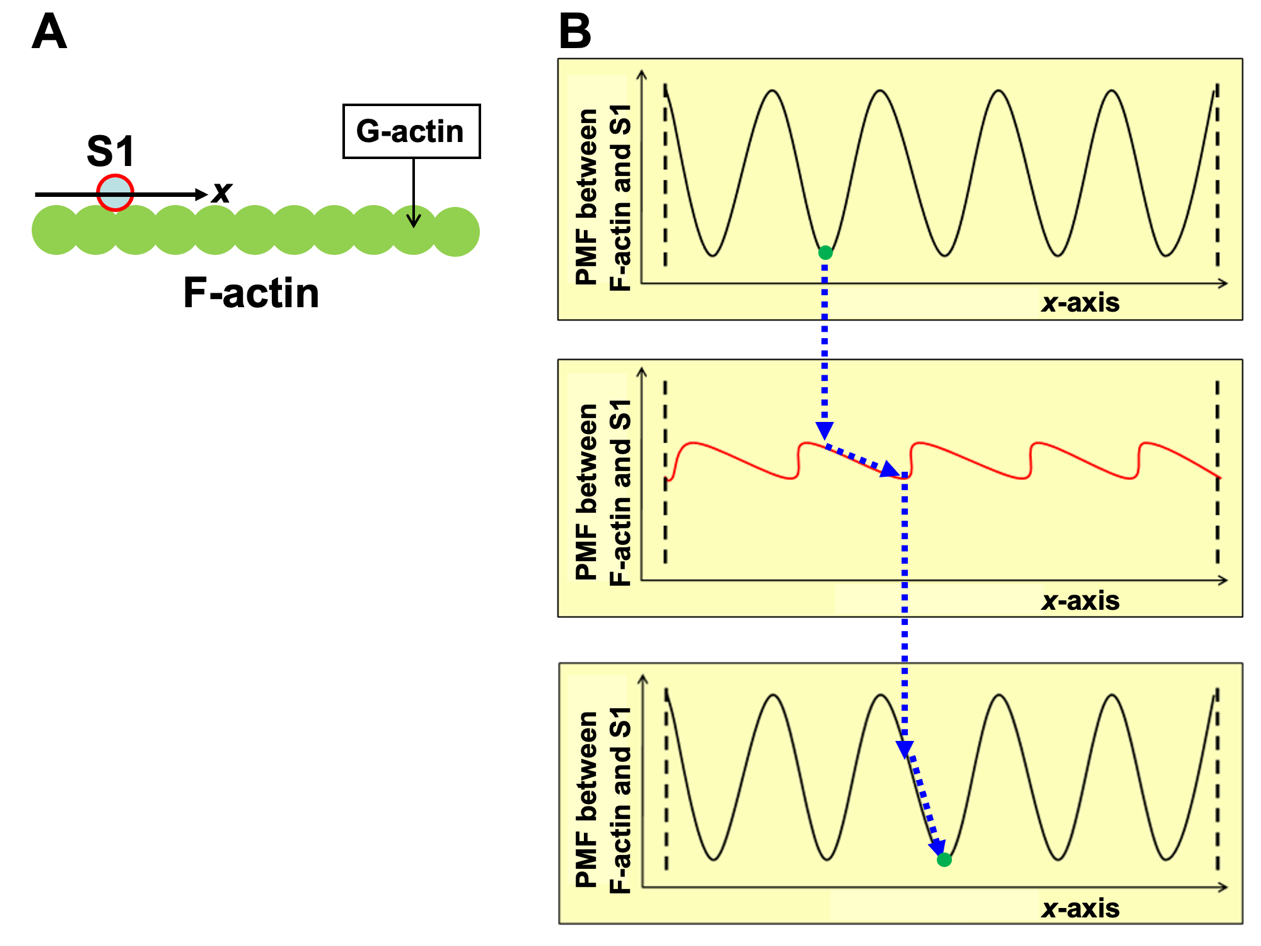


FIGURE S5

(A) Movement of S1 along F-actin. (B) Movement of S1 by a single step in the forward direction. In the black curve, S1 is stabilized at the position indicated by the green closed circle.

Second, we consider F_1_-ATPase. The free energy of the system is denoted by *G*_System_. The system comprises not only F_1_-ATPase but also water. For a structure of the α_3_β_3_ complex in F_1_-ATPase given, *G*_System_ is a function of the rotation angle *θ* defined for the γ subunit in the counterclockwise direction with the origin suitably chosen. The structure of the γ subunit is also variable depending on *θ*. The mean torque acting on the γ subunit, *τ*(*θ*), can be expresses as

*τ*= −∂*G*_System_/∂*θ*. (S14)

*τ*(*θ*_0_) represents the torque acting on the γ subunit averaged over all the possible configurations of water molecules in the entire system with *θ* being fixed at *θ*_0_. In this case, *G*_System_ takes a sharp minimum at *θ*=*θ*_min_ and *τ*(*θ*_min_)=0. Since *G*_System_ is strongly dependent on *θ*, the torque expressed by Eq. (S14) is also strong. During the ATP hydrolysis cycle, the chemical compounds bound to the three β subunits, the structure of the α_3_β_3_ complex, and *τ*(*θ*) and *θ*_min_ exhibit sequential changes. This leads to the unidirectional rotation of the γ subunit.

*G*_System_ is expresses as

*G*_System_=*E*_C_−*TS*_C_+*μ*, *μ*=*ε*−*TS*, *G*_System_=*E*_C_+*ε*−*TS*_C_−*TS* (S15)

where *E*_C_ is the intramolecular (conformational) energy, *S*_C_ is the conformational entropy, and *μ*, *ε*, and *S* are the hydration free energy, energy, and entropy, respectively. *E*_C_, *S*_C_, *μ*, *ε*, and *S* are defined for the combination of F-actin and the myosin head or that of the α_3_β_3_ complex and the γ subunit. ⎪*S*⎪ of a solute denotes the magnitude of water-entropy loss caused by the solute insertion. In general, a change in conformational energy *ΔE*_C_ and that in hydration energy Δ*ε* are compensating.^3,4^ (*ΔX* signifies the change in *X* upon a structural change of a protein. In the present case, it signifies the change in *X* caused by structural and positional changes of the myosin head or by structural changes of the α_3_β_3_ complex and the γ subunit and an orientational change of the γ subunit.) That is, *Δε*>0 for *ΔE*_C_<0 and *Δε*<0 for *ΔE*_C_>0, and *ΔE*_C_+*Δε* is usually positive but much smaller than ⎪−*TΔS*⎪. When two oppositely charged groups of the protein are buried and contacted, for example, *E*_C_ decreases due to a gain of electrostatic attractive interaction between the two groups. However, the gain accompanies a loss of group-water electrostatic attractive interactions, causing an increase in *ε*. This is why *ΔE*_C_ and Δ*ε* are compensating. On the other hand, ⎪*ΔS*_C_⎪ is significantly smaller than ⎪*ΔS*⎪.^3,4^ Therefore, when we discuss *ΔG*_System_, the water entropy *S*_Water_ can be regarded as a principal component of *G*_System_:

*ΔG*_System_~−*TΔS*_Water_. (S16)

We can take the view that the structure and position of the myosin head or the structure of the α_3_β_3_ complex and the structure and orientation of the γ subunit are determined so that the water entropy can be increased. Thus, *G*_System_ in Eqs. (S12) and (S14) can be replaced by −*TΔS*_Water_. *τ*(*θ*) can be referred to as the “entropic torque”.

It is now apparent that the force moving the myosin head and the torque rotating the central shaft are generated by water in the physical pictures explained above. No input of chemical free energy of ATP is required for the force or torque generation. It is just that in an ATP hydrolysis cycle the system free energy decreases by the free-energy change upon the ATP hydrolysis in bulk aqueous solution. We note, however, that this never means that ATP plays no significant roles. In the absence of ATP, the unidirectionality of the movement or rotation is not achievable: Once the myosin head with a prescribed structure, for example, is stabilized in the position where *G*_System_ is minimized, its movement is stopped unless it can overcome the free-energy barrier because the potential or force field is not changed. ATP certainly works as a trigger of the change in the potential or force field. For actomyosin, the extension of the physical picture to the unidirectional movement of myosin V along F-actin is to be considered in future studies. In any case, the conformation of actomyosin (i.e., structures, orientations, and positions of myosin and F-actin) or that of F_1_-ATPase is perturbed by the ATP hydrolysis cycle (Subsec. 3.1) but adjusted so that the system free energy can be lowered, which leads to the unidirectional movement or rotation.

We remark that the concept of chemo-mechanical coupling must have arisen from the overlooking of the force or torque field generated by water acting on myosin or the central shaft of F_1_- or V_1_-ATPase. In the chemo-mechanical coupling, the chemical free energy of ATP was incorrectly considered to be the only source of the force or torque generation.

# Relation among energy given to the system, energy lost as heat, and energy received by the system per ATP synthesis

We consider mode (iv) where the γ subunit rotates in the inverse direction even under the aqueous-solution condition that the overall reaction should be ATP hydrolysis.^75,76^ This rotation is driven by sufficiently strong external torque imposed on the γ subunit, leading to the occurrence of ATP synthesis as the overall reaction.

According to the experimental result reported by Toyabe and Muneyuki,^76^ the following equation holds:

*W*_ext_−*Q*=*W*~−*ΔG*, (S17)

where *W*_ext_, *Q*, and *W* denote the energy given to the system, energy lost as the heat, and energy received by the system per ATP synthesis cycle, respectively (i.e., these quantities are discussed by dividing them by the number of the ATP synthesis reactions which have occurred), and *ΔG*~−12 kcal/mol. *W*_ext_ is the external torque multiplied by 2π/3, and −*ΔG*>0 is the free energy required for the ATP synthesis reaction. In the stalled state, the number of normal rotations is equal to that of inverse rotations. If this number is 10 for an energy of *W*_ext,total_ given to the system, for example, the change in system free energy is zero, but −10*ΔG* required for the ATP synthesis reactions is given to the system through the external torque: *W*_ext_=*W*_ext,total_/10=−*ΔG*, *W*_ext_−*Q*=−*ΔG*, and *Q*=0.　An interpretation of Eq. (S17) is the following: Even when a large amount of energy is given to F_1_-ATPase, it receives only the amount required for the ATP synthesis (an increase in *W*_ext_ is followed by an equal increase in *Q* and *W*_ext_−*Q* remains constant) and −*ΔG*/*W*~1, leading to the ~100% efficiency which is surprising (Toyabe and Muneyuki, 2015). However, “F_1_-ATPase” in the aforementioned interpretation should be replaced by “the system” formed by not only F_1_-ATPase but also the aqueous solution. The system exchanges heat with the heat bath (i.e., the surroundings). If *Q* is regarded as the heat transferred to the heat bath keeping the system temperature constant, Eq. (S17) is simply a free-energy balance equation that is thermodynamically ordinary and −*ΔG*/*W*~1 is not surprising.

# Experimental evidence supporting our view on change in packing structure of F_1_-ATPase during ATP hydrolysis cycle

Crystal structures were solved for the catalytic dwell state of yeast F_1_-ATPase and the state after 16° rotation of γ subunit.^77^ In the catalytic dwell structure, AMP-PNP is bound to β_DP_ and β_TP_ and Pi is bound to β_E_ as illustrated in Figure S6. The ATP hydrolysis does not occur in β_DP_ during the 16° rotation, and the rotation is triggered by the dissociation of Pi from β_E_. We analyzed the packing structures of the two crystal structures: the packing efficiencies (PEs) of the three subcomplexes (or equivalently, the PEs of the three β subunits) and those of the α-β, α-γ and β-γ interfaces.^54^ AMP-PNP was replaced by ATP (ATP-Mg^2+^ is bound to β_DP_ and β_TP_).

We found the following:

(1) Upon the rotation, the PE of β_DP_ (and that of subcomplex 3) becomes looser, whereas the PEs of β_E_ and β_TP_ (and those of subcomplexes 1 and 2) become closer. Importantly, a decrease in the PE for one of the three β subunits is compensated with increases in the PEs for the other two β subunits. This result is suggestive that during the change from state (A) to an intermediate state between states (A) and (B) in Figure 7, there is no high free-energy barrier thanks to the compensation, keeping the water entropy almost constant. We believe that qualitatively the same compensation occurs during the change from state (B) to an intermediate state between states (B) and (C) in Figure 7.


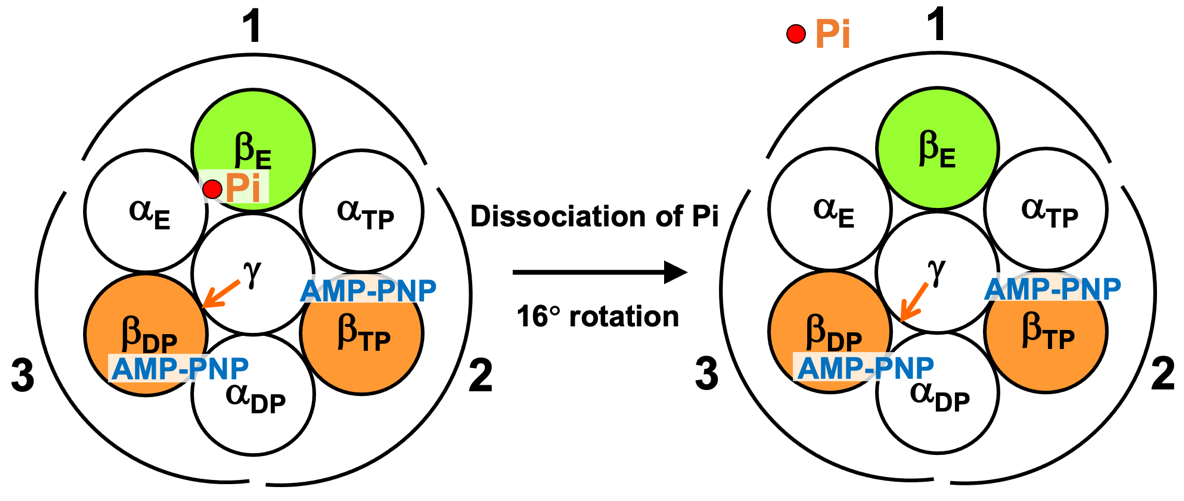
(2) The interfaces of α_DP_-β_DP_ and α_E_-β_E_ become less efficiently packed (i.e., more open) after the rotation, which is in agreement with the experimental observations.^78^

FIGURE S6

States of yeast F_1_-ATPase before and after 16° rotation of γ subunit. The 16° rotation is triggered by the dissociation of Pi from β_E_.

# Physical origin of the force for moving myosin along F-actin

In our view, the force generated by water for moving myosin along F-actin is primarily entropic in its origin, which is supported by the experimental data manifesting the following: (1) The changes in entropy and enthalpy upon the binding of myosin to F-actin are both positive^49,78^ and therefore the binding is entropy-driven; (2) the binding entropy (i.e., the change in entropy upon the binding) for myosin with ATP bound and F-actin is much smaller than that for myosin without ATP bound and F-actin;^49^ and (3) the binding affinity of myosin for F-actin is weakened at low temperatures^19^ whereas (4) it is enhanced by the addition of sucrose.^72^ Fact (2) is consistent with the experimentally known behavior that myosin can get detached from F-actin only after ATP binds to it. Fact (3), which originates from the weakening of the water-entropy effect (i.e., the weakening of the hydrophobic effect) at low temperatures,^3,13^ is associated with the cold denaturation of a protein.^3,18^ Fact (4) is exemplified by the enhancement of thermal stability of a protein, in which case the water-entropy effect becomes larger due to the sucrose addition.^23^

As for fact (4), Iwaki et al.^72^ experimentally studied the effect of sucrose addition on the movement of myosin V along F-actin (Sec. 1). At a sucrose concentration of 2 mol/L, the movement of myosin was stalled, but this is not because the viscous resistance force by water acting on the myosin head was made much stronger but because the myosin head could not be detached from F-actin due to too high an affinity of the myosin head for F-actin. (See Subsec. 2.11 in the recently published book^34^ as well.)

# Subjects to be theoretically investigated in further studies

We discuss the subjects to be theoretically investigated in the near future as follows.

(i) Since Orders (7) and (17) are consistent with the rotation mechanisms of F_1_-ATPase and V_1_-ATPase, respectively, the corroboration of Order (17) for V_1_-ATPase should lead to that of Order (7) for F_1_-ATPase as well. For Order (17), A(ATP)>A(ADP)>A has been proved in this study, and it is probable that A(ADP+Pi)>A(ADP) holds. The order that remains to be corroborated is A(ATP)>A(ATP(ATP-H_2_O))>A(ATP-H_2_O). ADP·AlF_4_ is different from ADP and ATP but much closer to ATP: It corresponds to A(ATP(ATP-H_2_O)) or A(ATP-H_2_O).^55,56^ In the succeeding study, we intend to show A(ATP)>A(ADP·AlF_4_)>A(ADP) using the three crystal structures of A_3_B_3_DF⋅2ATP, A_3_B_3_DF⋅2ADP·AlF_4_, and A_3_B_3_DF⋅2ADP^47,57,63^ which will be refined using all-atom MD simulations (see Subsec. 5.1). The corroboration of A(ATP)>A(ADP·AlF_4_)>A(ADP) is a reasonable alternative to that of A(ATP)>A(ATP(ATP-H_2_O))>A(ATP-H_2_O)>A(ADP). All of the issues to be pursued in further studies specified in Table 1 will then be resolved.

(ii) As described in Subsec. 6.2, even the structure of A_3_B_3_ (the complex with no nucleotides bound and without the DF subunit) is asymmetric. The physical origin of this interesting feature of V_1_-ATPase is to be investigated.

(iii) Taking at least one of the association patterns given in Table 3, we plan to calculate the changes in hydration entropy, hydration energy, and conformational energy (sum of protein and nucleotide intramolecular energies) and corroborate ⎪“change in hydration energy + change in conformational energy”⎪ << “change in hydration entropy (water-entropy gain)”, the dominance of the water-entropy effect.

(iv) In protein folding, as explained in Subsec. S1.3, though Δ*E*_ES_ is negative and very large, Δ*ε*_2-ES_ is positive and very large. They are compensating, and Δ*E*_ES_+Δ*ε*_2-ES_ is positive and small. Though Δ*E*_vdW_ is negative and large, Δ*ε*_2-vdW_ is positive and large. They are compensating, and Δ*E*_vdW_+Δ*ε*_2-vdW_ is negative and small. Δ*E*_ES_+Δ*ε*_2-ES_+Δ*E*_vdW_+Δ*ε*_2-vdW_ is positive and very small. Δ*S*_2-vdW_ can be neglected and Δ*S*_2-ES_ is very small. As a consequence, the thermodynamic quantities of hydration in process 1 dominate. Δ*ε*_1_ is positive and medium. The total-energy change, Δ*E*_ES_+Δ*ε*_2-ES_+Δ*E*_vdW_+Δ*ε*_2-vdW_+Δ*ε*_1_, is positive, implying that protein folding is entropy-driven. Δ*S*_1_ (~Δ*S*) is positive and very large. Δ*S*_C_ (*S*_C_ is the conformational entropy) is negative and large, but Δ*S*_1_+Δ*S*_C_ is positive and significantly large. Moreover, ⎢−*T*Δ*S*_1_⎢>Δ*ε*_1_. Taken together, protein is driven to fold by the entropic component of the hydrophobic hydration, *S*_1_. For the binding of an oncoprotein (MDM2) and the extreme N-terminal peptide region of a tumor suppressor protein p53 (p53NTD),^4^ the important quantities are as follows (*T*=298 K): Δ*E*_ES_=−513.9*k*_B_*T*, Δ*ε*_2-ES_=536.9*k*_B_*T*, Δ*E*_ES_+Δ*ε*_2-ES_=23.0*k*_B_*T*, Δ*E*_vdW_=−108.3*k*_B_*T*, Δ*ε*_2-vdW_=94.9*k*_B_*T*, Δ*E*_vdW_+Δ*ε*_2-vdW_=−13.4*k*_B_*T*, Δ*E*_ES_+Δ*ε*_2-ES_+Δ*E*_vdW_+Δ*ε*_2-vdW_=9.6*k*_B_*T*, Δ*ε*_1_=13.5*k*_B_*T*, −*T*Δ*S*_1_=−119.5*k*_B_*T*, −*T*Δ*S*_2-ES_=−12.8*k*_B_*T*, and −*T*Δ*S*_C_~59*k*_B_*T*. Therefore, an argument which is similar to that in the case of protein folding can be made. It follows that Δ*S*_1_ is an essential quantity in such processes as protein folding and protein-peptide binding. In any case, we have much experience in the incorporation of enthalpic components and conformational entropies of biomolecules for protein-peptide binding^4^ and protein folding/unfolding problems.^3,14^ In future studies on F_1_- and V_1_-ATPases, we will try to incorporate the enthalpic components and the conformational entropies of biomolecules for elaborating our theoretical model.

Studies in these directions are in progress at our laboratory.

In Figure 7, the 120° rotation splits into the two substep rotations in state changes (A)→(B) and (B)→(C). The rotation angle in (B)→(C) is larger than that in (A)→(B), because (B)→(C) includes the ATP binding by which a relatively larger state change is caused. For elucidating the 40° rotation in (A)→(B), at least the structure of state (B) is required. Very recently, Sobti et al.^79^ determined the structures of F_1_-ATPase in the catalytic-dwell and binding-dwell states which were supposed to be corresponding to states (A) and (B) in Figure 7, respectively, using cryo-electron microscopy. However, ADP and ATP were unexpectedly bound to β_E_ and β’_E_, respectively (i.e., three nucleotides were bound to the three β subunits, respectively, in the two structures), which is not in accord with the results from single-molecule experiments by Noji and coworkers.^80−83^ The reasons for the ADP and ATP bindings are unknown. Since the nucleotide binding should influence the packing structure to a significant extent, the two structures do not actually correspond to states (A) and (B) in Figure 7, respectively, and they are not suited to further theoretical analyses for elucidating why the angles of the two substep rotations are 40° and 80°, respectively. It is desired that the structure of state (B) be solved experimentally for deepening the understanding of the rotation mechanism.

Moreover, though we have shown that many of the fundamental characteristics of the rotation mechanisms of F_1_- and V_1_-ATPases can be elucidated by a theoretical model focused on the water-entropy effect, it can be improved by incorporating such physical factors as the electrostatics in future studies.

# References

^1^S. Hikiri, T. Hayashi, M. Inoue, T. Ekimoto, M. Ikeguchi, and M. Kinoshita, J. Chem. Phys. **150**, 175101 (2019).

^2^T. Yoshidome, M. Kinoshita, S. Hirota, N. Baden, and M. Terazima, J. Chem. Phys. **128**, 225104 (2008).

^3^M. Inoue, T. Hayashi, S. Hikiri, M. Ikeguchi, and M. Kinoshita, J. Mol. Liq. **317**, 114129 (2020).

^4^T. Yamada, T. Hayashi, S. Hikiri, N. Kobayashi, H. Yanagawa, M. Ikeguchi, M. Katahira, T. Nagata, and M. Kinoshita, J. Chem. Inf. Model. **59**, 3533 (2019).

^5^M. Kinoshita and T. Hayashi, Phys. Chem. Chem. Phys. **19**, 25891 (2017).

^6^M. Kinoshita, J. Chem. Phys. **128**, 024507 (2008).

^7^Y. Harano and M. Kinoshita, Biophys. J. **89**, 2701 (2005).

^8^M. Kinoshita, Front. Biosci. **14**, 3419 (2009).

^9^T. Yoshidome and M. Kinoshita, Phys. Chem. Chem. Phys. **14**, 14554 (2012).

^10^M. Kinoshita, Biophys. Rev. **5**, 283 (2013).

^11^H. Oshima and M. Kinoshita, J. Chem. Phys. **142**, 145103 (2015).

^12^M. Kinoshita, *Mechanism of functional expression of the molecular machines* (Springer Briefs in Molecular Science, 2016).

^13^S. Yasuda, K. Kazama, T. Akiyama, M. Kinoshita, and T. Murata, J. Mol. Liq. **301**, 112403 (2020).

^14^M. Inoue, T. Hayashi, S. Hikiri, M. Ikeguchi, and M. Kinoshita, J. Chem. Phys. **152**, 065103 (2020).

^15^T. Ikkai and T. Ooi, Biochemistry **5**, 1551 (1966).

^16^P. S. Niranjan, P. B. Yim, J. G. Forbes, S. C. Greer, J. Dudowicz, K. F. Freed, and J. F. Douglas, J. Chem. Phys. **119**, 4070 (2003).

^17^D. Foguel, M. C. Suarez, A. D. Ferrao-Gonzales, T. C. R. Porto, L. Palmieri, C. M. Einsiedler, L. R. Andrade, H. A. Lashuel, P. T. Lansbury, J. W. Kelly, and J. L. Silva, Proc. Natl. Acad. Sci. U. S. A. **100**, 9831 (2003).

^18^A. Pastore, S. R. Martin, A. Politou, K. C. Kondapalli, T. Stemmler, and P. A. Temussi, J. Am. Chem. Soc. **129**, 5374 (2007).

^19^S. Highsmith, Arch. Biochem. Biophys. **180**, 404 (1977).

^20^R. Mishra and R. Winter, Angew. Chem. Int. Ed. **47**, 6518 (2008).

^21^H. S. Ashbaugh and L. R. Pratt, Rev. Mod. Phys. **78**, 159 (2006).

^22^D. Myres, *Surfaces, interfaces, and colloids: Principles and applications* (Wiley-VCH, Berlin, 1999).

^23^H. Oshima and M. Kinoshita, J. Chem. Phys. **138**, 245101 (2013).

^24^S. Murakami and M. Kinoshita, J. Chem. Phys. **144**, 125105 (2016).

^25^S. Murakami, T. Hayashi, and M. Kinoshita, J. Chem. Phys. **146**, 055102 (2017).

^26^T. Hayashi, S. Yasuda, T. Škrbić, A. Giacometti, and M. Kinoshita, J. Chem. Phys. **147**, 125102 (2017).

^27^T. Hayashi, M. Inoue, S. Yasuda, E. Petretto, T. Škrbić, A. Giacometti, and M. Kinoshita, J. Chem. Phys. **149**, 045105 (2018).

^28^Y. Harano, T. Yoshidome, and M. Kinoshita, J. Chem. Phys. **129**, 145103 (2008).

^29^T. Yoshidome, Y. Harano, and M. Kinoshita, Phys. Rev. E **79**, 011912 (2009).

^30^H. Oshima, T. Yoshidome, K. Amano, and M. Kinoshita, J. Chem. Phys. **131**, 205102 (2009).

^31^S. Hikiri, T. Yoshidome and M. Ikeguchi, J. Chem. Theory Comput. **12**, 5990 (2016).

^32^A. Merlino, N. Pontillo, and G. Graziano, Phys. Chem. Chem. Phys. **19**, 751 (2017).

^33^T. Sumi and H. Imamura, Protein Sci. **30**, 2132 (2021).

^34^M. Kinoshita, *Mechanism of functional expression of F_1_-ATPase* (Springer Briefs in Molecular Science, 2021).

^35^M. Kinoshita, Chem. Eng. Sci. **61**, 2150 (2006).

^36^E. A. Meyer, R. K. Castellano, and F. Diederich, Angew. Chem. Int. Ed. **42**, 1210 (2003).

^37^E. Persch, O. Dumele, and F. Diederich, Angew. Chem. Int. Ed. **54**, 3290 (2015).

^38^P. Setny, R. Baron and J. A. McCammon, J. Chem. Theory Comput. **6**, 2866 (2010).

^39^F. Kamo, R. Ishizuka, and N. Matubayasi, Protein Sci. **25**, 56 (2016).

^40^Y. Togunaga, Y. Yamamori, and N. Matubayasi, J. Chem. Phys. **148**, 125101 (2018).

^41^M. Kinoshita and Y. Harano, Bull. Chem. Soc. Jpn. **78**, 1431 (2005).

^42^S. Mukherjee and A. Warshel, Proc. Natl. Acad. Sci. U. S. A. **112**, 2746 (2015).

^43^K. Y. Hara, H. Noji, D. Bald, R. Yasuda, K. Kinosita Jr., and M. Yoshida, J. Biol. Chem. **275**, 14260 (2000).

^44^M. Tanigawara, K. V. Tabata, Y. Ito, J. Ito, R. Watanabe, H. Ueno, M. Ikeguchi, and H. Noji, Biophys. J. **103**, 970 (2012).

^45^S. Vicatos, A. Rychkova, S. Mukherjee, and A. Warshel, Proteins **82**, 1168 (2014).

^46^A. Singharoy, C. Chipot, M. Moradi, and K. Schulten, J. Am. Chem. Soc. **139**, 293 (2017).

^47^M. Shekhar, C. Gupta, K. Suzuki, C. K. Chan, T. Murata, and A. Singharoy, ACS Cent. Sci. **8**, 915 (2022).

^48^T. Kodama, *Energetics of myosin ATP hydrolysis by calorimetry*, In *The role of water in ATP hydrolysis energy transduction by protein machinery*, M. Suzuki, ed., Part II, Chap. 7, pp. 103−111 (Springer Briefs in Molecular Science, Springer, 2018).

^49^T. Kodama, Physiol. Rev. **65**, 467 (1985).

^50^R. Alberty, Pure Appl. Chem. **66**, 1641 (1994).

^51^D. Voet and J. G. Voet, *Biochemistry* (3rd ed.), Sec. 22 in Chap. IV (John Wiley & Sons, New York, 2004).

^52^M. W. Bowler, M. G. Montgomery, A. G. W. Leslie, and J. E. Walker, J. Biol. Chem. **282**, 14238 (2007).

^53^T. Yoshidome, Y. Ito, M. Ikeguchi, and M. Kinoshita, J. Am. Chem. Soc. **133**, 4030 (2011).

^54^T. Yoshidome, Y. Ito, N. Matubayasi, M. Ikeguchi, and M. Kinoshita, J. Chem. Phys. **137**, 035102 (2012).

^55^A. J. Fisher, C. A. Smith, J. B. Thoden, R. Smith, K. Sutoh, H. M. Holden, and I. Rayment, Biochemistry **34**, 8960 (1995).

^56^R. I. Menz, J. E. Walker, and A. G. W. Leslie, Cell **106**, 331 (2001).

^57^K. Suzuki, K. Mizutani, S. Maruyama, K. Shimono, F. L. Imai, E. Muneyuki, Y. Kakinuma, Y. Ishizuka-Katsura, M. Shirouzu, S. Yokoyama, I. Yamato, and T. Murata, Nat. Commun. **7**, 13235 (2016).

^58^P. G. Kusalik and G. N. Patey, J. Chem. Phys. **88**, 7715 (1988).

^59^N. M. Cann and G. N. Patey, J. Chem. Phys. **106**, 8165 (1997).

^60^P. G. Kusalik and G. N. Patey, Mol. Phys. **65**, 1105 (1988).

^61^R. Roth, Y. Harano, and M. Kinoshita, Phys. Rev. Lett. **97**, 078101 (2006).

^62^A. D. MacKerell, Jr., D. Bashford, M. Bellott, R. L. Dunbrack, Jr., J. D. Evanseck, M. J. Field, S. Fischer, J. Gao, H. Guo, S. Ha, D. Joseph-McCarthy, L. Kuchnir, K. Kuczera, F. T. Lau, C. Mattos, S. Michnick, T. Ngo, D. T. Nguyen, B. Prodhom, W. E. Reiher, B. Roux, M. Schlenkrich, J. C. Smith, R. Stote, J. Straub, M. Watanabe, J. Wiórkiewicz-Kuczera, D. Yin, and M. Karplus, J. Phys. Chem. B **102**, 3586 (1998).

^63^S. Arai, S. Saijo, K. Suzuki1, K. Mizutani, Y. Kakinuma, Y. Ishizuka-Katsura, N. Ohsawa, T. Terada, M. Shirouzu, S. Yokoyama, S. Iwata, I. Yamato, and T. Murata, Nature **493**, 703 (2013).

^64^R. Rosenzweig and L. E. Kay, Annu. Rev. Biochem. **83**, 291 (2014).

^65^A. J. Wand and K. A. Sharp, Annu. Rev. Biophys. **47**, 41 (2018).

^66^H. Oshima, T. Hayashi, and M. Kinoshita, Biophys. J. **110**, 2496 (2016).

^67^M. K. Gilson, J. A. Given, B. L. Bush, and J. A. McCammon, Biophys. J. **72**, 1047 (1997).

^68^S Mukherjee, and A Warshel, Proc. Natl. Acad. Sci. U. S. A. **108**, 20550 (2011).

^69^K. Kitamura, M. Tokunaga, A. H. Iwane, and T. Yanagida, Nature **397**, 129 (1999).

^70^M. Kinoshita, Biophys. Physicobiol. **18**, 60 (2021).

^71^M. Maroncelli, J. Mol. Liq. **57**, 1 (1993).

^72^M. Iwaki, K. Ito, and K. Fujita, *Single-molecule analysis of actomyosin in the presence of osmolyte*, In *The role of water in ATP hydrolysis energy transduction by protein machinery*, M. Suzuki, ed., Part III, Chap. 15, pp. 245−256 (Springer Briefs in Molecular Science, Springer, 2018).

^73^K. Amano, T. Yoshidome, M. Iwaki, M. Suzuki, and M. Kinoshita, J. Chem. Phys. **133**, 045103 (2010).

^74^M. Kinoshita, *Functioning mechanism of ATP-driven proteins inferred on the basis of water-entropy effect*, In *The role of water in ATP hydrolysis energy transduction by protein machinery*, M. Suzuki, ed., Part III, Chap. 18, pp. 303–323 (Springer Briefs in Molecular Science, Springer, 2018).

^75^S. Toyabe, T. Watanabe-Nakayama, T. Okamoto, S. Kudo, and E. Muneyuki, Proc. Natl. Acad. Sci. U. S. A. **108**, 17951 (2011).

^76^S. Toyabe and E. Muneyuki, New J. Phys. **17**, 015008 (2015).

^77^V. Kabaleeswaran, N. Puri, J. E. Walker, A. G. W. Leslie, and D. M. Mueller, EMBO. J. **25**, 5433 (2006).

^78^T. Katoh and F. Morita, J. Biochem. **120**, 189 (1996).

^79^M. Sobti, H. Ueno, H. Noji, and A. G. Stewart, Nat. Commun. **12**, 4690 (2021).

^80^R. Yasuda, H. Noji, K. Kinosita, Jr., and M. Yoshida, Cell **93**, 1117 (1998).

^81^R. Yasuda, H. Noji, M. Yoshida, K. Kinosita, Jr., and H. Itoh, Nature **410**, 898 (2001).

^82^K. Adachi, K. Oiwa, T. Nishizaka, S. Furuike, H. Noji, H. Itoh, M. Yoshida, and K. Kinosita, Jr., Cell **130**, 309 (2007).

^83^R. Watanabe, R. Iino, and H. Noji, H., Nat. Chem. Biol. **6**, 814 (2010).
